# The role of renewables for rapid transitioning of the power sector across states in India

**DOI:** 10.1038/s41467-022-33048-8

**Published:** 2022-09-21

**Authors:** Ashish Gulagi, Manish Ram, Dmitrii Bogdanov, Sandeep Sarin, Theophilus Nii Odai Mensah, Christian Breyer

**Affiliations:** 1 LUT University, Yliopistonkatu 34, 53850 Lappeenranta, Finland; 2 Wärtsilä Corporation, Hiililaiturinkuja 2, 00180 Helsinki, Finland

**Keywords:** Energy economics, Energy supply and demand, Renewable energy, Energy modelling

## Abstract

Recent events like heatwaves and abnormal rainfall are a glimpse of the devastating effects of human induced climate change. No country is immune to its effects, but a developing country like India is particularly vulnerable. This research, for the individual states of India, explores the technical feasibility and economic viability of a renewable transition pathway for the power sector. Based on the assumptions of this study, we show that a renewables-based power system by 2050 is lower in cost than the current  coal dominated system, has zero greenhouse gas emissions and provides reliable electricity to around 1.7 billion people. Electricity generation will be based on solar PV, wind energy, and hydropower, while batteries and multi-fuel reciprocating internal combustion engines based on synthetic fuels provide the required flexibility to the power system. This transition would address  multiple imperatives: affordability, accessibility, and sustainability without compromising economic growth.

## Abstract (web summary)

A new study assesses the feasibility of a fully renewable based power system by 2050 across India, finding this option to be cost competitive with the status quo and with zero GHG emissions.

## Introduction

Recent changes in the extremity and abnormality of weather events like heatwaves in the northern hemisphere and extreme rainfall in Europe and Asia have renewed focus on climate change^1–4^. These extreme and abnormal weather-related events are felt far and wide, as no country is immune to their  devastating effects, but a developing country like India is more vulnerable. India has its own share of extreme and untimely rainfalls, heatwaves and droughts, which are growing by every passing year^5–7^, a result of human-induced climate change. The consequences are extreme; socially and financially. The latest climate report from the Intergovernmental Panel on Climate Change (IPCC) finds that ‘it is now unequivocal that human-caused emissions from burning fossil fuels are responsible for recent warming’^5^. Though countries have ratified the Paris Agreement and pledged their Intended Nationally Determined Contributions (INDCs), recent climate events show that more ambitious targets are needed. Therefore, the first and foremost step is a shift away from the dependence on fossil fuels, especially in the power sector towards renewable energy at a faster rate than ever.

In this context, India’s path towards achieving the 1.5 °C target needs to be in synergy with its development imperatives; energy affordability and accessibility, mitigating air pollution, while maintaining rapid economic development^8^. Even though, historically, India has had lower per capita emissions than other developed countries, it has been at the forefront of the global climate debate^9^. In this regard, India has committed to reducing the greenhouse gas (GHG) emissions intensity of its GDP by 33-35% below 2005 levels and achieving 40% of cumulative installed power generation capacity from non-fossil sources by 2030^10,11^. To further its global climate commitment and leadership, it has pledged at the UN Climate Summit in 2019 a target of 450 GW of renewable energy (RE) to be achieved by 2030^12^. However, the challenge for India going forward will be to align its renewable growth trajectory with its social and economic development priorities. It is vital to set long-term goals and envision a net zero emission energy system across the country, which will not only ensure economic benefits but also place India in a position of global climate leadership. In recent years, India has taken remarkable strides in reforming its power sector, with electricity shortages declining and an electrification of 99.9% of the households across the country^13^. However, there is still a long way to go in reaching the standards of the developed world in terms of reliability and per capita consumption. The per capita electricity consumption within the Indian states and union territories differs a lot but is less than the global average of almost 3000 kWh^14^. As of 2020^15^, the average per capita electricity consumption in India is only 1200 kWh. A dynamic growth in future electricity demand is projected over the coming years, escalated by the growing economy and end-use services^16^, despite the government’s efforts to pursue strong energy efficiency standards^17^. This research focuses only on the power sector, while other sectors such as heat and transport will further increase the electricity demand. In its INDC, it is mentioned that ‘half of the India of 2030 is yet to be built’^11^. Thus, India’s power generation choices will have implications on its long-term emissions locally and globally.

Historically, the power sector in India has been the largest contributor to energy-related GHG emissions. The dependence on low-quality coal used in highly inefficient power plants has resulted in air pollution, predominant in cities and aggravating other environmental issues^18^. Additionally, many of these coal power plants are operating at lower plant load factors (PLF), thus reducing their profitability and compounding their already dwindling financial returns^14^. Already, solar PV-based electricity generation ranges between 1.99 and 2.36 INR/kWh (24.8–29.5 €/MWh), as compared to electricity from domestic coal-fired power plants costing 3.5–5 INR/kWh (43.7–62.5 €/MWh). To complicate the matter further, under-construction coal power plants will add to the financial burden of already cash-strapped distribution companies, as these inflexible assets cannot compete with low-cost solar-based electricity^19^. On the other hand, private investors are shying away from coal investments due to the associated risks and are shifting towards sustainable technologies^20^. This has resulted in many coal power projects being scrapped or abandoned^21^.

Another issue with coal power plants is the use of freshwater for cooling. India, currently, is placed 13^th^ among the world’s ‘extremely water stressed countries’ and most of its states are facing depleting freshwater resources. It is projected that two-thirds of the country’s power plants will face high water stress by the end of 2030^22^. About 40% of coal power plants are located in these water-stressed areas across the country, while the total water requirement for thermal cooling makes up more than half of the domestic water demand^23,24^. Consequently, water shortages or drought-like situations have resulted in thermal power plant shutdowns, resulting in a loss of 1.4 bUSD (1.3 b€) between 2013 and 2016, due to lack of fresh water available for cooling^25^. With the population predicted to grow, there will be an increase in irrigation requirements, which will put tremendous pressure on already scarce water resources^26^. Competing uses of freshwater for vital irrigation and electricity generation in thermal power plants cause immense challenges for decision-makers. These factors, together with India’s ambitious climate change goals and record low solar and wind energy prices, have made thermal power plants unviable in the long term, with high risks of being stranded assets. Therefore, with a view on these impacts, in this study, it is assumed that there will be no new coal or fossil fuel-based power plants built in the future to focus on a least cost and best policy scenario.

India is one of the countries that has been aggressively pursuing renewable capacity installations. For the past few years, significant growth has been observed in solar and wind installations. To put this in context, solar capacity has grown 13 times in the last six years^27^, reaching approximately 45.6 GW by August 2021^28^. This growth aptly reflects the government’s plan to cash in on the declining costs and significant solar potential available in the country. Even in its integrated energy policy, the government has put forth that solar energy is the way forward for India^29^. This indicates that the trend of growing renewable capacity installations will continue amid sharp falling costs and supportive policies from the central as well as state governments. Saraswat and Digalwar^30^ assessed the sustainability of various energy resources in India, with empirical investigations and validation of sustainability indicators. Solar energy ranked as the most sustainable energy resource, followed by wind energy, while the least sustainable energy resources were thermal and nuclear. As highlighted by Child et al.^31^, the benefits of utilising renewables go beyond the energy sector, and solar and wind are the foremost technologies to achieve sustainability goals.

**Fig. 1:**
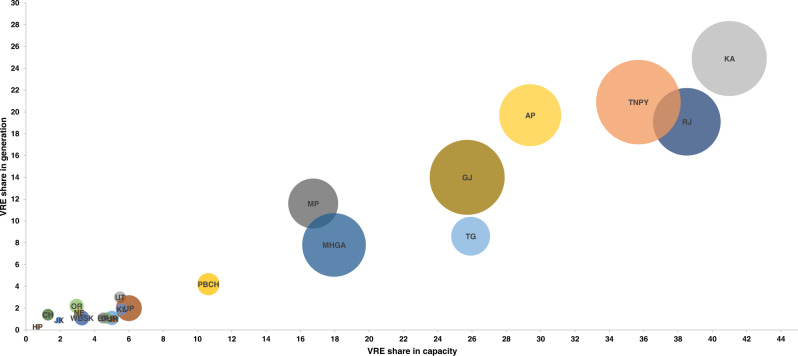
Variable Renewable Energy (VRE) share by state in capacity and generation, 2019^97^. The x and y axis represent VRE share of capacity and generation in each state’s total capacity and generation respectively. The bubble size represents the share of electricity generation by VRE in each state with respect to the total electricity generation in India. Tamil Nadu has the highest share of VRE in total India generation. Karnataka has the highest share of capacity and generation among all the states. Abbreviations: AP Andhra Pradesh, BR Bihar, CH Chhattisgarh, DL New Delhi, GJ Gujarat, Daman and Dadra, HP Himachal Pradesh, HR Haryana, JH Jharkhand, JK Jammu-Kashmir, KA Karnataka, KL Kerala, MHGA Maharashtra and Goa, MP Madhya Pradesh, NE North Eastern states, OR Odisha, PBCH Punjab and Chandigarh, RJ Rajasthan, TG Telangana, TNPY Tamil Nadu and Puducherry, UP Uttar Pradesh, UT Uttarakhand, WBSK West Bengal and Sikkim.

However, to achieve the ambitious target of 450 GW of renewables, more needs to be done in some of the states and policies need to be aligned with the ambitions of the central government. The role of renewables in electricity generation is highly variable within the states of India. For example, the share of renewables in electricity generation from renewable energy-rich states like Andhra Pradesh, Gujarat, Karnataka, Kerala, Maharashtra, Madhya Pradesh, Punjab, Rajasthan, Tamil Nadu and Telangana is considerably higher than the national average of 8.2%^17^. Figure 1 shows the share of solar and wind electricity in total generation across all major states of India in 2020. The states of Karnataka, Tamil Nadu, and Rajasthan have considerable generation from solar and wind, while other states are still lagging in capacity and generation. Clearly, action will be required at both the state and national level to achieve the goals

As India plans to achieve its ambitious economic goals and climate change targets, the power sector assumes an important role, as decarbonization of the power sector is key for reducing CO_2_ emissions by mid-century. According to Bistline et al.^32^, power sector decarbonization will play a vital role in the complete decarbonization of the energy system through direct electrification of the processes and indirect electrification – electricity derived fuels. Likewise, a recent study on an energy transition pathway for India acknowledges the huge role of electricity as a key vector in final energy demand to achieve net-zero emissions by 2050^33^. As a result, failure to deeply decarbonize the power sector before mid of this century will seriously jeopardize ongoing global climate mitigation efforts^34^.

Various long-term transition studies on the emission reduction pathways for India have been conducted. Most of these studies, however, first, focus only on a national level^35^, second, lack a high temporal and spatial resolution of resources and power demand^36,37^, third, contain no or limited storage and flexibility options^38^, fourth, lack a transition pathway, showing how the current system will ‘transition’ towards a system with high shares of renewables^39^, and finally, consider limited share of renewable penetration^39,40^. Some of the key studies such as WWF and TERI^41^, Teske et al.^42,43^, Jacobson et al.^44^, Lawrenz et al.^36^, Gulagi et al.^45^ and Bogdanov et al.^46,47^, consider 100% renewable energy penetration, however, they lack in one or the other aspects mentioned above.

Considering the importance of the power sector, this study explores a rapid transition pathway for the power sector of India in a resolution of states from the current power system till 2050 in a 5-year time interval towards integrating large shares of renewables. There is a clear need for a transition pathway of the power sector beyond India’s target of 2030. This paper presents a cost-optimal transition pathway integrating various generation options, storage technologies and interstate transmission to meet the hourly power demand for an entire year. This paper answers two important questions, first, is a 100% renewable energy-based power system technically possible and is it the least cost option in 2050? Second, how much and what are the generation capacities, storage, and flexibility requirements on a state and national level during the transition?

To explore the power sector transition pathway, India was divided into 22 states/regions (henceforth, the individual states and the states combined together will be called as ‘states’), which are grouped into four major regional grids (Northern, Western, Southern and a combined Eastern and North Eastern) that are further interconnected to form a national transmission network, as highlighted in Methods Fig. 9. The North Eastern grid is combined with the Eastern grid due to the relative size of its power system in the total electricity demand. Similarly, states in the Northeast of India are combined into one region of ‘Northeast’ and the Union Territories except Delhi, are combined with the adjacent states.

## Results and Discussion

### Capacity expansion during the transition

**Fig. 2:**
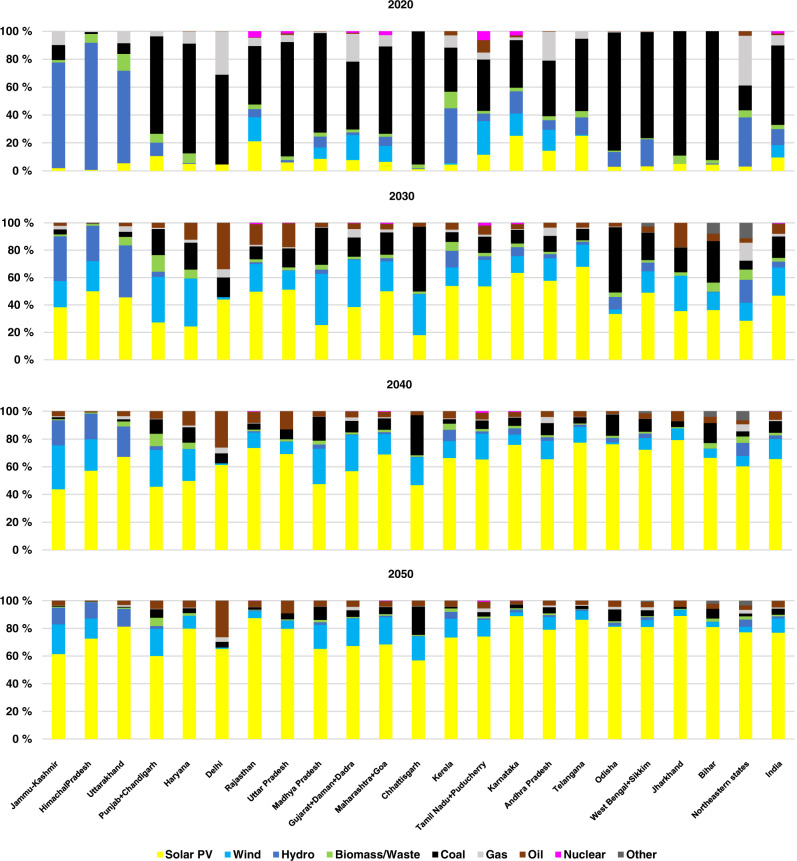
State-wise installed capacity share of different technologies in 2020, 2030, 2040 and 2050 for the individual states and aggregated all India. Solar PV capacity increases in all the states during the transition and is the main source of electricity generation, with a share of about 77% in the total installed capacity across India in 2050.

The cost optimal electricity generation capacities, which satisfy hourly demand in each of the regions are summarised in Fig. 2. The results show significant growth in optimal capacities of solar PV and wind power across all the states.

During the first decade of the transition, significant growth in solar PV capacities is observed in the larger states of Uttar Pradesh (82 GW) and Maharashtra (78 GW), a reflection of excellent solar resource availability and huge capacities required as replacement for decreased coal generation to satisfy the growing power demand. However, the highest average annual growth rates of solar PV installations are observed in Himachal Pradesh (228%), Jammu and Kashmir (125%) and Delhi (111%) in the Northern grid, Kerala (122%) in the Southern grid, West Bengal (121%) and Jharkhand (102%) in the Eastern grid. As hydropower is seasonal and increasing its capacity is comparatively expensive and time-consuming, the northern states, dependent on it, start investing and building solar PV at a faster rate than other states. On the other hand, the Eastern states dependent on coal, start investing in new solar PV capacities as it is the cheapest source of new electricity, saving on carbon emission costs for these states and corresponding GHG emissions. Growth in rooftop PV installation is observed across all states, particularly in Delhi, where land area is limited, and a large potential for rooftop PV is available. During the same period, wind resource-rich states observe the largest increase in wind energy installations. In absolute capacities, Gujarat (38 GW) and Maharashtra (30 GW) install wind turbines due to the availability of excellent wind resources. Installation of wind capacities is also observed in other states like Himachal Pradesh, Punjab, Haryana, and Uttar Pradesh, where the current installed capacities are negligible. Newer turbines with higher hub heights increase the capacity factors at these locations to make them more cost-competitive compared to other fossil and renewable sources of electricity generation. Additionally, during this period, as batteries are yet to be cost competitive and the dependence of solar PV on batteries to supply night-time demand, enables wind energy to see the highest growth.

From 2030 onwards, solar PV has a steady average annual growth rate of 35% across the states of India, as solar PV supported by batteries dominate the installed capacities, reaching almost 3000 GW by 2050. On the other hand, annual growth in wind capacities slows down during this period due to  better cost competitiveness of solar PV. With excellent resource availability across the length and breadth of India and a continuous decrease in cost, solar PV emerges as the major source of electricity generation in all the states in 2050, as seen from the Supplementary Information Fig. 5. Total wind capacity in the country by 2050 is about 410 GW. Regions with good exploitable hydropower potential, like the Northern and Eastern states, will see the maximum growth in hydropower capacities during the transition. Detailed data on installed capacities for each of the states for all generation technologies till 2050 in every 5-year interval is given in Supplementary Information Table 5.

Shares of fossil fuels and coal decline through the transition, with installed capacities of coal at risk of becoming stranded assets. These coal power plants have very low full load hours during the transition years, as the share of renewables increases, which will lead to reduced revenues and profitability. At the same time, if these coal power plants were made to operate flexibly, additional new investments would be required^17^. These additional investments should be compared, first, against other flexibility sources such as batteries, multi-fuel reciprocating internal combustion engines (ICE) and grids, which can support very high shares of VRE with faster ramp rates, and second, against climate change goals. Even retrofitting old coal power plants to operate flexibly will result in emissions. From the results, gas turbines and reciprocating multi-fuel ICE are installed by 2030 to provide flexibility to the system, which is already over 60% renewable energy based. These flexible generation sources ramp up and down, providing instantaneous demand cover, especially for the evening peaks. The increase in utilisation and capacity of interstate transmission networks adds an additional dimension of flexibility to the power system. Expansion of the transmission system smooths out the resource variability and provides access to low-cost electricity from other states^48^. As a result, local storage requirements and curtailment are reduced. The total grid capacity increases to 308 GW for a full renewable energy-based power system in 2050. Grid capacity expansion of all the transmission lines considered in this study for every 10-year period of the transition is given in Supplementary Information Table 6. On the other hand, it is assumed that as the inter-state transmission grid grows during the transition, simultaneously, necessary upgrades and improvements are made within each state’s grid network, as low cost electricity is available to each of the end-users.

### Electricity generation during the transition

**Fig. 3:**
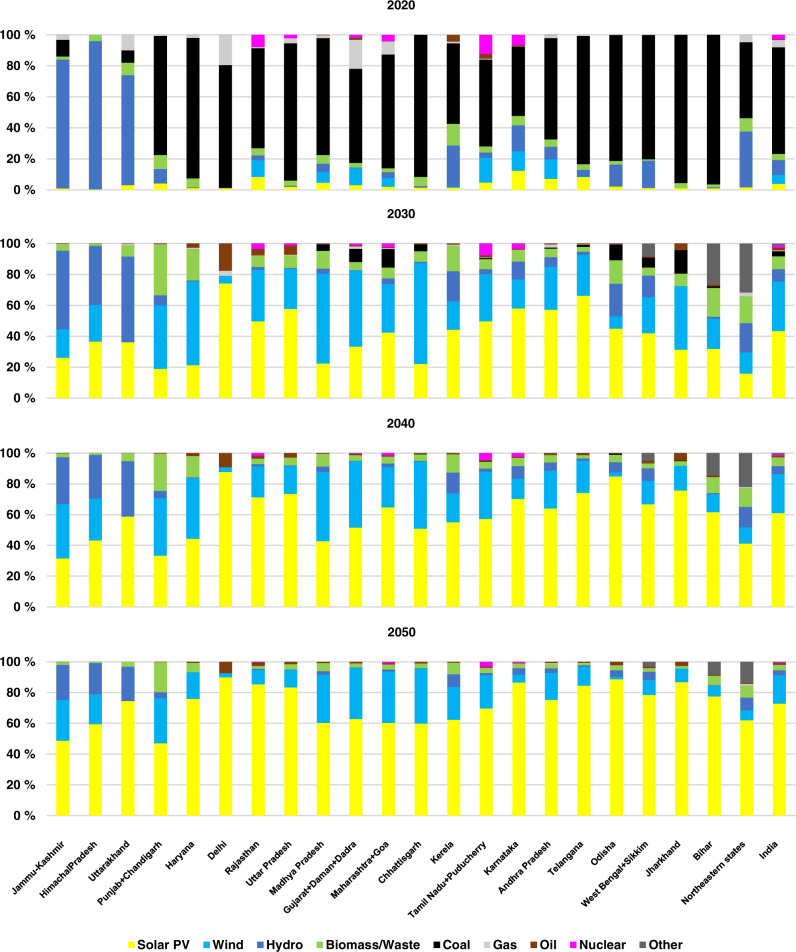
Contribution of different technologies to electricity generation in 2020, 2030, 2040 and 2050 for the individual states and aggregated all India. Transition from a coal-based to renewable energy based power system is rapid during the first decade. Electricity generated from solar PV has a share of about 73% in the total electricity generation across India in 2050.

The cost-optimal contribution of different generation sources in all states across India is illustrated in Fig. 3. The share of coal in electricity generation decreases across most of the states by more than 60% in 2030. Notably, more than 80% decrease is observed in Punjab, Haryana, and Delhi in the Northern grid, Kerala, and Karnataka in the Southern grid and Northeastern states. However, states with high electricity demand, such as Gujarat and Maharashtra, and states within the traditional coal belt; Odisha, West Bengal and Jharkhand, still have a considerable share of coal generation in comparison to the rest of the states in India. On a national level, beyond 2020, as the share of coal continues to drop, first the share of wind energy in 2025 (27%) and then solar PV in 2030 (43%), increase in total electricity generation, as they become more cost competitive. This is also observed at the individual state level, where, first, electricity generation from wind energy picks up due to its high capacity factors and its availability at night. However, after 2030, as the cost competitiveness of hybrid PV-battery systems increases, solar PV will account for  the largest share of electricity generation. Round-the-clock Power Purchase Agreements (PPAs) are already on the rise across different parts of India to capture the cost decrease of hybrid PV-battery power solutions and provide night-time demand^49^. The higher share of solar electricity generation could enhance the resource complementarity across the states in an interconnected power system, thus neutralising the effects of the monsoon season^48^. On a national level in 2050, the major contribution to total power production are from solar (73%), wind (19%), with hydropower (3%) and nuclear power (0.4%) complementing VRE.

Multi-fuel ICEs, in 2050, have a share of 1.1% in electricity generation, driven by higher efficiency and lower cost, while having full load hours of over 800, mainly utilised for peak supply and balancing. Detailed generation data from different technologies is provided in the Supplementary Information Table 9.

It is quite evident that the Indian power sector is undergoing a rapid transition away from coal towards solar PV as the prime source of electricity generation as electricity from solar PV is the least cost. The trends during the last few years with record low tariffs across the country are already disrupting the economics of the power sector. With low-cost storage solutions, this trend is expected to be further amplified.

### Storage deployment during the transition

**Fig. 4:**
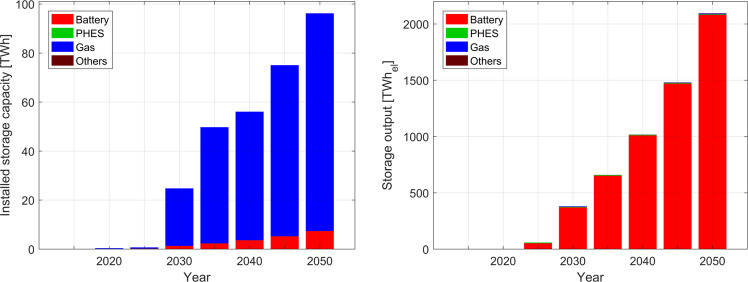
Aggerated storage capacity (a) and storage output (b) during the transition from 2015 to 2050 across India. The installed storage capacities are based on gas storage and the output is based on batteries, which is a consequence of structurally different charge-discharge cycles of short-term and seasonal storage technologies.

Supplementary Information Table 9 summarises the installed capacities and output during the transition in a cost-optimal power system for all storage technologies considered. The table shows that storage plays a vital role in enabling a smooth and secure hourly power supply across all the states during the transition. As of 2020, pumped hydro energy storage (PHES) is the only storage option that is available and used, albeit in only some of the states. However, the installed capacity and output is low and future projects have been stalled for various reasons, such as social and environmental^50^. During the transition, cost-optimal investments are made in batteries and gas storage on a large scale rather than PHES. Large scale storage requirements start in 2030.  However, this could very well take shape earlier with the right policy framework and incentives. In this research, storage capacities are initiated when the capacity share of renewables is more than 60%. Batteries perfectly complement the large share of solar PV in the generation due to their modularity, finally forming utility-scale hybrid PV-battery systems, while gas storage is used seasonally. The installed electricity storage capacity increases from about 22 TWh in 2030 to around 95 TWh by 2050, as shown in Fig. 4. Utility-scale and prosumer batteries contribute to a major share of the electricity storage output, with more than 98% by 2050, due to their low cost and high round trip efficiency, as diurnal storage requirements increase considerably by 2050.

On the other hand, gas storage, which is e-methane produced via the power-to-gas process, has large capacities but very few discharge cycles as compared to batteries. In a power-to-gas process, renewable electricity is used to capture carbon dioxide (CO_2_) from the air using direct air capture units and in the process of electrolysis, separating hydrogen (H_2_) from water. In the next step, these two gases are combined in a methanation process to produce synthetic methane (e-methane). The low capex of gas storage results in large capacities being installed during the transition, but only contributes to the vital seasonal storage through the transition. On a national level, it plays an important role when solar resource is at its lowest. Gas storage discharges slowly over the late monsoon and winter periods and is completely discharged till the end of winter. The excess electricity generated during the summer months is used to produce e-methane and charge the gas storage. Gas storage is completely charged till the end of summer. Hydropower reservoirs are charged completely during the monsoon and, similar to gas storage, provide complementarity to solar and wind generation but are mainly used for seasonal balancing. The state-of-charge (SoC) profiles for 2050 are provided for batteries, gas storage, and hydropower reservoirs in Supplementary Information Fig. 4.

**Fig. 5:**
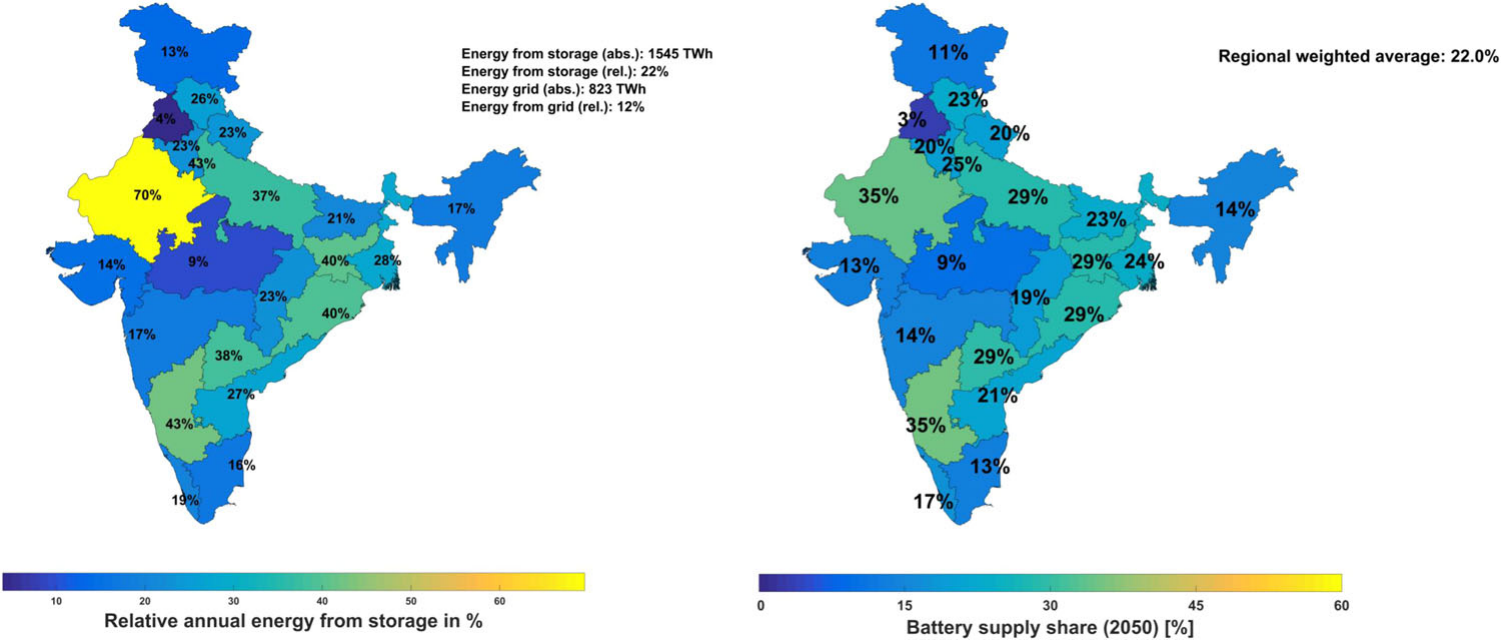
Regional distribution of relative storage output in individual states’ electricity generation (a) and battery supply share (b) in 2050. The aggregated average storage supply share is 22% of the total electricity generated, while the battery supply share is 99% of the total storage output.

On a regional level, in a fully renewable energy system in 2050, the storage capacities are well distributed across the regions of India. The installed storage capacities are dominated by gas storage that is mainly to provide seasonal storage, while the output is dominated by utility-scale and prosumer batteries (refer to Supplementary Information Fig. 6). Figure 5 shows the share of storage output in electricity generation across each region. Rajasthan has the largest share, with 70% of storage output in electricity generation among all the states. Given its geographic location with continuous and cheap solar availability throughout the year, batteries are needed on a diurnal cycle, while gas storage acts as an additional source of flexibility for balancing mainly seasonal unavailability of solar energy in events such as sandstorms. At the national level, battery output contributes an average of over 35% of electricity generation in 2050, as shown in Fig. 5. Rajasthan and Delhi have the largest shares of battery discharge in each of the regions’ total electricity generation. Utility-scale batteries form the major share in Rajasthan, while in Delhi it is prosumer batteries that are installed. Both reflect the type of solar PV capacity installed in these states.

Electrolysers play a vital role in the production of hydrogen during the transition of the power system across the states of India and reach an installed capacity of 407 GW_el_ in 2050. The major capacities are in the solar-rich states of Rajasthan, Karnataka and Uttar Pradesh, with minor capacities in the rest of the states across the country, as shown in Supplementary Information Fig. 5. Electrolysers not only produce hydrogen, which is a fuel as well as feedstock for the production of e-fuels, but also provide crucial flexibility to the power system through the transition.

### Import and Export of electricity in 2050

In a cost-optimised power system across India, transmission and distribution play a vital role in mitigating the variability of renewable resources. Thus, all states benefit from reduced investments in storage and other flexibility options while at the same time reducing the overall system costs. A strong regional grid is vital for all states to benefit from the low-cost renewable energy resources across the entire country. The power transmission capacity increases by more than six times from 2020 to 2050, as shown in Supplementary Information Fig. 8. The interregional exchange of electricity across India in 2050 is shown in Supplementary Information Fig. 9. On a seasonal scale, grid utilisation is predominantly high during the monsoon season^48^, while on a daily and weekly basis, high utilisation (hourly electricity transfer/(grid capacity·8760 h)) is observed during the morning and night hours. During a regular day, with good solar resource availability across the country, the least utilisation is observed during the noon hours, as direct electricity is used to satisfy the demand (refer to Supplementary Information Fig. 8).

Himachal Pradesh (227 TWh), Rajasthan (103 TWh) and Karnataka (116 TWh) are major net exporters of electricity, while Punjab and Chandigarh (116 TWh), Delhi (122 TWh), Maharashtra and Goa (110 TWh) and Tamil Nadu and Puducherry (116 TWh) are major net importers. Export states have excellent low-cost renewables-based electricity generation, particularly solar, wind and hydropower, which are exported, thus reducing the overall cost of storage and curtailment in these export states. Delhi, one of the largest populated cities by 2050, but with limited area and renewable energy resources, depends on neighbouring states to satisfy its electricity demand in 2050. The transmission line between Delhi and Haryana has the highest utilisation of 79% through the entire year, supplying about 121 TWh of electricity. These electricity imports play a crucial role in ensuring a steady supply of electricity throughout the year.

**Fig. 6:**
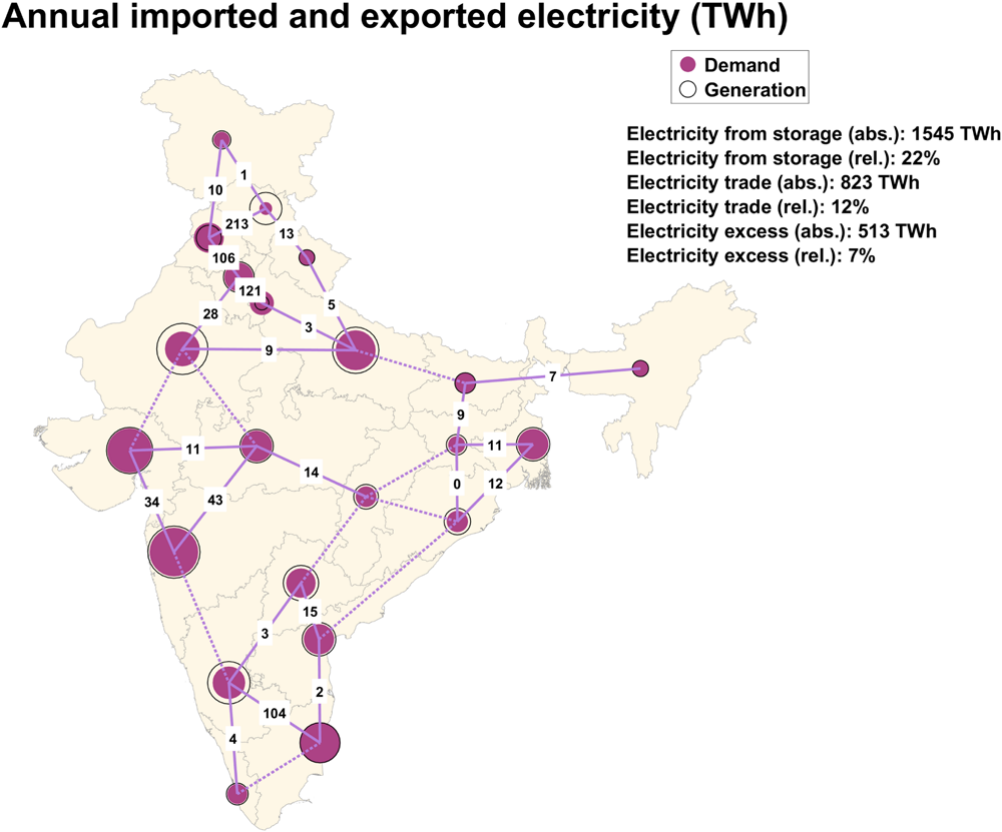
Inter-regional electricity exports and imports across India in 2050. The annual net exchange of electricity across India is around 823 TWh, which is 12% of the electricity generated in 2050.

The share of inter-state traded electricity reaches about 12% of the total generation in 2050, clearly indicating that the majority of electricity demand across the individual states is supplied within the respective states. This implies that despite the overall interconnectedness of states across the country, each state utilises locally available renewable resources to a large extent, ensuring robust power systems even at the state level. Figure 6 gives detailed information on the power exchange between states in 2050. Supplementary Information Tables 6-8 gives information on the capacity, electricity exchange and utilisation of each transmission line respectively.

### Seasonal power system analysis

A fully renewables-based power system across India has distinctive operational characteristics, which vary according to the states and seasonal patterns. Two important and distinctive seasonal variations, summer and monsoon, are considered to show the operational characteristics of a fully renewable energy based power system across India in 2050. Figure 10 in the Supplementary Information shows a representative week in summer and the monsoon season for an aggregated all India power system.

During the summer period, the major generation is from solar PV, complemented by wind energy. Significant curtailment (shown as excess) of solar and wind energy is seen on a daily basis. In the summer months, hourly curtailment can be as high as 33% of VRE-generated electricity. However, when integrated over an entire year, overall curtailment is down to 8.7%. This curtailment of electricity can be reduced by an integrated energy system, enhanced by the coupling of heat, transport and industrial sectors^51^. Storage plays an important role, especially, batteries, which are used on a daily basis, charging during the day and discharging during the evening and night hours to meet peak consumption, as highlighted in Fig. 10 of the Supplementary Information.

During the monsoon period, solar PV generation decreases, while wind generation increases and becomes the main source of electricity generation. Notably, excess electricity generation also decreases. Other renewable energy sources such as hydropower and dispatchable bioenergy support the lack of solar PV and wind generation. Reciprocating multi-fuel ICE are utilised in periods of low VRE generation, especially at the beginning of the week when wind generation is low and when solar generation is also low in the mid and end of the week, as shown in Supplementary Information Fig. 10.

Imports and exports of electricity between states of the country play a vital role in the monsoon season, while electricity exchange is rather limited in summer. The amount of excess electricity is lower in the monsoon season as compared to the summer season.

### Implications on costs and investments during the transition

**Fig. 7:**
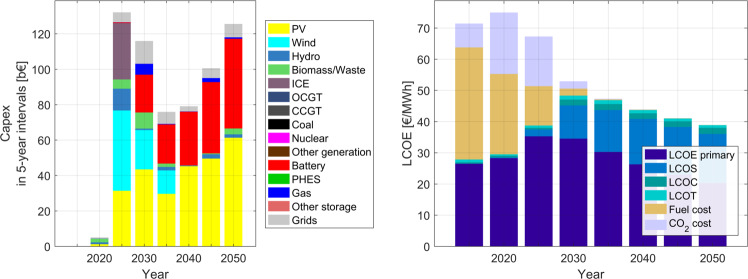
Capital expenditures for 5-year intervals (a) and levelised cost of electricity (b) during the energy transition from 2015 to 2050 across India. The highest investments take place in 2025, when the system needs to invest the most in building a new renewable energy based power system, as fossil fuels based technologies are decommissioned and restrictions on new installations. A fully renewable energy based power system in 2050 is cheaper in cost than the current fossil fuel based system.

The operating costs of the entire power system, including capital investments, operational expenditures, fuel costs, grid expansion costs and CO_2_ emission costs during the transition are given in Fig. 7. On a national level, capital expenditures increase through the transition, with wind and reciprocating multi-fuel ICE, and later with solar and batteries being dominant. During the initial years, wind energy, due to its cost competitiveness and higher capacity factors, and solar PV are installed. However, after 2030, solar PV and batteries will become cost competitive to other generation sources, due to rapidly decreasing costs. Investments in building new transmission lines start as early as 2025 and 2030, to provide the required flexibility to a rapidly changing power system.

The levelised cost of electricity declines from around 71 €/MWh in 2020 (includes CO_2_ emissions costs) to around 38 €/MWh by 2050 (refer Fig. 7) and is increasingly dominated by capital costs as fuel costs continue to decline through the transition period, which could mean increased self-reliance in terms of energy for India by 2050. Notably, the levelised cost of a fully renewable power system decreases by 46% compared to a system with 70% coal generation. Even without CO_2_ emission costs^52^, the decrease is about 30%. This indicates that a rapid transition of the Indian power system is simply a case of sound economics, but with additional benefits of reducing air pollution, corresponding health costs and creating jobs, which translate to further economic gains^53,54^. A steady growth in capital investments in the power sector indicates that fuel imports into the country and the respective negative impacts on trade balances will fade out through the transition, giving rise to increasing energy security.

The average cost across India is an accurate representation of the cost of the power sector, as effective cooperation among the states in terms of generation, transmission and storage enables a least-cost power system for India as well as the individual states. Direct investments, power purchase agreements (PPAs) for round-the-clock supply across the different states from central and state avenues will generate income and employment for all states and also enable least cost electricity for consumers in the country.

### Reduction in GHG emissions during the transition

**Fig. 8:**
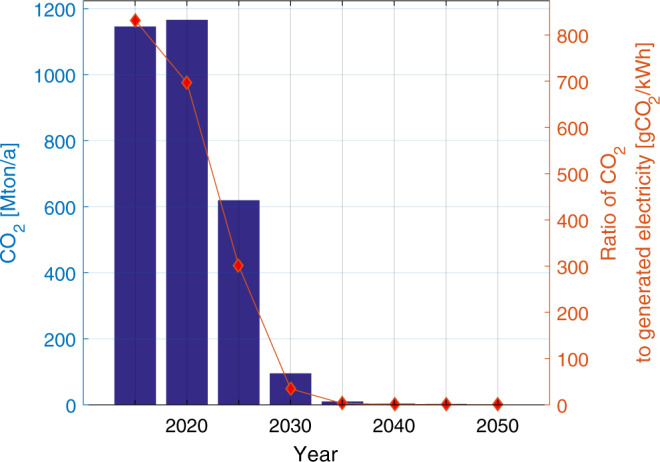
GHG emissions from the power sector during the energy transition from 2020 to 2050 across India. Deep defossilisation of the power sector is possible by 2030 and a steady decline of emissions is possible beyond 2030 up to 2050.

The reduction in GHG emissions as a function of increasing shares of renewables during the transition is shown in Fig. 8. The results indicate a rapid decline in GHG emissions in the power sector, reaching almost zero well before 2050 (2040) in comparison to current levels of about 1200 MtCO_2eq_/a in 2020, on a national level. This reduction in GHG emissions is in line with the Paris Agreement target of limiting temperature rise to 1.5°C above pre-industrial levels by 2050, with zero GHG emissions across all energy sectors. As the power sector drives the transition across other energy sectors (heat, transport and industry) with increased electrification, which is a growing trend even in India, particularly with increased impetus on electric vehicles, a rapid transition will be a fundamental enabler of a climate compliant energy pathway for India.

Due to large share of coal in its electricity generation, the CO_2eq_ intensity of electricity generation in India is one of the highest in the world. During the transition, CO_2eq_ intensity rapidly declines as coal is replaced by renewables, which indicates a deep defossilisation by 2030. The level of air pollution is also expected to decline throughout India during the transition to near zero by 2040, therefore reducing associated health impacts, which has both societal as well as economic benefits.

### Challenges and Uncertainties

In this research, we show a best policy scenario for the transition of the Indian power sector. Within the scope of this study, the available renewable resources in each state are adequate to satisfy the growing power demand in each state. Brown et al.^55^ clearly respond to major barriers and concerns that are often associated with 100% renewables-based power systems. Nevertheless, challenges and uncertainties do exist in the modelling of future power systems.

- The primary challenges are the stability of the power system with comparatively low inertia and the inability of the power system to balance short-term variability between generation and demand. However, lack of inertia from rotating masses in a 100% renewables-based power system can be mitigated by integration of synthetic inertia and improved algorithms for power inverters for generation and batteries^47,56^, as described by Oyewo et al.^57^ for a 100% renewables-based power system for sub-Saharan Africa.
- The cost developments of the different renewable energy technologies considered in the study will be uncertain due to various factors, such as the recent price hike in silicon due to COVID-19 related value chain distortions. While the costs of solar PV and batteries have fallen rapidly by almost 70–80% in the last decade, this trend is expected to continue during the transition period, based on the historic learning rates of the renewable energy technologies.
- The criticality of certain raw materials like silver, copper, aluminium and lithium is seen as a potentially limiting factor in the fast growth of renewable energy and storage technologies. However, solutions do exist, and a growth in the circular economy would reduce primary production.
- Social acceptance of technologies and political will are the most uncertain aspects of the transition. These aspects change overtime and are hard to integrate into techno-economic analysis. However, qualitative assessments can be made, and we assume that society and government policies will follow a low-cost, sustainable, and a climate compliant pathway.

### Renewables – Key enabler of the power sector transition

In this study, a best policy scenario was devised to analyse an energy transition pathway towards integrating 100% renewable energy by 2050 for the various states in India. It is acknowledged that challenges and uncertainties do exist in such a transition.

Despite the challenges and uncertainties, the findings of this study, based on the financial and technical assumption used, show that a cost optimal rapid transition away from coal and towards 100% renewable energy based electricity generation across the different states of India can be achieved by integrating large shares of solar PV, batteries, wind energy and supported by a strong transmission and distribution infrastructure. This transition not only decreases the cost of electricity generation by phasing out fossil fuels but also enables a rapid decrease in CO_2_ emissions and losses in the power sector. Future analysis could capture various uncertainties associated with such a transition pathway.

The total installed capacity of solar PV reaches 3000 GW by 2050, contributing almost 73% to the total power generation of India. On the other hand, wind plays a supporting role, which complements perfectly during the monsoon season. The contribution of wind energy to total power generation reaches 19% in 2050. Solar PV and wind energy are already low-cost in India. With the prices of batteries continuously decreasing^58^, a rapid transition of the power sector towards utilising 100% renewables is a possibility. Additionally, utilising the huge resource potential of solar and wind energy should be the preferred strategy that India should focus on. This will not only solve the increasing toxic air pollution and water stress issues^59^, but also reduce the growing fossil fuel import bill that has been over 100 bUSD for the last few years^60^. Additionally, this transition could decrease the system’s energy transformation losses to as low as 9.3% in 2050, from a high of 57% in 2020 (refer to Supplementary Information Figs. 11 and 12).

As the share of renewables increases across the different states, storage technologies, especially batteries and the transmission grid provide much needed flexibility during the transition, without increasing the total cost of the system. The system’s LCOE decreases from 71 €/MWh in 2020 to 38 €/MWh by 2050. The CO_2_ emissions cost enables a faster transition. However, without such a cost, the LCOE still decreases by 30%, compared to the 2020 levels. Additionally, if the cost structure of 2020 is frozen to satisfy the power demand of 2050 (a non-transition scenario), this would lead to an LCOE of 268 €/MWh, which is almost six times higher than a 100% renewable energy based power system. This shows that a rapid transition of the power system across the states in India is not only based on the direct cost competitiveness of renewables but also on indirect economic benefits, such as reducing air pollution and corresponding health costs and creating additional jobs. Finally, pathways showing fully renewable power systems fulfil wide ranging environmental, socio-economic, and ethical sustainability criteria in a comprehensive manner. Therefore, fully renewable energy system scenarios should be regarded as real policy options and set as a reference for alternative pathways.

### Policy implications - Opportunity for India to be a trendsetter

The growth in electricity use, among all energy carriers, is the fastest, confirming the role of electricity as the backbone of the current as well as future energy systems, globally as well as in India. This was amplified further due to the disruptions caused by the COVID-19 pandemic. Electricity as an energy vector kept societies functioning without major disruptions^61^. Also, the growing trend of ‘electrification’ of energy sectors reiterates the importance of renewable energy based electricity as an enabler of a sustainable and low-cost energy transition. This increasing trend of electrification obligates India to develop a low-cost resilient future power system, decoupling it from external price shocks of imported fossil fuels and increasing its energy security.

The power sector in India has undergone a massive transformation during the last decade. Government led reforms such as establishing a single national power grid by connecting regional grids, expanding electricity access to all households, and a massive increase in renewable energy installations have created momentum for increased electricity use and a clean energy transition^62^. A recent example can be seen from the growth in VRE installations in Karnataka. Favourable state government policies for renewable project developers, involving local farmers and reducing dependence on coal imports, resulted in a conducive atmosphere for VRE development on a large scale^63,64^.

India will see the largest increase in energy demand in the next couple of decades, as a result of its expanding economy, population, urbanisation and industrialisation^65^. There is huge potential for India to leapfrog polluting technologies and satisfy the growing energy demand with renewable energy and storage technologies. Doing so without increasing CO_2_ emissions.

Currently, India does not directly implement a tax on carbon or GHG emissions. However, it does implement implicitly a form of taxation known as ‘fuel excise tax’. In 2021, this was 14.4 €/tCO_2_^66^. However, this is lower compared to the GHG emissions cost considered in this study, which is based on a proactive climate perspective. In this context, India could consider some additional taxes on emissions or similar mechanisms to internalise the adverse effects of fossil fuels. Additionally, the revenue collected could be used towards the development of renewable energy and sustainable technologies.

Already, India has one of the most ambitious renewable energy capacity expansion targets by 2030^12^. Some of the renewable energy rich states have renewable energy penetration levels larger than some of the developed countries^17^. However, even faster growth and steeper targets will be needed in the next few decades to stop the ill effects of climate change^5^. The average annual growth rate of renewables in India has been around 15%, while solar PV installations have grown by 26% annually since 2018. However, more needs to be done in the case of India to achieve its targets of renewable capacity installation. On the other hand, globally, renewable capacity installation grew by 45% in 2020^67^. China installed 136 GW of renewables in 2020, about 50 GW of solar PV and 73 GW of wind energy. The growth of renewables in Vietnam has been phenomenal, especially solar PV, growing by almost 1000%, with 11.7 GW of solar PV installed in 2020. Similarly, Australia had an annual growth in solar PV capacity of 35% in 2020, while per capita installed capacity of renewables was more than 250 W/person/year in 2020^68^.

This study shows that a faster and a cost optimal transition is possible with solar PV, wind energy and batteries. An ambitious long term target will give a clear message to investors and stakeholders that investing in fossil fuel based electricity generating technologies will result in stranded assets.

About 137 countries have already announced their net zero targets^69^. Among top carbon emitting countries, the US and the European Union have set a target of carbon neutrality by 2050, while China has set a target of 2060^69^. India, as the third largest GHG emitter, announced their net zero emissions target by 2070 at the recently concluded COP26. The results of this study show that a rapid transition pathway for achieving net zero emissions in the power sector can accommodate India’s development imperatives of energy affordability, accessibility and mitigating air pollution in its cities, while maintaining robust economic growth.

Every country will have a different pathway towards net zero emissions, more so for India due to its uniqueness. However, one thing is clear: electricity will be the backbone of the entire energy system, with solar PV and batteries emerging as the most dominant technologies in the transition. The COVID-19 pandemic has shown us that electricity kept societies functioning when everything else stopped.

## Methods

### LUT energy system transition model

The LUT Energy System Transition Model is developed to assess various possible techno-economic energy transition pathways on global, national, and regional levels. The model has been previously used to study the transition of the global^46,47^, regional^70–72^ and national^45,73,74^ power and energy systems. The specific characteristics of individual countries or regions are captured with corresponding model input parameters and assumptions.

The primary objective of this study is to define a least-cost power system incorporating renewables for all the specified years during the transition across the different states of India, using specific initial assumptions for key technologies. The transition from the current coal dominated to a fully renewable energy-based power system by 2050 is not only cost competitive but also rapidly reduces GHG emissions. This pathway provides an alternative scenario of affordability, sustainability, and emissions reduction, mainly utilising solar, wind and batteries, further complemented by hydropower.

To evaluate an energy transition pathway from 2015 to 2050, the LUT Energy System Transition modelling tool^47,75^ is applied to the power sector across the states of India. A hierarchical modelling approach has been applied to reduce the complexity and allow simulation at high regional resolution in India. This method is described in Bogdanov et al.^76^. The model linearly optimises a set of given constraints on an hourly resolution for an entire year (further details of the model along with the respective mathematical representation of the target functions and constraints can be found in the next section). According to Prina et al.^77^, the LUT model is one of the most sophisticated among all the investigated long term energy system models.

Two important constraints are applied to the model. First, no new power capacity installed after 2015 for coal, nuclear and conventional fossil oil-based power plants; the exception here being capacities commissioned and grid connected between 2015 and 2019, as mentioned briefly earlier. Second, in a specific year, growth in the share of installed capacity of renewable energy technologies cannot exceed more than 4% of the total installed capacity per annum from 2020 onwards. Additional information on the constraints can be found in the next section.

The model defines a cost-optimal capacity mix of generation, storage, transmission, and flexibility technologies to match the hourly power demand for each of the 22 states for a reference year. The costs of operating a power system for an entire year are calculated as a sum of the annualised capital expenditures (Capex), the Weighted Average Cost of Capital (WACC), Operational Fixed (Opex fixed) and Operational Variable (Opex var) expenditures, ramping costs for thermal generators, fuel costs and the cost of GHG emissions for all available technologies. The detailed financial and technical assumptions for all technologies are given in the Supplementary Information Table 1−4.

In addition to the energy system transition modelling, the power sector incorporates distributed self-generation and consumption of residential, commercial, and industrial PV prosumers. A prosumer is an individual entity generating their own electricity by installing rooftop solar PV and optional batteries and can also consume electricity from the grid (and supply excess generated electricity to the grid if regional policy allows). These prosumers are optimised exogenously with a different model describing rooftop PV capacities and battery development^78^. The prosumer modelling determines the cost-optimal solar PV capacities installed on rooftops with the battery energy storage, individually for residential (all roofs used for residential purposes such as residential houses, apartments, individual houses, etc.), commercial (all roofs used for commercial purposes such as commercial buildings, malls and government buildings) and industrial prosumers (all rooftop available from the industrial complexes).

The hourly profiles for solar PV consumption, battery charging and discharging, electricity supply from the grid, and feed-in of excess electricity to the grid are determined through the target function of minimisation of annual electricity costs. The details of the target function used for prosumers is given in the next section. The resulting output from the prosumer model defines the demand of the centralised power system. As a result of the integration of prosumers into the larger energy system, prosumers reduce the daily peak demand and, in turn, reduce the centralised system’s power plant capacities. Integration of large scale prosumers will require bidirectional smart meters, and it is assumed to be part of the prosumer setup.

The capacity built, electricity generated, storage and grid deployed are all based on the results of the applied target function and constraints. It is acknowledged that there could be various pathways to achieve a zero GHG emission power system by 2050, such as integrating large shares of nuclear energy, carbon capture and storage and biomass. However, in this study, a least cost scenario is highlighted by utilising abundant potential of solar and wind energy^79^.

The power sector transition modelling for India is performed by using the LUT Energy System Transition Model tool^47,75^. Under the assumption of perfect foresight of renewable energy power generation and power demand, the power system is linearly optimised on an hourly resolution for an entire year under a set of applied constraints. The optimisation is performed using a third-party solver. In this study, MOSEK ver.8 is used as an optimiser, but other solvers (Gurobi, CPLEX, etc.) can also be used. The model is compiled in the Matlab environment in LP file format so that the model can be read by most of the available solvers. After simulation, the results are parsed back into the Matlab data structure and post-processed.

**Fig. 9:**
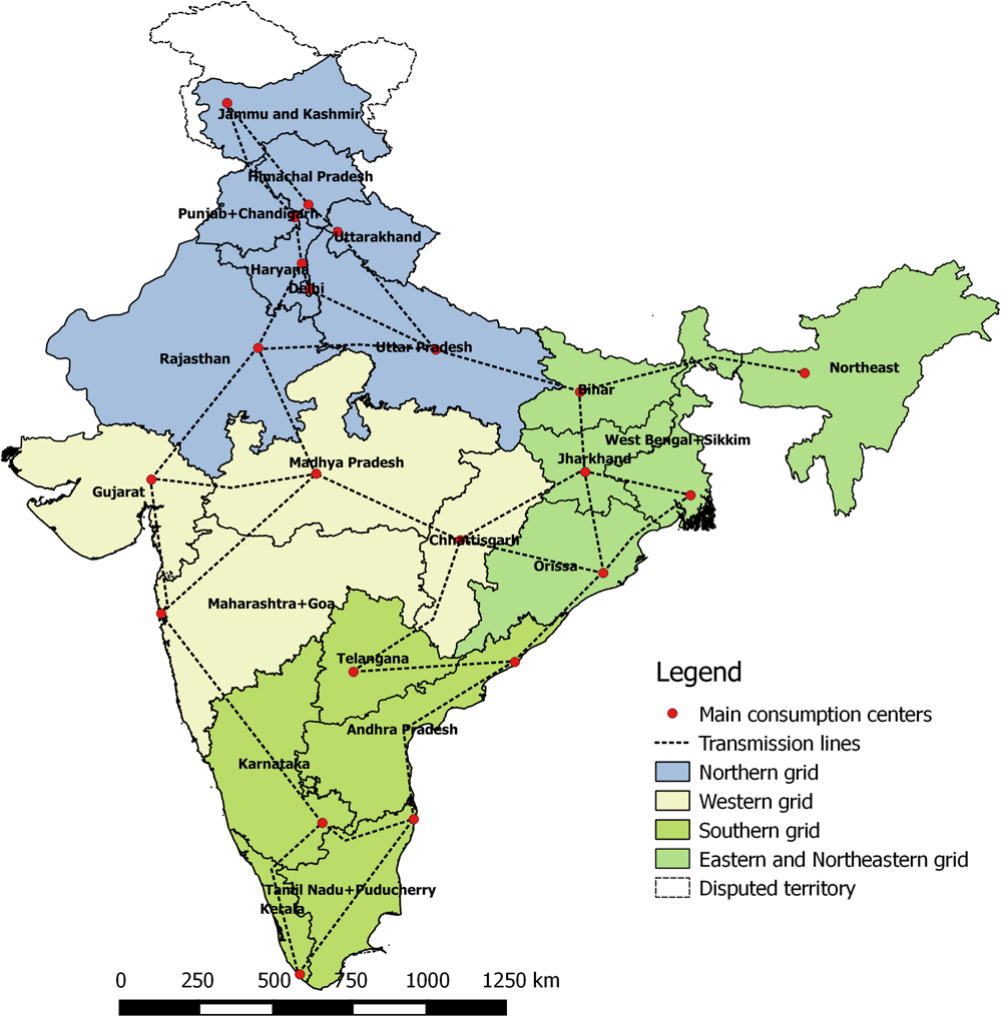
The four major regions constituted by the corresponding states/regions. India has five regional grids. In this analysis, we have combined the Eastern and Northeastern grids to form an Eastern grid, so we have four regional grids. All the major states are considered as shown, while smaller states and union territories are combined to the nearest state, except Delhi. The individual states within each of the regional grids are interconnected, and the regional grids are interconnected with each other. These transmission lines enable imports and exports between the states. It is assumed that the existing network of alternating current (AC) lines within the individual states will provide electricity to all end consumers.

A multi-node approach used in this study enables the description of any desired configuration of states and power transmission interconnections. To decrease the simulation time, a hierarchical modelling approach has been applied^76^. The modelling is performed in two steps. First, modelling of the system in a reduced regional resolution (4 regional grids). Second, modelling of each of the regional grids in full state resolution, considering the power flows between the regional grids simulated in the first step. The results represent the operations of the integrated power system in full resolution, where power can flow between all the states. Figure 9 describes the detailed regional configuration.

The main constraints for the optimisation are the matching of all types of generation and power demand for every hour of the applied year, and the optimisation criteria is to have a least annual cost of the power system. The hourly resolution of the model significantly increases the computation time; however, it guarantees that for every hour of the year, the total supply within a region covers the local demand and enables a more precise system description, including synergy effects of different system components.

### Target function

The target of the system optimisation is to minimise the total annual cost of an integrated power system, calculated as the sum of the annual costs of installed capacities of different technologies, the costs of power generation and ramping technologies. This target function includes the annual costs of the power sector. The target function of the applied energy model for minimising annual costs is presented in Eq. (1) using the abbreviations: states/regions (*r*, reg), generation, storage and transmission technologies (*t*, tech), capital expenditures for technology *t* in region *r* (CAPEX_*r,t*_), capital recovery factor for technology *t* in region *r* (crf_*r,t*_), fixed operational expenditures for technology *t* in region *r* (OPEXfix_*r,t*_), variable operational expenditures technology *t* in region *r* (OPEXvar_*r,t*_), installed capacity in the region *r* of technology *t* (instCap_*r,t*_), annual generation by technology *t* in region *r* (E_gen*,t,r*_), cost of ramping of technology *t* (rampCost_*t*_) and sum of power ramping values during the year for the technology *t* in the region *r* (totRamp_*r,t*_).

1 $$\min \left(\mathop{\sum }\limits_{r=1}^{{{{{{\rm{reg}}}}}}}\mathop{\sum }\limits_{t=1}^{{{{{{\rm{tech}}}}}}}\left({{{{{{{\rm{CAPEX}}}}}}}_{r,t}\cdot {crf}}_{r,t}+{{OPEXfix}}_{r,t}\right)\cdot {{instCap}}_{r,t}+ {{OPEX}{var}}_{r,t}\cdot {E}_{{gen},r,t}+{{rampCost}}_{t}\cdot {{totRamp}}_{r,t}\right)$$

The target function only considers the cost assumptions for the given step of transition as the previously built capacity is defined as a lower limit for the total capacity (instCap_*t,r*_), and thus the previously built capacity costs do not affect the optimisation.

The rooftop prosumer system (solar PV and batteries) is realised in an independent sub model with a slightly different target function. The prosumer system is optimised for each region and each power demand segment (residential, commercial and industrial) independently, even if the states or regions are interconnected with each other. The target function includes annual costs of the prosumers power generation and storage and the cost of electricity bought from the distribution grid. The cost of electricity sold to the distribution grid is deducted from the total annual cost. The target function of the applied prosumer model for minimising annual costs is presented in Eq. (2) using the abbreviations: generation and storage technologies (*t, tech*), capital expenditures for technology *t* (CAPEX_*t*_), capital recovery factor for technology *t* (crf_*t*_), fixed operational expenditures for technology *t* (OPEXfix_*t*_), variable operational expenditures for technology *t* (OPEXvar_*t*_), installed capacity of technology *t* (instCap_*t*_), annual generation by technology *t* (*E*_gen*,t*_), retail price of electricity (elCost), feed-in price of electricity (elFeedIn), annual amount of electricity bought from the grid (*E*_grid_), annual amount of electricity sold from the grid (*E*_curt_).

2 $$\min \bigg(\mathop{\sum }\limits_{t=1}^{{tech}}\left({{{CAPEX}}_{t}\cdot {crf}}_{t}+{{OPEXfix}}_{t}\right)\cdot {{instCap}}_{t}+{{OPEX}{var}}_{t}\\ \cdot {E}_{{gen},t}+\,{elCost}\cdot {E}_{{grid}}+{elFeedIn}\cdot {E}_{{curt}}\bigg)$$

### Energy balance constraints

The main constraint for optimising the power sector is matching power generation and demand for every hour of the applied year. For every hour of the year, the total generation within a region and electricity imported should cover the local electricity demand.

3 $$\forall {{{{{\rm{h}}}}}}\in	 \left[1,8760\right]\,\mathop{\sum }\limits_{t}^{{tec}h}{E}_{{gen},t}+\mathop{\sum }\limits_{r}^{{reg}}{E}_{{imp},r}+\mathop{\sum }\limits_{t}^{{stor}}{E}_{{stor},{disc}h} \\ 	={E}_{{demand}}+\mathop{\sum }\limits_{r}^{{reg}}{E}_{{\exp },r}+\mathop{\sum }\limits_{t}^{{stor}}{E}_{{stor},ch}+{E}_{{curt}}$$

Equation (3) describes constraints for the energy flows of a region. Abbreviations: hours (*h*), technology (*t*), all modelled power generation technologies (tech), sub-region (*r*), all sub-regions (reg), electricity generation (*E*_gen_), electricity import (*E*_imp_), storage technologies (stor), electricity from discharging storage (*E*_stor,disch_), electricity demand (*E*_demand_), electricity exported (*E*_exp_), electricity for charging storage (*E*_stor,ch_), curtailed excess energy (*E*_curt_). The energy loss in the high voltage direct current (HVDC) and alternating current (HVAC) transmission grids and energy storage technologies are considered in storage discharge and grid import value calculations.

Apart from this, various financial and technical assumptions that are utilised for the cost optimisation of the model are presented in the Supplementary Information Table 1−4.

The important constraints applied in the modelling are given below:

1. No new power capacity will be installed after 2015 for coal, nuclear and conventional fossil oil-based power plants, mainly due to their inability to fulfill the high sustainability criteria set in the model. The capacities commissioned and grid connected between 2015 and 2019 are an exception. It is assumed that coal and oil-fired power plants under construction and planned capacities are scrapped and not commissioned. All fossil fuel-based power plant capacities are fully amortised until the end of their technical lifetimes to facilitate a gradual phase out. Their utilisation is cost optimised so that, in later periods for some states, full load hours or capacity factors even decline to zero, due to their higher per unit cost of electricity production. Even though these capacities do not produce electricity, they have to be amortised for political reasons, a procedure which is known as cold reserve (also called security reserve). Gas turbines and multi-fuel ICE are permitted to be installed beyond 2015 due to lower carbon emissions and the possibility to accommodate renewable electricity based methane (e-methane), bio-methane and even green hydrogen into the system. Gas-fired power plants are more flexible, not only in their ramping rates but also in utilising different e-fuels
2. In a specific year, growth in the shares of installed capacities of renewable energy technologies cannot exceed more than 4% per annum from 2020 onwards in congruence with empirical data^80^

The active capacity existing in the system is defined on each of the steps for each of the regions, based on the data of the capacity installed at previous steps and the lifetime for a given technology at given commissioning year as presented in Eq. (4) using the abbreviations: years (*y, year*), generation and storage technologies (*t, tech*), existing active capacity for technology *t* at modelled year (existingCap_*t,year*_), new built capacity for technology *t* at previous year *y* (newCap_*t,y*_), lifetime of the capacity of technology *t* built in year *y* (N_*t,y*_):

4 $$\forall t\in \left[{tech}\right]\,{{{{{{\rm{existingCap}}}}}}}_{t,{year}}=\mathop{\sum }\limits_{y=1960}^{{year}}{{{{{{\rm{newCap}}}}}}}_{t,y}\cdot \left((y+{N}_{t,y}) \, > \, {year}\right)$$

Then the model optimisation results in the optimal regional capacity of the technologies in the given year, which defines the new built capacity needed by the system as defined in Eq. (5) using the abbreviations: modelling year (*year*), generation and storage technologies (*t, tech*), new built capacity for technology *t* at a given year *year* (newCap_*t,year*_), total capacity for technology *t* at a given year *year* as defined by the model optimisation (${instCap}$_*t,year*_), existing active capacity for technology *t* at modelled year (existingCa*p*_*t,year*_):

5 $$\forall t\in \left[{tech}\right]\,{{{{{{\rm{newCap}}}}}}}_{t,{year}}={{{{{{\rm{instCap}}}}}}}_{t,{year}}-{{{{{{\rm{existingCap}}}}}}}_{t,{year}}$$

The energy cost calculations in the post-processing phase are based in a hierarchical approach, where the annualised cost of the system considers the financial assumptions in the periods when these capacities were built, unlike the approach used in the optimisation and described in Eq. (1). For the variable opex calculations, the energy output of technologies is split accordingly to the capacity age structure as defined in Eq. (6) using the abbreviations: modelling year (*year*), all years from 1960 (*y*), generation and storage technologies (*t, tech*), annual generation by technology *t* by capacity built at year *y* (*E*_genSplit*,t,y*_), new built capacity for technology *t* built at year *y* (newCap_*t,y*_), annual generation by technology *t* defined by the model for the modelling year *year* (*E*_gen,*t,year*_), total capacity for technology *t* at given a year *year* as defined by the model optimisation (instCap_t*,year*_) lifetime of the capacity of technology *t* built at year *y* (*N*_*t,y*_):

6 $$\forall t 	 \in \left[{tech}\right],\forall {{{{{\rm{y}}}}}}\in \left[1960\ldots {year}\right]\,{E}_{{{{{{\rm{genSplit}}}}}},t,y} \\ 	={E}_{{{{{{\rm{gen}}}}}},t,{year}}\cdot \left({{{{{{\rm{newCap}}}}}}}_{t,y}\cdot \left(\left(y+{N}_{t,y}\right) > {year}\right)\right)/{{{{{{\rm{instCap}}}}}}}_{t,{year}}$$

The annnualised cost of the system at a given year is calculated accordingly to the Eq. (7) using the abbreviations: modelling year (*year*), all years from 1960 (*y*), generation and storage technologies (*t, tech*), capital expenditures for technology *t* in region *r* and year *y* (CAPEX_*r,t,y*_), capital recovery factor for technology *t* in region *r* and year *y* (crf_*r,t,y*_), fixed operational expenditures for technology *t* in region *r* and year *y* (OPEXfix_*r,t,y*_), variable operational expenditures technology *t* in region *r* and year *y* (OPEXvar_*r,t,y*_), new built capacity for technology *t* built in region *r* at year *y* (newCap_*r,t,y*_), lifetime of the capacity of technology *t* built at year *y* (*N*_*t,y*_), annual generation by technology *t* in region *r* in year *year* by capacity built at year *y* (*E*_genSplit*,r,t,y*_), cost of ramping of technology *t* (rampCost_*t*_) and sum of power ramping values during the year for the technology *t* in the region *r* (totRamp_*r,t*_):

7 $${{{{{{\rm{annualCost}}}}}}}_{{year}} 	=\,\mathop{\sum }\limits_{r=1}^{{reg}}\mathop{\sum }\limits_{t=1}^{{tech}}\mathop{\sum }\limits_{y=1960}^{{year}}\left({{{{{{{\rm{CAPEX}}}}}}}_{r,t,y}\cdot {{{{{\rm{crf}}}}}}}_{r,t,y}+{{{{{{\rm{OPEXfix}}}}}}}_{r,t,y}\right)\\ 	 \cdot \Big(\left({{{{{{\rm{newCap}}}}}}}_{r,t,y}\cdot \left(\left(y+{N}_{t,y}\right) > {year}\right)\right) \\ 	+ {{{{{{\rm{OPEX}}}}}}{{{{{\rm{var}}}}}}}_{r,t,y}\cdot {E}_{{{{{{\rm{genSplit}}}}}},r,t,y}+{{{{{{\rm{rampCost}}}}}}}_{t}\cdot {{{{{{\rm{totRamp}}}}}}}_{r,t}$$

This historical cost calculation approach is used for other cost calculations including LCOE and split of LCOE in sub-categories.

**Fig. 10:**
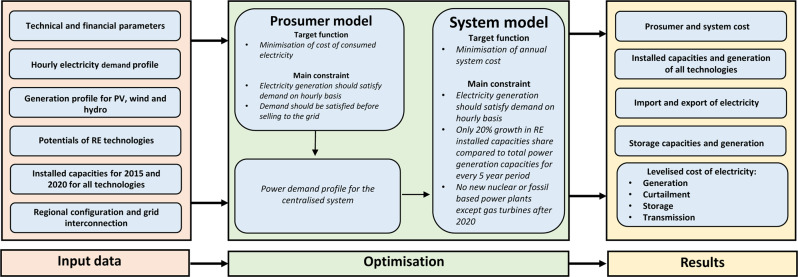
Schematics of the LUT Energy System Transition model. The model consists of various primary data as an input to the optimisation process, where, first, the prosumer target function is optimised, and in the second step, the system target function. Different optimised results are obtained as an output.

The schematic of the LUT Energy System Transition Model with the various inputs, optimisation and results is illustrated in Fig. 10.

### Development of electricity demand

The average per capita electricity demand is assumed to rise from 1.2 MWh in 2020 to 3.5 MWh in 2050, while the population is projected to increase to 1.7 billion by 2050, as highlighted in the Supplementary Information Fig. 1. Total electricity demand of the Indian power sector is estimated to increase to about 5921 TWh by 2050, which represents a compound average annual growth rate of around 4.9% in the energy transition period, in line with the expectations of the government and other energy institutions^81^. Use of electricity in other energy sectors (such as heat, transport and industry) is not considered in this research, which could lead to an additional increase in electricity demand during the transition period. The synthetic electricity demand profiles from 2015 until 2050 are generated for each of the states, based on the methods applied by Toktarova et al.^82^. Load profile will be different for the centralised power system due to partial load covering by prosumers. The seasonal and daily variations are captured in the load profiles up to 2050 across all the 22 states in the country.

### Electricity generation technologies and other resources

The model is integrated with all crucial aspects of power systems: generation, storage and transmission^83^.

- **Technologies for electricity generation**: Solar PV fixed tilted, solar PV single-axis north-south tracking, solar PV rooftop, concentrating solar thermal power (CSP), wind onshore and offshore, hydropower run-of-river, hydropower reservoirs, geothermal, bioenergy (solid biomass, biogas, and waste-to-energy). The existing fossil fuels-based generation technologies considered are coal and conventional oil based power plants, open cycle gas turbines (OCGT), combined cycle gas turbines (CCGT) and nuclear technologies. In addition, new technologies like multi-fuel reciprocating ICE (gas) and heavy-duty open cycle gas turbines (OCGT HD) make up the electricity generation technologies.
- **Energy storage technologies**: Lithium-ion (Li-ion) batteries and pumped hydro energy storage (PHES) for short-term storage. Adiabatic compressed air energy storage (A-CAES) and thermal energy storage (TES) for medium-term storage. Gas storage including power-to-gas technology, which allows production of e-methane for the energy system for seasonal storage requirement.
- **Electricity transmission technologies**: The existing power grid, its future development, and impact on overall electricity transmission and distribution losses^84^ is taken into account in the transition. The states are interconnected with high voltage direct current (HVDC) or high voltage alternating current (HVAC) power lines. These transmission lines provide the required flexibility by spatial distribution of renewable-based electricity, especially in the monsoon season^48^, while reducing overall national system costs.

### Best Policy Scenario

The LUT Power System Transition Model can be utilised to generate wide-ranging power sector scenarios across the different regions of the world on a global-local scale. However, the objective of this study is to highlight a power sector scenario for the states in India interconnected via transmission lines in the context of achieving the goals of the Paris Agreement by reaching zero GHG emissions from the power sector in a technically feasible and economically viable manner. Therefore, a Best Policy Scenario is envisioned for the power sector from 2015 towards a cost-optimal power system by 2050. The results are visualised and presented in 5-year intervals through the transition from 2015-2050 for the power system transition across the states of India.

### Technical and financial assumptions

The key technical and financial assumptions, with the corresponding references, are presented in Table 1. A comprehensive list of all the assumptions used in this study is presented in Supplementary Information Table 1−4. The key assumptions are mostly taken from the Central Electricity Authority (CEA), and the Central Electricity Regulatory Commission (CERC). Table 2 presents the ramping costs for key power generation technologies. However, not all assumptions were available from these sources, therefore global assumptions were used in such cases. Each of these technical and financial assumptions are considered for 5-year time periods between 2015 to 2050. The average solar PV costs in India, in 2020 was 455 USD/kW i.e. ~424 €/kW^85^. The average lifetime is given in the range of 25-40 years in the NREL study^86^. Warranties are often used as an indicator of the economic lifetime of solar PV modules which is 25 years, while the modules can produce more than 80% of the original power after 25 years and upto 50 years^87^. Based on various project developers, and other stakeholders the useful life assumptions increased from an average of ~21.5 years in 2007 to ~32.5 years in 2019^86^. Currently, the assumptions range from 25 years to more than 35 years^86^. Increase in lifetime is expected as we go through the transition towards 2050, as observed from 2007 to 2019. For residential batteries, average cost of battery packs in India is 215 €/kWh^88^. The weighted average cost of capital (WACC) was set to 11% in 2015, declining steadily to 7% in 2050 (Table 3). However, in the case of residential solar PV prosumers, WACC is set to 4% due to lower financial return expectations. Electricity prices for residential, commercial and industrial consumers were taken from the Tariff Order for individual states, and extended to 2050 based on the methods of Breyer and Gerlach^89^. The excess electricity generated by PV prosumers is fed into the national grid and is assumed to be incentivised for a transfer price of 0.02 €/kWh. The model ensures that prosumers satisfy their own demand for electricity before feeding it to the grid. The costs for biomass are calculated using data from the IEA^90^ and IPCC^91^. Solid wastes gate fees are 50 €/ton in 2015, 53 €/ton in 2020, 59 €/ton in 2025, 68 €/ton in 2030, 80 €/ton in 2035, 95 €/ton in 2040, 100 €/ton in 2045 and 2050; the assumption is based that gate fees will gradually increase globally and by 2050 reach 100 €/ton as in most of the developed countries. It is assumed that the GHG emissions cost increases from 28 €/tCO_2_ in 2020 to 150 €/tCO_2_ in 2050^92^.

**Table 1:** Technical and financial assumptions for key power system technologies used in the Indian energy transition from 2015 to 2050

Technology		Unit	2015/2017	2020	2025	2030	2035	2040	2045	2050	Ref
PV rooftop - residential	Capex	€/kW_el_	1360	1045	842	715	622	551	496	453	^98^
	Opex fix	€/(kW_el_ a)	20.4	9.1	7.7	6.7	5.9	5.3	4.8	4.4	
	Opex var	€/(kWh_el_)	0	0	0	0	0	0	0	0	
	Lifetime	years	30	30	35	35	35	40	40	40	
PV rooftop - commercial	Capex	€/kW_el_	1360	689	544	456	393	345	308	280	^98^
	Opex fix	€/(kW_el_ a)	20.4	9.1	7.7	6.7	5.9	5.3	4.8	4.4	
	Opex var	€/(kWh_el_)	0	0	0	0	0	0	0	0	
	Lifetime	years	30	30	35	35	35	40	40	40	
PV rooftop - industrial	Capex	€/kW_el_	1360	512	397	329	281	245	217	197	^98^
	Opex fix	€/(kW_el_ a)	20.4	9.1	7.7	6.7	5.9	5.3	4.8	4.4	
	Opex var	€/(kWh_el_)	0	0	0	0	0	0	0	0	
	Lifetime	years	30	30	35	35	35	40	40	40	
PV optimally tilted	Capex	€/kW_el_	733	432	336	278	237	207	184	166	^99–101^
	Opex fix	€/(kW_el_ a)	9.3	7.8	6.5	5.7	5.0	4.5	4.0	3.7	
	Opex var	€/(kWh_el_)	0	0	0	0	0	0	0	0	
	Lifetime	years	30	30	35	35	35	40	40	40	
PV single-axis tracking	Capex	€/kW_el_	1150	475	370	306	261	228	202	183	^100,102^
	Opex fix	€/(kW_el_ a)	17.3	9.0	7.0	6.0	6.0	5.0	4.0	4.0	
	Opex var	€/(kWh_el_)	0	0	0	0	0	0	0	0	
	Lifetime	years	30	30	35	35	35	40	40	40	
Wind onshore	Capex	€/kW_el_	800.0	800	783.3	767.0	749.0	749.0	749.0	749.0	^99,100^
	Opex fix	€/(kW_el_ a)	15.0	15.0	13	11	8.0	8.0	8.0	8.0	
	Opex var	€/(kWh_el_)	0	0	0	0	0	0	0	0	
	Lifetime	years	25	25	25	25	25	25	25	25	
Hydro Reservoir/ Dam	Capex	€/kW_el_	1650	1650	1650	1650	1650	1650	1650	1650	^103^
	Opex fix	€/(kW_el_ a)	49.5	49.5	49.5	49.5	49.5	49.5	49.5	49.5	
	Opex var	€/(kWh_el_)	0.003	0.003	0.003	0.003	0.003	0.003	0.003	0.003	
	Lifetime	years	50	50	50	50	50	50	50	50	
Hydro Run-of-River	Capex	€/kW_el_	2560	2560	2560	2560	2560	2560	2560	2560	^103^
	Opex fix	€/(kW_el_ a)	76.8	76.8	76.8	76.8	76.8	76.8	76.8	76.8	
	Opex var	€/(kWh_el_)	0.005	0.005	0.005	0.005	0.005	0.005	0.005	0.005	
	Lifetime	years	50	50	50	50	50	50	50	50	
Coal Power Plant	Capex	€/(kW_el_)	867	934	1045	1156	1267	1378	1489	1600	^104^
	Opex fix	€/(kW_el_ a)	24.0	23.6	23.0	22.4	21.8	21.2	20.6	20.0	
	Opex var	€/(kWh)	0.001	0.001	0.001	0.001	0.001	0.001	0.001	0.001	
	Efficiency	%	32	42	42	42	43	43	43	43	
	Lifetime	years	40	40	40	40	40	40	40	40	
Nuclear Power Plant	Capex	€/(kW_el_)	4511	4571	4672	4773	4874	4974	5075	5175	^105–107^
	Opex fix	€/(kW_el_ a)	85.0	86.1	88.0	83.0	84.8	79.3	80.9	78.8	
	Opex var	€/(kWh_el_)	0.003	0.003	0.003	0.003	0.003	0.003	0.003	0.003	
	Efficiency	%	34	34	34	35	35	35	35	35	
	Lifetime	years	40	40	40	40	40	40	40	40	
CCGT	Capex	€/(kW_el_)	623	637	660	683	706	729	752	775	^104,108^
	Opex fix	€/(kW_el_ a)	23.44	23.1	22.5	21.9	21.3	20.7	20	19.375	
	Opex var	€/(kWh_el_)	0	0	0	0	0	0	0	0	
	Efficiency	%	52.2	52.2	52.2	52.2	53.1	54	54	54	
	Lifetime	years	35	35	35	35	35	35	35	35	
OCGT HD	Capex	€/(kW_el_)	450	445	440	435	430	425	420	415	^104,108^
	Opex fix	€/(kW_el_ a)	23.4	11.3	10.6	9.9	9.2	8.5	7.8	7.1	
	Opex var	€/(kWh_el_)	0	0	0	0	0	0	0	0	
	Efficiency	%	28	30	33	35	38	40	43	45	
	Lifetime	years	35	35	35	35	35	35	35	35	
Open cycle Aeroderivative	Capex	€/(kW_el_)	550	540	530	520	510	500	490	480	
	Opex fix	€/(kW_el_ a)	11.3	11.3	10.6	9.9	9.2	8.5	7.8	7.1	
	Opex var	€/(kWh_el_)	0	0	0	0	0	0	0	0	
	Efficiency	%	0.39	0.40	0.42	0.42	0.43	0.44	0.45	0.45	
	Lifetime	years	35	35	35	35	35	35	35	35	
RECIP oil based	Capex	€/(kW_el_)	385	385	385	385	385	385	385	385	^109^
	Opex fix	€/(kW_el_ a)	11.5	11.5	11.5	11.5	11.5	11.5	11.5	11.5	
	Opex var	€/(kWh_el_)	0	0	0	0	0	0	0	0	
	Efficiency	%	28	28	28	28	29	29	30	30	
	Lifetime	years	20	20	20	20	20	20	20	20	
RECIP Gas	Capex	€/(kW_el_)	578.5	569.0	553.0	537.0	522.0	506.0	491.0	475.0	
	Opex fix	€/(kW_el_ a)	15.3	15.3	14.6	13.9	13.2	12.5	11.8	11.1	
	Opex var	€/(kWh_el_)	0	0	0	0	0	0	0	0	
	Efficiency	%	0.47	0.48	0.48	0.49	0.49	0.50	0.50	0.51	
	Lifetime	years	30	30	30	30	30	30	30	30	
Biomass Power Plant	Capex	€/(kW_el_)	760.0	857	1019	1181	1343	1505	1668	1830	^99,100^
	Opex fix	€/(kW_el_ a)	53.3	51.5	48.4	45.3	42.2	39.1	36	32.9	
	Opex var	€/(kWh_el_)	0.004	0.004	0.004	0.004	0.004	0.004	0.004	0.004	
	Efficiency	%	35	36	37	37	38	38	39	39	
	Lifetime	years	25	25	25	25	25	25	25	25	
Battery storage	Capex	€/kWh_el_	400	270	182	134	108	92	78	70	^98^
	Opex fix	€/(kWh_el_ a)	24.0	9.0	5.0	3.8	3.0	2.5	2.1	1.9	
	Opex var	€/(kWh_el_)	0	0	0	0	0	0	0	0	
	Efficiency	%	90	91	92	93	94	95	95	95	
	Lifetime	years	15	20	20	20	20	20	20	20	
Pumped Hydro Energy Storage (PHES)	Capex	€/kWh_el_	89.0	89.0	89.0	89.0	89.0	89.0	89.0	89.0	
	Opex fix	€/(kWh_el_ a)	1	1	1	1	1	1	1	1	
	Opex var	€/(kWh_el_)	0	0	0	0	0	0	0	0	
	Efficiency	%	85	85	85	85	85	85	85	85	
	Self-discharge	%/h	0	0	0	0	0	0	0	0	
	Lifetime	years	50	50	50	50	50	50	50	50	

Selected technologies are listed. Further technologies can be found in the Supplementary Information Table 1.

**Table 2:** Ramping costs for key power generation technologies

Technology	Unit	
Coal PP	€/MW	54.3
Nuclear PP	€/MW	54.3
CCGT	€/MW	25.0
OCGT	€/MW	22.9
Biomass PP	€/MW	54.3

Data adopted from Deutsches Institut für Wirtschaftsforschung^110^. Selected technologies are listed. Further technologies can be found in the Supplementary Information Table 2.

**Table 3:** Financial assumptions for the fossil and nuclear fuel prices and GHG emission cost

Name of component	Unit	2015/2017	2020	2025	2030	2035	2040	2045	2050	Ref.
Coal	€/MWh_th_	9.9	9.9	10.8	11.8	13.1	14.3	14.3	14.3	^111^
Fuel oil	€/MWh_th_	101.1	101.1	114.3	127.5	126.0	124.9	124.9	124.9	^106,112^
Fossil gas	€/MWh_th_	36.1	36.1	48.8	53.2	58.8	65.4	65.4	65.4	^112,113^
Uranium	€/MWh_th_	2.6	2.6	2.6	2.6	2.6	2.6	2.6	2.6	^107^
GHG emissions	€/tCO_2eq_	9	28	52	61	68	75	100	150	^92^
WACC		11.0 %	11.0 %	9.7 %	8.5 %	7.0 %	7.0 %	7.0 %	7.0%	^114^
**GHG emissions by fuel type t**_**CO2eq**_**/MWh**_**th**_										
**Coal**^115^			**Oil**^115^				**Fossil gas**^116^			
0.34			0.25				0.21			

The referenced values are till 2040 and are kept stable for later periods (fuels).

### Capacity factor profiles

The hourly feed-in profiles for solar PV, wind energy and hydropower were provided as an input to the model. The dataset used for solar irradiation and wind speed is in a 0.45° X 0.45° spatial resolution for the real weather conditions. The feed-in full load hours (FLH)/capacity factors for the individual states are computed on the basis of the 0.45° X 0.45° spatially resolved single sub-area data using a weighted average formula. The individual state capacity factors are calculated using the following rule: 0–10% best sub-areas of a state are weighted by 0.3, 10–20% best sub-areas of a state are weighted by 0.3, 20– 30% best state of a region are weighted by 0.2, 30–40% best sub-areas of a state are weighted by 0.1 and 40–50% best sub-areas of a state are weighted by 0.1. The FLH/capacity factor of solar PV and wind energy estimated at a high geospatial resolution across the country are given below in Supplementary Figs.2 and 3.

### Renewable energy potentials

The potential capacities, or the upper limits for solar PV and wind energy, are based on land use limitations and specific capacity densities. The area covered by solar PV plants is set at a maximum of 6% of the total land area available in each of the states. The average specific capacity density of solar PV is assumed to be 75 MW/km^2^ for the entire transition period. This is based on 15% module efficiency and a 50% ground coverage ratio^93^, and is confirmed by empiric data^94^. However, increase in the efficiency of the PV modules that would impact the specific capacity density is not considered. The total calculated installable potential for utility-scale solar PV in India is 14223 GW.

For onshore wind power plants, land use limitation is set to a maximum of 4%, while the average specific capacity density is assumed to be 8.4 MW/km^2,^^93^. The total calculated installable potential for onshore wind energy is 1062 GW.

For hydropower plants and pumped hydro energy storage (PHES), the potential was set to 150% and 200% of the already installed capacities of 2015. The geothermal energy potential was calculated according to the methods described in Aghahosseini and Breyer^95^. The biomass potentials were calculated based on the methodology described in Mensah et al.^96^.

## Supplementary information

Supplementary Information File

## Data Availability

The data and the main model code that support the findings of this study is available from the authors on reasonable request.
