# Supplementary material for: The role of renewables for rapid transitioning of the power sector across states in India
Source: Nat Commun. 2022 Sep 21;13:5499. doi: 10.1038/s41467-022-33048-8 (PMC9492668; doi:10.1038/s41467-022-33048-8)
Supplement: Supplementary file 1 — Supplementary Information File [file 41467_2022_33048_MOESM1_ESM.pdf]

Supplementary Information

The role of renewables for rapid transitioning of the power sector across states in India

Ashish Gulagi[1*], Manish Ram[1], Dmitrii Bogdanov[1], Sandeep Sarin[2], Theophilus Nii Odai Mensah[1], Christian Breyer[1*]

[1] LUT University, Yliopistonkatu 34, 53850 Lappeenranta, Finland

[2] Wärtsilä Corporation, Hiililaiturinkuja 2, 00180 Helsinki, Finland

*Corresponding authors

Technical and financial assumptions

The following tables show the various technical and financial assumptions used in the modelling of the power sector.

Supplementary Table 1: Technical and financial assumptions of energy system technologies used in the energy transition from 2015 to 2050. Core technologies are listed in Table 1 of the main manuscript file.

**Technology**		Unit	**2015/ 2017**	**2020**	**2025**	**2030**	**2035**	**2040**	**2045**	**2050**	**Ref**
Wind offshore	Capex	€/kW$_{el}$	2061	2003	1995	1979	1909	1896	1899	1897	
	Opex fix	€/(kW$_{el}$ a)	67.7	59.9	50.2	41.2	29.4	28.5	25.4	23.1	
	Opex var	€/(kWh$_{el}$)	0	0	0	0	0	0	0	0	
	Lifetime	years	20	25	25	25	25	25	25	25	
Geothermal power	Capex	€/kW$_{el}$	5250	4970	4720	4470	4245	4020	3815	3610	
	Opex fix	€/(kW$_{el}$ a)	80	80	80	80	80	80	80	80	[4,5]
	Opex var	€/(kWh$_{el}$)	0	0	0	0	0	0	0	0	
	Efficiency	%	23.9	23.9	23.9	23.9	23.9	23.9	23.9	23.9	
	Lifetime	years	40	40	40	40	40	40	40	40	
Steam turbine (CSP)	Capex	€/(kW$_{el}$)	760	740	720	700	670	640	615	600	
	Opex fix	€/(kW$_{el}$ a)	15.2	14.8	14.4	14	13.4	12.8	12.3	12	
	Opex var	€/(kWh$_{el}$)	0	0	0	0	0	0	0	0	
	Efficiency	%	37.2	38.3	40.3	43.0	43.0	43.0	43.0	43.0	
	Lifetime	years	25	25	25	25	30	30	30	30	
CHP Biogas	Capex	€/kW$_{el}$	1580	1463	1269	1074	880	685	491	296	
	Opex fix	€/(kW$_{el}$ a)	70.4	65.1	56.2	47.3	38.4	29.5	20.7	11.84	
	Opex var	€/(kWh$_{el}$)	0.001	0.001	0.001	0.001	0.001	0.001	0.001	0.001	[1,2]
	Efficiency elec.	%	33	34	37	40	42	44	44	45	
	Efficiency heat	%	41.9	43	46.5	50	52.3	54.7	54.7	54.7	
	Lifetime	years	30	30	30	30	30	30	30	30	
Waste incinerator	Capex	€/kW$_{el}$	5940	5630	5440	5240	5030	4870	4690	4540	
	Opex fix	€/(kW$_{el}$ a)	267.3	253.4	244.8	235.8	226.4	219.2	211.1	204.3	[4]
	Opex var	€/(kWh$_{el}$)	0.007	0.007	0.007	0.007	0.007	0.007	0.007	0.007	
	Efficiency elec.	%	24	26	26	26	26	26	26	26	
	Efficiency heat	%	65.5	71	71	71	71	71	71	71	
	Lifetime	years	30	30	30	30	30	30	30	30	
Biogas digester	Capex	€/kW$_{th}$	771	731	706	680	653	632	609	589	[12]
	Opex fix	€/(kW$_{th}$ a)	30.8	29.2	28.2	27.2	26.1	25.3	24.3	23.6	
	Opex var	€/(kWh$_{th}$)	0	0	0	0	0	0	0	0	
	Lifetime	years	20	20	20	20	25	25	25	25	
Biogas upgrade	Capex	€/kW$_{th}$	340	290	270	250	230	220	210	200	
	Opex fix	€/(kW$_{th}$ a)	27.2	23.2	21.6	20	18.4	17.6	16.8	16	
	Opex var	€/(kWh$_{th}$)	0	0	0	0	0	0	0	0	[12]
	Efficiency	%	98 %	98 %	98 %	98 %	98 %	98 %	98 %	98 %	
	Lifetime	years	20	20	20	20	25	25	25	25	
CSP (solar field, parabolic trough)	Capex	€/kW$_{th}$	438.3	344.5	303.6	274.7	251.1	230.2	211.9	196	[13,14]
	Opex fix	€/(kW$_{th}$ a)	10.1	7.9	7	6.3	5.8	5.3	4.9	4.5	

Technology		Unit	2015/ 2017	2020	2025	2030	2035	2040	2045	2050	Ref
	Opex var	€/(kWh$_{th}$)	0	0	0	0	0	0	0	0	
	Lifetime	years	25	25	25	25	25	25	25	25	
	Opex var	€/(kWh$_{th}$)	0	0	0	0	0	0	0	0	
	Efficiency	%	95	95	95	95	95	95	95	95	
	Lifetime	years	22	22	22	22	22	22	22	22	
Water electrolysis	Capex	€/kW$_{H2}$	800	685	500	363	325	296	267	248	
	Opex fix	€/(kW$_{H2}$ a)	32	27	20	12.7	11.4	10.4	9.4	8.7	15,16
	Opex var	€/(kWh$_{H2}$)	0.001	0.001	0.001	0.001	0.001	0.001	0.001	0.001	
	Efficiency	%	84	84	84	84	84	84	84	84	
	Lifetime	years	30	30	30	30	30	30	30	30	
Methanation	Capex	€/kW$_{CH4}$	547	502	368	278	247	226	204	190	
	Opex fix	€/(kW$_{CH4}$ a)	25.16	23.09	16.93	12.79	11.36	10.4	9.38	8.74	15,16
	Opex var	€/(kWh$_{CH4}$)	0.002	0.002	0.002	0.002	0.002	0.002	0.002	0.002	
	Efficiency	%	77	77	77	77	77	77	77	77	
	CO_2 input	kgCO_2/kWh$_{th}$	0.178	0.178	0.178	0.178	0.178	0.178	0.178	0.178	
	Lifetime	years	30	30	30	30	30	30	30	30	
CO_2 direct air capture	Capex	€/tCO_2 a	1000	730	493	335	274.4	234	210.6	195	
	Opex fix	€/tCO_2 a	40	29.2	19.7	13.4	11	9.4	8.4	7.8	17
	Opex var	€/tCO_2	0	0	0	0	0	0	0	0	
	Elec. cons	kWh$_{el}$/tCO_2	250	242	236	225	214	203	192	182	
	Heat cons	kWh$_{th}$/tCO_2	1750	1670	1590	1500	1393	1286	1194	1102	
	Lifetime	years	20	20	30	30	30	30	30	30	
Fischer-Tropsch unit	Capex	€/kW$_{FTLiq,output}$	947	947	947	947	947	852.3	852.3	852.3	
	Opex fix	€/kW$_{FTLiq,output}$	28.41	28.41	28.41	28.41	28.41	25.57	25.57	25.57	18
	Opex var	€/kW$_{FTLiq,output}$	0	0	0	0	0	0	0	0	
	Efficiency	%	0.634	0.634	0.634	0.634	0.634	0.634	0.634	0.634	
	CO_2 input	kgCO_2/kWh$_{th}$	0.284	0.284	0.284	0.284	0.284	0.284	0.284	0.284	
	Lifetime	years	30	30	30	30	30	30	30	30	
A-CAES	Capex	€/kWh$_{el}$	80.4	80.4	70.7	63.3	59.0	56.2	52.4	49.2	
	Opex fix	€/(kWh$_{el}$ a)	1	1	1	1	1	1	1	1	
	Opex var	€/(kWh$_{el}$)	0	0	0	0	0	0	0	0	
	Efficiency	%	54	59	65	70	70	70	70	70	
	Self-discharge	%/h	0.1	0.1	0.1	0.1	0.1	0.1	0.1	0.1	
	Lifetime	years	40	55	55	55	55	55	55	55	
Gas Storage	Capex	€/kWh$_{el}$	0.05	0.05	0.05	0.05	0.05	0.05	0.05	0.05	
	Opex fix	€/(kWh$_{el}$ a)	0.001	0.001	0.001	0.001	0.001	0.001	0.001	0.001	
	Opex var	€/(kWh$_{el}$)	0	0	0	0	0	0	0	0	
	Efficiency	%	100	100	100	100	100	100	100	100	
	Self-discharge	%/h	0	0	0	0	0	0	0	0	
	Lifetime	years	50	50	50	50	50	50	50	50	
Hydrogen Storage	Capex	€/kWh$_{th}$	0.24	0.24	0.24	0.24	0.24	0.24	0.24	0.24	
	Opex fix	€/(kWh$_{th}$ a)	0.01	0.01	0.01	0.01	0.01	0.01	0.01	0.01	20
	Opex var	€/(kWh$_{th}$)	0	0	0	0	0	0	0	0	
	Efficiency	%	100	100	100	100	100	100	100	100	
	Self-discharge	%/h	0	0	0	0	0	0	0	0	
	Lifetime	years	15	15	15	15	15	15	15	15	
CO_2 Storage	Capex	€/ton	142	142	142	142	142	142	142	142	
	Opex fix	€/(ton a)	9.94	9.94	9.94	9.94	9.94	9.94	9.94	9.94	21
	Opex var	€/ton	0	0	0	0	0	0	0	0	
	Efficiency	%	100	100	100	100	100	100	100	100	
	Self-discharge	%/h	0	0	0	0	0	0	0	0	
	Lifetime	years	30	30	30	30	30	30	30	30	

Supplementary Table 2: Ramping costs for power generation technologies. Data adopted from Deutsches Institut für Wirtschaftsforschung [22]. Core technologies are listed in Table 2 of the main manuscript file.

Technology	Unit	
Geothermal power	€/MW	0
Internal Combustion Generator	€/MW	0
Steam Turbine (CSP)	€/MW	0
CHP Biogas	€/MW	22.9

Waste incinerator	€/MW	54.3

Supplementary Table 3: Efficiency assumptions for HVAC and HVDC transmission lines [30].

Component	Power losses
HVAC line	9.4% / 1000 km
HVDC line	1.6% / 1000 km
HVDC converter pair	1.4%

Supplementary Table 4: Financial and technical assumptions for HVAC and HVDC transmission lines.

	Year		2015/2017	2020	2025	2030	2035	2040	2045	2050
HVDC Transmission Line Blend: 30% Overhead. 70% Cable	Capex	€/(kW·km)	0.92	0.92	0.92	0.92	0.92	1.05	1.05	1.05
	Opex fix	€/(kW·km)	0.00	0.00	0.00	0.00	0.00	0.00	0.00	0.00
	Opex var	€/(kWh·km)	0.00	0.00	0.00	0.00	0.00	0.00	0.00	0.00
	Lifetime	year	50	50	50	50	50	50	50	50
	Efficiency	Per 1000 km	0.98	0.98	0.98	0.98	0.98	0.98	0.98	0.98
HVDC Transmission Line (Cable)	Capex	€/(kW·km)	1.23	1.23	1.23	1.23	1.23	1.37	1.37	1.37
	Opex fix	€/(kW·km)	0.00	0.00	0.00	0.00	0.00	0.00	0.00	0.00
	Opex var	€/(kWh·km)	0.00	0.00	0.00	0.00	0.00	0.00	0.00	0.00
	Lifetime	year	50	50	50	50	50	50	50	50
	Efficiency	Per 1000 km	0.98	0.98	0.98	0.98	0.98	0.98	0.98	0.98
HVDC Transmission Line (Overhead)	Capex	€/(kW·km)	0.20	0.20	0.20	0.20	0.20	0.30	0.30	0.30
	Opex fix	€/(kW·km)	0.00	0.00	0.00	0.00	0.00	0.00	0.00	0.00
	Opex var	€/(kWh·km)	0.00	0.00	0.00	0.00	0.00	0.00	0.00	0.00
	Lifetime	year	50	50	50	50	50	50	50	50
	Efficiency	Per 1000 km	0.98	0.98	0.98	0.98	0.98	0.98	0.98	0.98
HVAC Transmission Line (Overhead)	Capex	€/(kW·km)	0.46	0.46	0.46	0.46	0.46	0.46	0.46	0.46
	Opex fix	€/(kW·km)	0.00	0.00	0.00	0.00	0.00	0.00	0.00	0.00
	Opex var	€/(kWh·km)	0.00	0.00	0.00	0.00	0.00	0.00	0.00	0.00
	Lifetime	year	50	50	50	50	50	50	50	50
	Efficiency	Per 1000 km	0.91	0.91	0.91	0.91	0.91	0.91	0.91	0.91
Converter Stations pair - HVDC	Capex	€/(kW)	150.00	150.00	150.00	150.00	150.00	180.00	180.00	180.00
	Opex fix	€/(kW)	1.50	1.50	1.50	1.50	1.50	1.80	1.80	1.80
	Opex var	€/(kWh·a)	0.00	0.00	0.00	0.00	0.00	0.00	0.00	0.00
	Lifetime	year	50	50	50	50	50	50	50	50
	Efficiency		0.99	0.99	0.99	0.99	0.99	0.99	0.99	0.99

Long term exchange rate used: 1 € = 75 INR.

Results

Supplementary Table 5: Installed capacities of power and storage technologies for each region from 2015 to 2050.

	Technology	Units	2015	2020	2025	2030	2035	2040	2045	2050
**Jammu Kashmir**	PV prosumers	GW	0	0	0	2	4	6	8	11
	PV fixed tilted	GW	0	0	0	0	0	0	0	4
	PV single-axis tracking	GW	0	0	3	3	3	5	9	9
	Concentrating Solar Thermal Power (CSP)	GW	0	0	0	0	0	0	0	0
	Wind energy	GW	0	0	0	3	8	8	8	8
	Hydropower Run-of-River	GW	2	2	2	2	2	2	2	2
	Hydropower reservoir (dam)	GW	1	2	3	3	3	3	3	3
	Geothermal power	GW	0	0	0	0	0	0	0	0
	Combined Cycle Gas Turbine (CCGT)	GW	0	0	0	0	0	0	0	0
	Open Cycle Gas Turbine (OCGT)	GW	0	0	0	0	0	0	0	0
	Heavy Duty Open Cycle Gas Turbine (OCGT HD)	GW	0	0	0	0	0	0	0	0
	Biomass PP	GW	0	0	0	0	0	0	0	0
	Waste PP	GW	0	0	0	0	0	0	0	0
	Biogas PP	GW	0	0	0	0	0	0	0	0
	Hard coal PP	GW	0	1	1	1	0	0	0	0
	Internal combustion generator (ICG)	GW	0	0	0	0	1	1	1	1
	Multifuel Internal combustion generator	GW	0	0	0	0	0	0	0	0
	Nuclear PP	GW	0	0	0	0	0	0	0	0
	Battery storage	GWh	0	0	0	8	16	26	43	61
	PHES storage	GWh	0	0	0	0	0	0	0	0
	Thermal Energy Storage (TES)	GWh	0	0	0	0	0	0	0	0
	Adiabatic Compressed Air Energy Storage (A-CAES)	GWh	0	0	0	0	0	0	0	0
	Power-to-Gas	GW_{el}	0	0	0	0	0	0	0	0
	Gas storage	GWh	1	1	3	261	264	295	296	300
**Himachal Pradesh**	PV prosumers	GW	0	0	0	2	3	4	6	8
	PV fixed tilted	GW	0	0	0	0	0	0	0	28
	PV single-axis tracking	GW	0	0	28	28	28	43	58	58
	Concentrating Solar Thermal Power (CSP)	GW	0	0	0	0	0	0	0	0
	Wind energy	GW	0	0	6	13	19	19	19	19
	Hydropower Run-of-River	GW	0	0	0	0	0	0	0	0
	Hydropower reservoir (dam)	GW	9	10	15	15	15	15	15	15
	Geothermal power	GW	0	0	0	0	0	0	0	0
	Combined Cycle Gas Turbine (CCGT)	GW	0	0	0	0	0	0	0	0
	Open Cycle Gas Turbine (OCGT)	GW	0	0	0	0	0	0	0	0
	Heavy Duty Open Cycle Gas Turbine (OCGT HD)	GW	0	0	0	0	0	0	0	0
	Biomass PP	GW	0	1	1	1	1	1	0	0

	Waste PP	GW	0	0	0	0	0	0	0	0
	Biogas PP	GW	0	0	0	0	0	0	0	0
	Hard coal PP	GW	0	0	0	0	0	0	0	0
	Internal combustion generator (ICG)	GW	0	0	0	0	0	1	1	1
	Multifuel Internal combustion generator	GW	0	0	0	0	0	0	0	0
	Nuclear PP	GW	0	0	0	0	0	0	0	0
	Battery storage	GWh	0	0	0	17	46	96	151	241
	PHES storage	GWh	0	0	0	0	0	0	0	0
	Thermal Energy Storage (TES)	GWh	0	0	0	0	0	0	0	0
	Adiabatic Compressed Air Energy Storage (A-CAES)	GWh	0	0	0	0	0	0	0	0
	Power-to-Gas	GW_{el}	0	0	0	0	0	0	0	0
	Gas storage	GWh	1	1	3	58	67	68	69	71
**Uttarakhand**	PV prosumers	GW	0	0	0	2	4	6	9	11
	PV fixed tilted	GW	0	0	0	0	2	2	2	9
	PV single-axis tracking	GW	0	0	2	3	3	5	9	9
	Concentrating Solar Thermal Power (CSP)	GW	0	0	0	0	0	0	0	0
	Wind energy	GW	0	0	0	0	0	0	0	0
	Hydropower Run-of-River	GW	2	3	3	3	3	3	3	3
	Hydropower reservoir (dam)	GW	1	1	2	2	2	2	2	2
	Geothermal power	GW	0	0	0	0	0	0	0	0
	Combined Cycle Gas Turbine (CCGT)	GW	0	1	1	1	0	0	0	0
	Open Cycle Gas Turbine (OCGT)	GW	0	0	0	0	0	0	0	0
	Heavy Duty Open Cycle Gas Turbine (OCGT HD)	GW	0	0	0	0	0	0	0	0
	Biomass PP	GW	0	1	1	1	1	1	0	0
	Waste PP	GW	0	0	0	0	0	0	0	0
	Biogas PP	GW	0	0	0	0	0	0	0	0
	Hard coal PP	GW	0	0	0	0	0	0	0	0
	Internal combustion generator (ICG)	GW	0	0	0	0	1	1	1	1
	Multifuel Internal combustion generator	GW	0	0	0	0	0	0	0	0
	Nuclear PP	GW	0	0	0	0	0	0	0	0
	Battery storage	GWh	0	0	0	8	18	27	42	71
	PHES storage	GWh	0	0	0	0	0	0	0	0
	Thermal Energy Storage (TES)	GWh	0	0	0	0	0	0	0	0
	Adiabatic Compressed Air Energy Storage (A-CAES)	GWh	0	0	0	0	0	0	0	0
	Power-to-Gas	GW_{el}	0	0	0	0	0	0	0	0
	Gas storage	GWh	1	1	2	102	108	118	120	124
**Punjab & Chandigarh**	PV prosumers	GW	0	0	4	11	19	28	38	51
	PV fixed tilted	GW	0	1	1	1	1	1	1	1
	PV single-axis tracking	GW	0	0	0	0	0	0	0	0
	Concentrating Solar Thermal Power (CSP)	GW	0	0	0	0	0	0	0	0
	Wind energy	GW	0	0	1	15	17	17	17	17
	Hydropower Run-of-River	GW	0	0	0	0	0	0	0	0

	Hydropower reservoir (dam)	GW	1	1	2	2	2	2	2	2
	Geothermal power	GW	0	0	0	0	0	0	0	0
	Combined Cycle Gas Turbine (CCGT)	GW	0	0	0	0	0	0	0	0
	Open Cycle Gas Turbine (OCGT)	GW	0	0	0	0	0	0	0	0
	Heavy Duty Open Cycle Gas Turbine (OCGT HD)	GW	0	0	0	0	0	0	0	0
	Biomass PP	GW	0	1	3	5	6	6	5	5
	Waste PP	GW	0	0	0	0	0	0	0	0
	Biogas PP	GW	0	0	0	0	0	0	0	0
	Hard coal PP	GW	7	8	8	8	7	7	6	5
	Internal combustion generator (ICG)	GW	0	0	0	1	2	3	4	5
	Multifuel Internal combustion generator	GW	0	0	0	0	0	0	0	0
	Nuclear PP	GW	0	0	0	0	0	0	0	0
	Battery storage	GWh	0	0	9	27	44	71	89	117
	PHES storage	GWh	0	0	0	0	0	0	0	0
	Thermal Energy Storage (TES)	GWh	0	0	0	0	0	0	0	0
	Adiabatic Compressed Air Energy Storage (A-CAES)	GWh	0	0	0	0	0	0	0	0
	Power-to-Gas	GW_{el}	0	0	0	0	0	0	0	0
	Gas storage	GWh	1	1	12	640	828	858	859	921
**Haryana**	PV prosumers	GW	0	0	4	10	17	26	35	49
	PV fixed tilted	GW	0	0	0	0	0	7	34	79
	PV single-axis tracking	GW	0	0	0	0	0	0	0	0
	Concentrating Solar Thermal Power (CSP)	GW	0	0	0	0	0	0	0	0
	Wind energy	GW	0	0	14	15	15	15	15	15
	Hydropower Run-of-River	GW	0	0	0	0	0	0	0	0
	Hydropower reservoir (dam)	GW	0	0	0	0	0	0	0	0
	Geothermal power	GW	0	0	0	0	0	0	0	0
	Combined Cycle Gas Turbine (CCGT)	GW	1	1	1	1	1	1	0	0
	Open Cycle Gas Turbine (OCGT)	GW	0	0	0	0	0	0	0	0
	Heavy Duty Open Cycle Gas Turbine (OCGT HD)	GW	0	0	0	0	0	0	0	0
	Biomass PP	GW	0	1	1	3	3	3	3	3
	Waste PP	GW	0	0	0	0	0	0	0	0
	Biogas PP	GW	0	0	0	0	0	0	0	0
	Hard coal PP	GW	8	8	8	8	8	7	7	6
	Internal combustion generator (ICG)	GW	0	0	0	1	2	2	3	4
	Multifuel Internal combustion generator	GW	0	0	4	4	4	4	4	4
	Nuclear PP	GW	0	0	0	0	0	0	0	0
	Battery storage	GWh	0	0	8	42	56	84	151	267
	PHES storage	GWh	0	0	0	0	0	0	0	0
	Thermal Energy Storage (TES)	GWh	0	0	0	0	0	0	0	0
	Adiabatic Compressed Air Energy Storage (A-CAES)	GWh	0	0	0	0	0	0	0	0
	Power-to-Gas	GW_{el}	0	0	0	1	1	1	1	1
	Gas storage	GWh	1	1	5	1421	1962	1964	1968	2235

**Delhi**	PV prosumers	GW	0	0	3	7	11	14	16	17
	PV fixed tilted	GW	0	0	0	7	7	7	7	7
	PV single-axis tracking	GW	0	0	0	0	3	7	7	7
	Concentrating Solar Thermal Power (CSP)	GW	0	0	0	0	0	0	0	0
	Wind energy	GW	0	0	0	0	0	0	0	0
	Hydropower Run-of-River	GW	0	0	0	0	0	0	0	0
	Hydropower reservoir (dam)	GW	0	0	0	0	0	0	0	0
	Geothermal power	GW	0	0	0	0	0	0	0	0
	Combined Cycle Gas Turbine (CCGT)	GW	2	2	2	2	2	2	2	2
	Open Cycle Gas Turbine (OCGT)	GW	0	0	0	0	0	0	0	0
	Heavy Duty Open Cycle Gas Turbine (OCGT HD)	GW	0	0	0	0	0	0	0	0
	Biomass PP	GW	0	0	0	0	0	0	0	0
	Waste PP	GW	0	0	0	0	0	0	0	0
	Biogas PP	GW	0	0	0	0	0	0	0	0
	Hard coal PP	GW	4	4	4	4	4	3	3	2
	Internal combustion generator (ICG)	GW	0	0	0	0	1	2	2	3
	Multifuel Internal combustion generator	GW	0	0	10	10	10	10	10	10
	Nuclear PP	GW	0	0	0	0	0	0	0	0
	Battery storage	GWh	0	0	6	28	43	64	80	90
	PHES storage	GWh	0	0	0	0	0	0	0	0
	Thermal Energy Storage (TES)	GWh	0	0	0	0	0	0	0	0
	Adiabatic Compressed Air Energy Storage (A-CAES)	GWh	0	0	0	0	0	0	0	0
	Power-to-Gas	GW_{el}	0	0	0	2	2	2	2	2
	Gas storage	GWh	1	1	2	2852	3649	3734	3734	4219
**Rajasthan**	PV prosumers	GW	0	1	6	14	23	34	45	60
	PV fixed tilted	GW	1	5	5	5	20	79	104	220
	PV single-axis tracking	GW	0	0	6	35	46	50	107	107
	Concentrating Solar Thermal Power (CSP)	GW	0	0	0	0	0	0	0	0
	Wind energy	GW	3	4	21	22	26	25	24	23
	Hydropower Run-of-River	GW	0	0	0	0	0	0	0	0
	Hydropower reservoir (dam)	GW	1	1	1	1	2	2	2	2
	Geothermal power	GW	0	0	0	0	0	0	0	0
	Combined Cycle Gas Turbine (CCGT)	GW	0	0	0	0	0	0	0	0
	Open Cycle Gas Turbine (OCGT)	GW	1	1	1	1	1	1	1	1
	Heavy Duty Open Cycle Gas Turbine (OCGT HD)	GW	0	0	0	0	0	0	0	0
	Biomass PP	GW	0	1	1	2	3	3	2	2
	Waste PP	GW	0	0	0	0	0	0	0	0
	Biogas PP	GW	0	0	0	0	0	0	0	0
	Hard coal PP	GW	8	10	10	10	10	10	9	8
	Internal combustion generator (ICG)	GW	0	0	0	1	2	3	3	5
	Multifuel Internal combustion generator	GW	0	0	15	15	15	15	15	15
	Nuclear PP	GW	1	1	1	1	1	1	1	0

	Battery storage	GWh	0	0	15	71	202	379	677	1067
	PHES storage	GWh	0	0	0	0	0	0	0	0
	Thermal Energy Storage (TES)	GWh	0	0	0	0	0	0	0	0
	Adiabatic Compressed Air Energy Storage (A-CAES)	GWh	0	0	0	0	0	0	0	0
	Power-to-Gas	GW_{el}	0	0	0	4	4	4	10	12
	Gas storage	GWh	1	1	9	4927	6255	7089	15451	19326
**Uttar Pradesh**	PV prosumers	GW	0	1	8	21	36	54	72	95
	PV fixed tilted	GW	0	1	1	32	51	90	132	186
	PV single-axis tracking	GW	0	0	14	31	31	31	31	31
	Concentrating Solar Thermal Power (CSP)	GW	0	0	0	0	0	0	0	0
	Wind energy	GW	0	0	18	23	23	23	23	23
	Hydropower Run-of-River	GW	0	0	0	0	0	0	0	0
	Hydropower reservoir (dam)	GW	0	0	0	0	1	1	1	1
	Geothermal power	GW	0	0	0	0	0	0	0	0
	Combined Cycle Gas Turbine (CCGT)	GW	1	1	1	1	0	0	0	0
	Open Cycle Gas Turbine (OCGT)	GW	0	0	0	0	0	0	0	0
	Heavy Duty Open Cycle Gas Turbine (OCGT HD)	GW	0	0	0	0	0	0	0	0
	Biomass PP	GW	0	1	2	3	4	4	3	3
	Waste PP	GW	0	0	0	0	0	0	0	0
	Biogas PP	GW	0	0	0	0	0	0	0	0
	Hard coal PP	GW	15	24	24	23	20	18	17	17
	Internal combustion generator (ICG)	GW	0	0	0	1	4	5	7	8
	Multifuel Internal combustion generator	GW	0	0	28	28	28	28	28	28
	Nuclear PP	GW	0	0	0	0	0	0	0	0
	Battery storage	GWh	0	0	17	138	241	399	564	771
	PHES storage	GWh	0	0	0	0	0	0	0	0
	Thermal Energy Storage (TES)	GWh	0	0	0	0	0	0	0	0
	Adiabatic Compressed Air Energy Storage (A-CAES)	GWh	0	0	0	0	0	0	0	0
	Power-to-Gas	GW_{el}	0	0	0	6	6	6	6	6
	Gas storage	GWh	1	1	6	7972	9995	9995	9995	10189
**Madhya Pradesh**	PV prosumers	GW	0	0	5	12	21	34	50	69
	PV fixed tilted	GW	1	2	2	2	11	14	14	47
	PV single-axis tracking	GW	0	0	0	5	5	5	6	6
	Concentrating Solar Thermal Power (CSP)	GW	0	0	0	0	0	0	0	0
	Wind energy	GW	1	3	15	29	29	29	34	32
	Hydropower Run-of-River	GW	0	0	0	0	0	0	0	0
	Hydropower reservoir (dam)	GW	2	2	2	2	3	3	3	3
	Geothermal power	GW	0	0	0	0	0	0	0	0
	Combined Cycle Gas Turbine (CCGT)	GW	0	0	0	0	0	0	0	0
	Open Cycle Gas Turbine (OCGT)	GW	0	0	0	0	0	0	0	0
	Heavy Duty Open Cycle Gas Turbine (OCGT HD)	GW	0	0	0	0	0	0	0	0
	Biomass PP	GW	0	1	1	2	3	3	3	3

	Waste PP	GW	0	0	0	0	0	0	0	0
	Biogas PP	GW	0	0	0	0	0	0	0	0
	Hard coal PP	GW	15	22	22	21	20	19	18	18
	Internal combustion generator (ICG)	GW	0	0	1	1	3	3	4	5
	Multifuel Internal combustion generator	GW	0	0	0	1	1	1	1	3
	Nuclear PP	GW	0	0	0	0	0	0	0	0
	Battery storage	GWh	0	0	10	41	78	114	153	224
	PHES storage	GWh	0	0	0	0	0	0	0	0
	Thermal Energy Storage (TES)	GWh	0	0	0	0	0	1	1	1
	Adiabatic Compressed Air Energy Storage (A-CAES)	GWh	0	0	0	0	0	0	0	0
	Power-to-Gas	GW_{el}	0	0	0	0	0	0	0	2
	Gas storage	GWh	0	8	10	133	728	739	744	2249
**Gujarat, Daman & Dadra**	PV prosumers	GW	0	1	4	20	38	58	76	100
	PV fixed tilted	GW	1	2	2	2	13	13	13	12
	PV single-axis tracking	GW	0	0	3	28	28	46	62	112
	Concentrating Solar Thermal Power (CSP)	GW	0	0	0	0	0	0	0	0
	Wind energy	GW	4	7	29	46	50	54	66	66
	Hydropower Run-of-River	GW	0	0	0	0	0	0	0	0
	Hydropower reservoir (dam)	GW	1	1	1	1	1	1	1	1
	Geothermal power	GW	0	0	0	0	0	0	0	0
	Combined Cycle Gas Turbine (CCGT)	GW	7	7	7	7	6	5	4	4
	Open Cycle Gas Turbine (OCGT)	GW	1	1	1	1	1	1	1	4
	Heavy Duty Open Cycle Gas Turbine (OCGT HD)	GW	0	0	0	0	0	0	0	0
	Biomass PP	GW	0	1	1	2	3	3	2	2
	Waste PP	GW	0	0	0	0	0	0	0	0
	Biogas PP	GW	0	0	0	0	0	0	0	0
	Hard coal PP	GW	18	20	19	19	18	17	16	16
	Internal combustion generator (ICG)	GW	0	0	1	3	4	6	8	10
	Multifuel Internal combustion generator	GW	0	0	1	3	3	3	3	5
	Nuclear PP	GW	0	0	0	0	0	0	0	0
	Battery storage	GWh	0	0	5	106	198	293	363	519
	PHES storage	GWh	0	0	0	0	0	0	0	0
	Thermal Energy Storage (TES)	GWh	0	0	0	0	0	1	1	1
	Adiabatic Compressed Air Energy Storage (A-CAES)	GWh	0	0	0	0	0	0	0	0
	Power-to-Gas	GW_{el}	0	0	0	1	2	2	4	6
	Gas storage	GWh	0	8	3	210	3134	3136	4429	7170
**Maharashtra & Goa**	PV prosumers	GW	0	1	13	35	59	91	125	168
	PV fixed tilted	GW	0	1	1	1	52	52	52	52
	PV single-axis tracking	GW	0	0	2	44	44	59	73	73
	Concentrating Solar Thermal Power (CSP)	GW	0	0	0	0	0	0	0	0
	Wind energy	GW	4	5	23	35	36	43	57	84
	Hydropower Run-of-River	GW	0	0	0	0	0	0	0	0

	Hydropower reservoir (dam)	GW	3	3	3	4	4	4	4	4
	Geothermal power	GW	0	0	0	0	0	0	0	0
	Combined Cycle Gas Turbine (CCGT)	GW	3	4	4	3	3	3	1	1
	Open Cycle Gas Turbine (OCGT)	GW	0	0	0	0	0	0	0	0
	Heavy Duty Open Cycle Gas Turbine (OCGT HD)	GW	0	0	0	0	0	0	0	0
	Biomass PP	GW	0	1	2	3	5	5	5	4
	Waste PP	GW	0	0	0	0	0	0	0	0
	Biogas PP	GW	0	0	0	0	0	0	0	1
	Hard coal PP	GW	19	27	27	26	25	24	23	22
	Internal combustion generator (ICG)	GW	0	0	1	3	6	8	10	12
	Multifuel Internal combustion generator	GW	0	0	1	3	3	3	3	5
	Nuclear PP	GW	1	1	1	1	1	1	1	1
	Battery storage	GWh	0	0	27	163	331	478	610	672
	PHES storage	GWh	0	0	8	8	8	8	8	8
	Thermal Energy Storage (TES)	GWh	0	0	0	0	0	2	2	2
	Adiabatic Compressed Air Energy Storage (A-CAES)	GWh	0	0	0	0	0	0	0	0
	Power-to-Gas	GW_{el}	0	0	0	0	1	1	3	5
	Gas storage	GWh	0	8	8	223	2834	2896	3911	6108
**Chhattisgarh**	PV prosumers	GW	0	0	1	5	8	13	20	28
	PV fixed tilted	GW	0	0	0	0	17	17	17	17
	PV single-axis tracking	GW	0	0	0	5	5	9	16	21
	Concentrating Solar Thermal Power (CSP)	GW	0	0	0	0	0	0	0	0
	Wind energy	GW	0	0	3	17	17	17	20	20
	Hydropower Run-of-River	GW	0	0	0	0	0	0	0	0
	Hydropower reservoir (dam)	GW	0	0	0	0	0	0	0	0
	Geothermal power	GW	0	0	0	0	0	0	0	0
	Combined Cycle Gas Turbine (CCGT)	GW	0	0	0	0	0	0	0	0
	Open Cycle Gas Turbine (OCGT)	GW	0	0	0	0	0	0	0	0
	Heavy Duty Open Cycle Gas Turbine (OCGT HD)	GW	0	0	0	0	0	0	0	0
	Biomass PP	GW	0	1	1	1	1	1	1	1
	Waste PP	GW	0	0	0	0	0	0	0	0
	Biogas PP	GW	0	0	0	0	0	0	0	0
	Hard coal PP	GW	17	28	28	27	25	24	24	24
	Internal combustion generator (ICG)	GW	0	0	0	1	1	1	2	2
	Multifuel Internal combustion generator	GW	0	0	0	1	1	1	1	2
	Nuclear PP	GW	0	0	0	0	0	0	0	0
	Battery storage	GWh	0	0	0	44	75	103	133	177
	PHES storage	GWh	0	0	0	0	0	0	0	0
	Thermal Energy Storage (TES)	GWh	0	0	0	0	0	1	1	1
	Adiabatic Compressed Air Energy Storage (A-CAES)	GWh	0	0	0	0	0	0	0	0
	Power-to-Gas	GW_{el}	0	0	0	0	0	0	1	1
	Gas storage	GWh	0	8	5	76	396	423	646	1693

**Kerala**	PV prosumers	GW	0	0	2	5	9	14	19	26
	PV fixed tilted	GW	0	0	0	0	0	0	0	0
	PV single-axis tracking	GW	0	0	5	10	10	13	22	24
	Concentrating Solar Thermal Power (CSP)	GW	0	0	0	0	0	0	0	0
	Wind energy	GW	0	0	3	4	4	5	5	9
	Hydropower Run-of-River	GW	0	0	0	0	0	0	0	0
	Hydropower reservoir (dam)	GW	2	2	3	3	3	3	3	3
	Geothermal power	GW	0	0	0	0	0	0	0	0
	Combined Cycle Gas Turbine (CCGT)	GW	1	1	1	1	1	0	0	0
	Open Cycle Gas Turbine (OCGT)	GW	0	0	0	0	0	0	0	0
	Heavy Duty Open Cycle Gas Turbine (OCGT HD)	GW	0	0	0	0	0	0	0	0
	Biomass PP	GW	0	1	1	2	2	2	1	1
	Waste PP	GW	0	0	0	0	0	0	0	0
	Biogas PP	GW	0	0	0	0	0	0	0	0
	Hard coal PP	GW	2	2	2	2	2	1	1	1
	Internal combustion generator (ICG)	GW	0	0	0	1	1	1	2	2
	Multifuel Internal combustion generator	GW	0	0	1	1	1	1	1	1
	Nuclear PP	GW	0	0	0	0	0	0	0	0
	Battery storage	GWh	0	0	4	28	38	61	96	121
	PHES storage	GWh	0	0	0	0	0	0	0	0
	Thermal Energy Storage (TES)	GWh	0	0	0	0	0	0	0	1
	Adiabatic Compressed Air Energy Storage (A-CAES)	GWh	0	0	0	0	0	0	0	0
	Power-to-Gas	GW_{el}	0	0	0	0	0	0	0	0
	Gas storage	GWh	0	3	22	322	620	813	874	931
**Tamil Nadu & Puducherry**	PV prosumers	GW	0	1	9	21	37	55	74	98
	PV fixed tilted	GW	0	4	4	4	4	4	4	4
	PV single-axis tracking	GW	0	0	15	40	40	57	84	101
	Concentrating Solar Thermal Power (CSP)	GW	0	0	0	0	0	0	0	0
	Wind energy	GW	7	9	18	23	25	33	31	33
	Hydropower Run-of-River	GW	0	0	0	0	0	0	0	0
	Hydropower reservoir (dam)	GW	2	2	2	3	3	3	3	3
	Geothermal power	GW	0	0	0	0	0	0	0	0
	Combined Cycle Gas Turbine (CCGT)	GW	2	2	2	2	1	1	0	0
	Open Cycle Gas Turbine (OCGT)	GW	0	0	0	0	0	0	0	7
	Heavy Duty Open Cycle Gas Turbine (OCGT HD)	GW	0	0	0	0	0	0	0	1
	Biomass PP	GW	0	1	1	3	3	3	3	2
	Waste PP	GW	0	0	0	0	0	0	0	0
	Biogas PP	GW	0	0	0	0	0	0	0	1
	Hard coal PP	GW	10	14	14	14	12	11	10	9
	Internal combustion generator (ICG)	GW	3	3	4	5	5	6	7	9
	Multifuel Internal combustion generator	GW	0	0	3	3	3	3	3	4
	Nuclear PP	GW	1	2	2	2	2	2	2	2

	Battery storage	GWh	0	0	19	107	150	220	298	368
	PHES storage	GWh	0	0	6	6	6	6	6	6
	Thermal Energy Storage (TES)	GWh	0	0	0	0	0	0	0	2
	Adiabatic Compressed Air Energy Storage (A-CAES)	GWh	0	0	0	0	0	0	0	0
	Power-to-Gas	GW_{el}	0	0	0	1	1	2	3	3
	Gas storage	GWh	0	4	18	1199	2539	3614	4990	6397
**Karnataka**	PV prosumers	GW	0	1	6	15	25	38	50	67
	PV fixed tilted	GW	0	7	7	7	7	34	71	152
	PV single-axis tracking	GW	0	0	11	44	44	44	62	62
	Concentrating Solar Thermal Power (CSP)	GW	0	0	0	0	0	0	0	0
	Wind energy	GW	3	5	13	13	12	11	11	9
	Hydropower Run-of-River	GW	1	1	1	1	1	1	1	1
	Hydropower reservoir (dam)	GW	4	4	6	6	6	6	6	6
	Geothermal power	GW	0	0	0	0	0	0	0	0
	Combined Cycle Gas Turbine (CCGT)	GW	1	1	1	1	1	1	0	0
	Open Cycle Gas Turbine (OCGT)	GW	0	0	0	0	0	0	0	0
	Heavy Duty Open Cycle Gas Turbine (OCGT HD)	GW	0	0	0	0	0	0	0	0
	Biomass PP	GW	0	1	1	3	3	3	2	2
	Waste PP	GW	0	0	0	0	0	0	0	0
	Biogas PP	GW	0	0	0	0	0	0	0	0
	Hard coal PP	GW	5	10	10	10	10	9	9	8
	Internal combustion generator (ICG)	GW	0	0	1	2	3	3	4	5
	Multifuel Internal combustion generator	GW	0	0	2	2	2	2	2	2
	Nuclear PP	GW	1	1	1	1	1	1	1	0
	Battery storage	GWh	0	0	14	108	162	281	470	735
	PHES storage	GWh	0	0	0	0	0	0	0	0
	Thermal Energy Storage (TES)	GWh	0	0	0	0	0	0	0	2
	Adiabatic Compressed Air Energy Storage (A-CAES)	GWh	0	0	0	0	0	0	0	0
	Power-to-Gas	GW_{el}	0	0	0	1	1	1	2	2
	Gas storage	GWh	0	3	24	682	1384	2248	3127	3378
**Andhra Pradesh**	PV prosumers	GW	0	0	5	12	21	32	43	58
	PV fixed tilted	GW	0	3	3	3	3	4	30	61
	PV single-axis tracking	GW	0	0	9	37	37	37	37	37
	Concentrating Solar Thermal Power (CSP)	GW	0	0	0	0	0	0	0	0
	Wind energy	GW	1	4	11	15	15	15	14	18
	Hydropower Run-of-River	GW	0	0	0	0	0	0	0	0
	Hydropower reservoir (dam)	GW	2	2	3	3	3	3	3	3
	Geothermal power	GW	0	0	0	0	0	0	0	0
	Combined Cycle Gas Turbine (CCGT)	GW	4	5	5	5	5	4	3	2
	Open Cycle Gas Turbine (OCGT)	GW	0	1	1	1	1	1	0	0
	Heavy Duty Open Cycle Gas Turbine (OCGT HD)	GW	0	0	0	0	0	0	0	0
	Biomass PP	GW	0	1	1	1	1	1	1	1

	Waste PP	GW	0	0	0	0	0	0	0	0
	Biogas PP	GW	0	0	0	0	0	0	0	1
	Hard coal PP	GW	6	11	11	11	10	10	10	9
	Internal combustion generator (ICG)	GW	0	0	0	1	2	3	4	5
	Multifuel Internal combustion generator	GW	0	0	2	2	2	2	2	2
	Nuclear PP	GW	0	0	0	0	0	0	0	0
	Battery storage	GWh	0	0	10	86	116	157	236	328
	PHES storage	GWh	0	0	0	0	0	0	0	0
	Thermal Energy Storage (TES)	GWh	0	0	0	0	1	1	1	2
	Adiabatic Compressed Air Energy Storage (A-CAES)	GWh	0	0	0	0	0	0	0	0
	Power-to-Gas	GW_{el}	0	0	0	1	1	1	2	2
	Gas storage	GWh	0	5	23	968	2365	2685	4970	5004
**Telangana**	PV prosumers	GW	0	0	4	12	20	30	42	57
	PV fixed tilted	GW	0	4	4	4	4	21	49	89
	PV single-axis tracking	GW	0	0	13	46	46	46	46	46
	Concentrating Solar Thermal Power (CSP)	GW	0	0	0	0	0	0	0	0
	Wind energy	GW	0	0	14	14	14	14	14	14
	Hydropower Run-of-River	GW	0	0	0	0	0	0	0	0
	Hydropower reservoir (dam)	GW	2	2	2	2	2	2	2	2
	Geothermal power	GW	0	0	0	0	0	0	0	0
	Combined Cycle Gas Turbine (CCGT)	GW	1	1	1	1	1	0	0	0
	Open Cycle Gas Turbine (OCGT)	GW	0	0	0	0	0	0	0	0
	Heavy Duty Open Cycle Gas Turbine (OCGT HD)	GW	0	0	0	0	0	0	0	1
	Biomass PP	GW	0	1	1	1	1	1	1	1
	Waste PP	GW	0	0	0	0	0	0	0	0
	Biogas PP	GW	0	0	0	0	0	0	0	0
	Hard coal PP	GW	6	8	8	7	6	6	5	5
	Internal combustion generator (ICG)	GW	0	0	0	1	2	3	4	5
	Multifuel Internal combustion generator	GW	0	0	2	2	2	2	2	2
	Nuclear PP	GW	0	0	0	0	0	0	0	0
	Battery storage	GWh	0	0	9	88	125	215	320	482
	PHES storage	GWh	1	1	14	14	14	14	14	14
	Thermal Energy Storage (TES)	GWh	0	0	0	0	1	1	1	2
	Adiabatic Compressed Air Energy Storage (A-CAES)	GWh	0	0	0	0	0	0	0	0
	Power-to-Gas	GW_{el}	0	0	0	1	1	2	2	2
	Gas storage	GWh	0	3	19	586	1694	2577	3374	3434
**Odisha**	PV prosumers	GW	0	0	2	6	11	16	23	31
	PV fixed tilted	GW	0	0	0	0	20	33	33	53
	PV single-axis tracking	GW	0	0	0	5	21	21	31	31
	Concentrating Solar Thermal Power (CSP)	GW	0	0	0	0	0	0	0	0
	Wind energy	GW	0	0	1	1	1	1	1	1
	Hydropower Run-of-River	GW	0	0	0	0	0	0	0	0

	Hydropower reservoir (dam)	GW	2	2	2	3	3	3	3	3
	Geothermal power	GW	0	0	0	0	0	0	0	0
	Combined Cycle Gas Turbine (CCGT)	GW	0	0	0	0	0	0	0	0
	Open Cycle Gas Turbine (OCGT)	GW	0	0	0	0	0	0	0	3
	Heavy Duty Open Cycle Gas Turbine (OCGT HD)	GW	0	0	0	0	0	0	0	0
	Biomass PP	GW	0	0	0	1	1	1	1	1
	Waste PP	GW	0	0	0	0	0	0	0	0
	Biogas PP	GW	0	0	0	0	0	0	0	0
	Hard coal PP	GW	13	17	16	16	15	14	14	12
	Internal combustion generator (ICG)	GW	0	0	0	1	1	2	2	2
	Multifuel Internal combustion generator	GW	0	0	0	0	0	0	0	4
	Nuclear PP	GW	0	0	0	0	0	0	0	0
	Battery storage	GWh	0	0	4	26	106	147	183	261
	PHES storage	GWh	0	0	0	0	0	0	0	0
	Thermal Energy Storage (TES)	GWh	0	0	0	0	0	0	0	0
	Adiabatic Compressed Air Energy Storage (A-CAES)	GWh	0	0	0	0	0	0	0	0
	Power-to-Gas	GW_{el}	0	0	0	0	0	0	0	3
	Gas storage	GWh	65	65	65	95	642	653	817	5511
**West Bengal & Sikkim**	PV prosumers	GW	0	1	5	14	24	37	51	68
	PV fixed tilted	GW	0	0	0	0	19	34	54	76
	PV single-axis tracking	GW	0	0	0	18	18	18	18	18
	Concentrating Solar Thermal Power (CSP)	GW	0	0	0	0	0	0	0	0
	Wind energy	GW	0	0	6	10	10	10	10	10
	Hydropower Run-of-River	GW	1	3	3	3	3	3	3	3
	Hydropower reservoir (dam)	GW	1	1	2	2	2	2	2	2
	Geothermal power	GW	0	0	1	2	2	2	2	2
	Combined Cycle Gas Turbine (CCGT)	GW	0	0	0	0	0	0	0	0
	Open Cycle Gas Turbine (OCGT)	GW	0	0	0	0	0	0	0	4
	Heavy Duty Open Cycle Gas Turbine (OCGT HD)	GW	0	0	0	0	0	0	0	0
	Biomass PP	GW	0	0	0	1	1	1	1	1
	Waste PP	GW	0	0	0	0	0	0	0	0
	Biogas PP	GW	0	0	0	0	0	0	0	0
	Hard coal PP	GW	11	14	14	13	12	11	9	9
	Internal combustion generator (ICG)	GW	0	0	1	1	3	3	4	5
	Multifuel Internal combustion generator	GW	0	0	2	2	2	2	2	2
	Nuclear PP	GW	0	0	0	0	0	0	0	0
	Battery storage	GWh	0	0	10	57	120	188	279	376
	PHES storage	GWh	0	0	0	0	0	0	0	0
	Thermal Energy Storage (TES)	GWh	0	0	0	0	0	0	0	0
	Adiabatic Compressed Air Energy Storage (A-CAES)	GWh	0	0	0	0	0	0	0	0
	Power-to-Gas	GW_{el}	0	0	0	0	2	2	2	2
	Gas storage	GWh	65	65	65	273	3481	3500	3500	3607

**Jharkhand**	PV prosumers	GW	0	0	1	3	6	10	14	19
	PV fixed tilted	GW	0	0	0	0	16	22	31	48
	PV single-axis tracking	GW	0	0	0	1	1	1	1	1
	Concentrating Solar Thermal Power (CSP)	GW	0	0	0	0	0	0	0	0
	Wind energy	GW	0	0	2	3	3	3	3	3
	Hydropower Run-of-River	GW	0	0	0	0	0	0	0	0
	Hydropower reservoir (dam)	GW	0	0	0	0	0	0	0	0
	Geothermal power	GW	0	0	0	0	0	0	0	0
	Combined Cycle Gas Turbine (CCGT)	GW	0	0	0	0	0	0	0	0
	Open Cycle Gas Turbine (OCGT)	GW	0	0	0	0	0	0	0	0
	Heavy Duty Open Cycle Gas Turbine (OCGT HD)	GW	0	0	0	0	0	0	0	0
	Biomass PP	GW	0	0	0	0	0	0	0	0
	Waste PP	GW	0	0	0	0	0	0	0	0
	Biogas PP	GW	0	0	0	0	0	0	0	0
	Hard coal PP	GW	2	2	2	2	2	2	1	1
	Internal combustion generator (ICG)	GW	0	0	0	0	1	1	1	2
	Multifuel Internal combustion generator	GW	0	0	2	2	2	2	2	2
	Nuclear PP	GW	0	0	0	0	0	0	0	0
	Battery storage	GWh	0	0	2	15	42	61	97	157
	PHES storage	GWh	0	0	0	0	0	0	0	0
	Thermal Energy Storage (TES)	GWh	0	0	0	0	0	0	0	0
	Adiabatic Compressed Air Energy Storage (A-CAES)	GWh	0	0	0	0	0	0	0	0
	Power-to-Gas	GW_{el}	0	0	0	0	2	2	2	2
	Gas storage	GWh	52	53	53	309	3405	3805	3807	3811
**Bihar**	PV prosumers	GW	0	0	2	5	9	13	19	25
	PV fixed tilted	GW	0	0	0	0	8	12	14	26
	PV single-axis tracking	GW	0	0	0	3	3	3	7	7
	Concentrating Solar Thermal Power (CSP)	GW	0	0	0	0	0	0	0	0
	Wind energy	GW	0	0	3	3	3	3	3	3
	Hydropower Run-of-River	GW	0	0	0	0	0	0	0	0
	Hydropower reservoir (dam)	GW	0	0	0	0	0	0	0	0
	Geothermal power	GW	0	0	1	2	2	2	2	2
	Combined Cycle Gas Turbine (CCGT)	GW	0	0	0	0	0	0	0	0
	Open Cycle Gas Turbine (OCGT)	GW	0	0	0	0	0	0	0	0
	Heavy Duty Open Cycle Gas Turbine (OCGT HD)	GW	0	0	0	0	0	0	0	0
	Biomass PP	GW	0	0	1	1	2	2	1	1
	Waste PP	GW	0	0	0	0	0	0	0	0
	Biogas PP	GW	0	0	0	0	0	0	0	0
	Hard coal PP	GW	3	6	6	6	6	6	5	5
	Internal combustion generator (ICG)	GW	0	0	0	1	1	1	2	2
	Multifuel Internal combustion generator	GW	0	0	0	1	1	1	1	1
	Nuclear PP	GW	0	0	0	0	0	0	0	0

	Battery storage	GWh	0	0	3	21	42	59	99	158
	PHES storage	GWh	0	0	0	0	0	0	0	0
	Thermal Energy Storage (TES)	GWh	0	0	0	0	0	0	0	0
	Adiabatic Compressed Air Energy Storage (A-CAES)	GWh	0	0	0	0	0	0	0	0
	Power-to-Gas	GW_{el}	0	0	0	0	0	0	0	0
	Gas storage	GWh	52	53	53	127	828	930	930	932
**Northeastern states**	PV prosumers	GW	0	0	1	3	6	9	12	16
	PV fixed tilted	GW	0	0	0	0	1	4	4	11
	PV single-axis tracking	GW	0	0	0	0	0	0	4	4
	Concentrating Solar Thermal Power (CSP)	GW	0	0	0	0	0	0	0	0
	Wind energy	GW	0	0	1	2	2	2	2	2
	Hydropower Run-of-River	GW	1	1	1	1	1	1	1	1
	Hydropower reservoir (dam)	GW	1	1	1	1	1	1	1	1
	Geothermal power	GW	0	0	1	1	1	1	1	1
	Combined Cycle Gas Turbine (CCGT)	GW	1	1	1	1	1	1	1	1
	Open Cycle Gas Turbine (OCGT)	GW	0	0	0	0	0	0	0	0
	Heavy Duty Open Cycle Gas Turbine (OCGT HD)	GW	0	0	0	0	0	0	0	0
	Biomass PP	GW	0	0	0	1	1	1	1	1
	Waste PP	GW	0	0	0	0	0	0	0	0
	Biogas PP	GW	0	0	0	0	0	0	0	0
	Hard coal PP	GW	0	1	1	1	1	1	1	1
	Internal combustion generator (ICG)	GW	0	0	0	0	0	1	1	1
	Multifuel Internal combustion generator	GW	0	0	0	0	0	0	0	0
	Nuclear PP	GW	0	0	0	0	0	0	0	0
	Battery storage	GWh	0	0	2	8	17	28	42	62
	PHES storage	GWh	0	0	0	0	0	0	0	0
	Thermal Energy Storage (TES)	GWh	0	0	0	0	0	0	0	0
	Adiabatic Compressed Air Energy Storage (A-CAES)	GWh	0	0	0	0	0	0	0	0
	Power-to-Gas	GW_{el}	0	0	0	0	0	0	1	1
	Gas storage	GWh	65	65	65	65	261	339	1222	1222

Supplementary Table 6: Transmission capacities of all interconnections in 10 year intervals from 2020 to 2050.

Transmission Line		Units	2020	2030	2040	2050
Jammu-Kashmir	Himachal Pradesh	MW	370	390	1701	1703
Jammu-Kashmir	Punjab & Chandigarh	MW	161	2361	5073	5073
Himachal Pradesh	Uttarakhand	MW	1569	7046	8452	8812
Himachal Pradesh	Punjab & Chandigarh	MW	8902	24679	35661	41024
Uttarakhand	Uttar Pradesh	MW	1702	6628	7395	7395
Punjab & Chandigarh	Haryana	MW	6131	22951	33698	33701
Haryana	Delhi	MW	1390	7905	10828	17504
Haryana	Rajasthan	MW	2461	16243	24389	28738
Delhi	Uttar Pradesh	MW	16	884	1737	1738
Rajasthan	Uttar Pradesh	MW	2	6843	8707	17265
Rajasthan	Madhya Pradesh	MW	0	0	0	0
Rajasthan	Gujarat, Daman & Dadra	MW	0	0	0	0
Uttar Pradesh	Bihar	MW	0	0	0	0
Madhya Pradesh	Gujarat, Daman & Dadra	MW	421	8265	8590	11490
Madhya Pradesh	Maharashtra & Goa	MW	6749	6750	7428	15861
Madhya Pradesh	Chhattisgarh	MW	5286	11532	13606	17857
Gujarat, Daman & Dadra	Maharashtra & Goa	MW	400	8845	12710	19219
Maharashtra & Goa	Karnataka	MW	0	0	0	0
Chhattisgarh	Telangana	MW	0	0	0	0
Chhattisgarh	Odisha	MW	0	0	0	0
Chhattisgarh	Jharkhand	MW	0	0	0	0
Kerala	Tamil Nadu & Puducherry	MW	3	140	391	429
Kerala	Karnataka	MW	700	1989	2835	4205
Tamil Nadu & Puducherry	Karnataka	MW	641	8659	14655	27587
Tamil Nadu & Puducherry	Andhra Pradesh	MW	806	2258	5448	5918
Karnataka	Telangana	MW	53	3763	6536	7112
Andhra Pradesh	Telangana	MW	1840	3864	3868	5971
Andhra Pradesh	Odisha	MW	0	0	0	0
Odisha	West Bengal & Sikkim	MW	6	3812	5422	7476
Odisha	Jharkhand	MW	4482	4483	5049	5050
West Bengal & Sikkim	Jharkhand	MW	120	2737	3414	4856
Jharkhand	Bihar	MW	3038	8003	10270	10455
Bihar	Northeastern states	MW	1153	1300	1403	2214

Supplementary Table 7: Electricity transfer across all the interconnections in 10 year intervals from 2020 to 2050.

Transmission Line		Units	2020	2030	2040	2050
Jammu-Kashmir	Himachal Pradesh	GWh	727	1104	3047	3503
Jammu-Kashmir	Punjab & Chandigarh	GWh	250	10520	17697	14374
Himachal Pradesh	Uttarakhand	GWh	914	30014	29195	24573
Himachal Pradesh	Punjab & Chandigarh	GWh	31371	113282	160629	213050
Uttarakhand	Uttar Pradesh	GWh	1832	29679	25786	19899
Punjab & Chandigarh	Haryana	GWh	12563	102885	136046	115823
Haryana	Delhi	GWh	1896	51876	65849	120916
Haryana	Rajasthan	GWh	5069	62004	99275	111202
Delhi	Uttar Pradesh	GWh	7	3274	5514	5821
Rajasthan	Uttar Pradesh	GWh	2	27514	35247	66306
Rajasthan	Madhya Pradesh	GWh	0	0	0	0
Rajasthan	Gujarat, Daman & Dadra	GWh	0	0	0	0
Uttar Pradesh	Bihar	GWh	0	0	0	0
Madhya Pradesh	Gujarat, Daman & Dadra	GWh	875	23284	33363	38631
Madhya Pradesh	Maharashtra & Goa	GWh	152	16762	38491	87451
Madhya Pradesh	Chhattisgarh	GWh	840	34262	45902	59236
Gujarat, Daman & Dadra	Maharashtra & Goa	GWh	1255	21958	43934	76793
Maharashtra & Goa	Karnataka	GWh	0	0	0	0
Chhattisgarh	Telangana	GWh	0	0	0	0
Chhattisgarh	Odisha	GWh	0	0	0	0
Chhattisgarh	Jharkhand	GWh	0	0	0	0
Kerala	Tamil Nadu & Puducherry	GWh	10	681	1385	1247
Kerala	Karnataka	GWh	4735	9357	11200	17703
Tamil Nadu & Puducherry	Karnataka	GWh	1057	37051	64242	128104
Tamil Nadu & Puducherry	Andhra Pradesh	GWh	882	6755	18191	21863
Karnataka	Telangana	GWh	129	12528	23579	30620
Andhra Pradesh	Telangana	GWh	2384	10973	11640	21175
Andhra Pradesh	Odisha	GWh	0	0	0	0
Odisha	West Bengal & Sikkim	GWh	6	11248	17691	26477
Odisha	Jharkhand	GWh	1189	13864	17279	14754
West Bengal & Sikkim	Jharkhand	GWh	467	9077	12237	18934
Jharkhand	Bihar	GWh	3498	23893	30739	28648
Bihar	Northeastern states	GWh	7652	7753	7572	10480

Supplementary Table 8: Utilisation of all transmission lines in 10 year intervals from 2020 to 2050.

Transmission Line		Units	2020	2030	2040	2050
Jammu-Kashmir	Himachal Pradesh	%	22	32	20	23
Jammu-Kashmir	Punjab & Chandigarh	%	18	51	40	32
Himachal Pradesh	Uttarakhand	%	7	49	39	32
Himachal Pradesh	Punjab & Chandigarh	%	40	52	51	59
Uttarakhand	Uttar Pradesh	%	12	51	40	31
Punjab & Chandigarh	Haryana	%	23	51	46	39
Haryana	Delhi	%	16	75	69	79
Haryana	Rajasthan	%	24	44	46	44
Delhi	Uttar Pradesh	%	5	42	36	38
Rajasthan	Uttar Pradesh	%	13	46	46	44
Rajasthan	Madhya Pradesh	%	0	0	0	0
Rajasthan	Gujarat, Daman & Dadra	%	0	0	0	0
Uttar Pradesh	Bihar	%	0	0	0	0
Madhya Pradesh	Gujarat, Daman & Dadra	%	24	32	44	38
Madhya Pradesh	Maharashtra & Goa	%	0	28	59	63
Madhya Pradesh	Chhattisgarh	%	2	34	39	38
Gujarat, Daman & Dadra	Maharashtra & Goa	%	36	28	39	46
Maharashtra & Goa	Karnataka	%	0	0	0	0
Chhattisgarh	Telangana	%	0	0	0	0
Chhattisgarh	Odisha	%	0	0	0	0
Chhattisgarh	Jharkhand	%	0	0	0	0
Kerala	Tamil Nadu & Puducherry	%	33	56	40	33
Kerala	Karnataka	%	77	54	45	48
Tamil Nadu & Puducherry	Karnataka	%	19	49	50	53
Tamil Nadu & Puducherry	Andhra Pradesh	%	12	34	38	42
Karnataka	Telangana	%	28	38	41	49
Andhra Pradesh	Telangana	%	15	32	34	40
Andhra Pradesh	Odisha	%	0	0	0	0
Odisha	West Bengal & Sikkim	%	12	34	37	40
Odisha	Jharkhand	%	3	35	39	33
West Bengal & Sikkim	Jharkhand	%	44	38	41	45
Jharkhand	Bihar	%	13	34	34	31
Bihar	Northeastern states	%	76	68	62	54

Supplementary Table 9: Generation/Output of different energy technologies for each state/region from 2020 to 2050.

	Technologies	Units	2015	2020	2025	2030	2035	2040	2045	2050
**Jammu & Kashmir**	PV prosumers	GWh	0	164	846	4307	7869	11773	15697	20465
	PV fixed tilted	GWh	0	16	16	16	22	26	31	7597
	PV single-axis tracking	GWh	0	0	6924	6924	6926	10965	18755	18755
	Concentrating Solar Thermal Power (CSP)	GWh	0	0	0	0	0	0	0	0
	Wind energy	GWh	0	0	1176	7919	25546	25549	25549	25549
	Hydropower Run-of-River	GWh	8125	8186	8186	8186	8187	8187	8187	8187
	Hydropower reservoir (dam)	GWh	6019	9188	13782	13782	13782	13782	13782	13782
	Geothermal power	GWh	0	0	0	0	0	0	0	0
	Power-to-Gas	$GWhe_l$	0	0	0	542	429	455	429	408
	Combined Cycle Gas Turbine (CCGT)	GWh	675	647	160	137	93	108	100	81
	Open Cycle Gas Turbine (OCGT)	GWh	452	43	92	53	1	0	0	0
	Heavy Duty Open Cycle Gas Turbine (OCGT HD)	GWh	0	0	1	1	1	1	1	1
	Biomass PP	GWh	0	0	234	359	363	364	366	368
	Waste-to-Power	GWh	0	358	716	1109	1137	1140	1140	1140
	Biogas PP	GWh	0	45	91	144	151	154	156	158
	Hard Coal PP	GWh	2602	2243	2	1	1	0	0	0
	Internal Combustion Generator (ICG)	GWh	0	0	0	0	0	0	0	0
	Multi-fuel Internal Combustion Generator (ICG)	GWh	0	0	48	141	185	193	182	169
	Nuclear PP	GWh	0	0	0	0	0	0	0	0
	Battery storage output	TWh	0	0	0	2	5	8	13	18
	Pumped hydro energy storage (PHES) output	TWh	0	0	0	0	0	0	0	0
	Thermal Energy Storage (TES) output	TWh	0	0	0	0	0	0	0	0
	Adiabatic Compressed Air Energy Storage (A-CAES) output	TWh	0	0	0	0	0	0	0	0
	Gas storage output	TWh	0	0	0	1	0	0	0	0
**Himachal Pradesh**	PV prosumers	GWh	0	109	736	3096	5147	7699	10494	13770
	PV fixed tilted	GWh	0	31	31	31	48	98	115	50168
	PV single-axis tracking	GWh	0	0	55194	55194	55196	85354	114558	114558
	Concentrating Solar Thermal Power (CSP)	GWh	0	0	0	0	0	0	0	0
	Wind energy	GWh	0	0	17293	37768	58906	58906	58906	58906
	Hydropower Run-of-River	GWh	1629	1854	1854	1854	1854	1854	1854	1854
	Hydropower reservoir (dam)	GWh	34590	39350	59026	59026	59026	59026	59026	59026
	Geothermal power	GWh	0	0	0	0	0	0	0	0
	Power-to-Gas	$GWhe_l$	0	0	0	4	17	20	23	22
	Combined Cycle Gas Turbine (CCGT)	GWh	137	53	33	37	41	43	41	33
	Open Cycle Gas Turbine (OCGT)	GWh	0	0	0	1	1	0	0	0

	Heavy Duty Open Cycle Gas Turbine (OCGT HD)	GWh	0	0	1	2	2	2	2	3
	Biomass PP	GWh	1316	1592	1848	2143	2167	2173	2185	2196
	Waste-to-Power	GWh	0	95	191	295	303	304	304	304
	Biogas PP	GWh	0	28	57	90	94	96	98	104
	Hard Coal PP	GWh	754	14	2	1	1	0	0	0
	Internal Combustion Generator (ICG)	GWh	1	0	0	0	0	0	0	0
	Multi-fuel Internal Combustion Generator (ICG)	GWh	0	0	3	10	13	12	15	15
	Nuclear PP	GWh	0	0	0	0	0	0	0	0
	Battery storage output	TWh	0	0	0	6	14	29	46	74
	Pumped hydro energy storage (PHES) output	TWh	0	0	0	0	0	0	0	0
	Thermal Energy Storage (TES) output	TWh	0	0	0	0	0	0	0	0
	Adiabatic Compressed Air Energy Storage (A-CAES) output	TWh	0	0	0	0	0	0	0	0
	Gas storage output	TWh	0	0	0	0	0	0	0	0
**Uttarakhand**	PV prosumers	GWh	0	162	762	3930	7512	11332	15212	20479
	PV fixed tilted	GWh	9	428	428	432	4397	4425	4439	16757
	PV single-axis tracking	GWh	0	0	4396	6242	6257	10742	17080	17080
	Concentrating Solar Thermal Power (CSP)	GWh	0	0	0	0	0	0	0	0
	Wind energy	GWh	0	0	0	0	1	1	1	1
	Hydropower Run-of-River	GWh	7884	9078	9078	9078	9078	9078	9078	9078
	Hydropower reservoir (dam)	GWh	4788	4788	7182	7182	7182	7182	7182	7182
	Geothermal power	GWh	0	0	0	0	0	0	0	0
	Power-to-Gas	GWh_{el}	0	0	0	118	92	111	105	111
	Combined Cycle Gas Turbine (CCGT)	GWh	154	1965	273	268	66	74	74	76
	Open Cycle Gas Turbine (OCGT)	GWh	0	0	0	0	0	0	0	0
	Heavy Duty Open Cycle Gas Turbine (OCGT HD)	GWh	0	0	1	1	1	1	1	0
	Biomass PP	GWh	1316	1426	1517	1633	1651	1656	1665	1674
	Waste-to-Power	GWh	0	145	289	448	459	461	461	461
	Biogas PP	GWh	0	32	63	100	105	107	108	109
	Hard Coal PP	GWh	2292	1534	2	1	1	0	0	0
	Internal Combustion Generator (ICG)	GWh	0	0	0	0	0	0	0	0
	Multi-fuel Internal Combustion Generator (ICG)	GWh	0	0	4	30	27	33	28	24
	Nuclear PP	GWh	0	0	0	0	0	0	0	0
	Battery storage output	TWh	0	0	0	3	5	8	13	21
	Pumped hydro energy storage (PHES) output	TWh	0	0	0	0	0	0	0	0
	Thermal Energy Storage (TES) output	TWh	0	0	0	0	0	0	0	0
	Adiabatic Compressed Air Energy Storage (A-CAES) output	TWh	0	0	0	0	0	0	0	0
	Gas storage output	TWh	0	0	0	0	0	0	0	0
**Punjab &**	PV prosumers	GWh	0	732	6994	18336	30576	46396	62312	83753
	PV fixed tilted	GWh	328	1360	1360	1360	1361	1361	1361	1036
	PV single-axis tracking	GWh	0	0	0	0	0	0	0	1

	Concentrating Solar Thermal Power (CSP)	GWh	0	0	0	0	0	0	0	0
	Wind energy	GWh	0	0	2469	42397	53480	53480	53480	53480
	Hydropower Run-of-River	GWh	438	438	438	438	438	438	438	438
	Hydropower reservoir (dam)	GWh	4143	4216	6324	6324	6324	6324	6324	6324
	Geothermal power	GWh	0	0	0	0	0	0	0	0
	Power-to-Gas	GWhe$_{l}$	0	0	0	4	18	24	28	31
	Combined Cycle Gas Turbine (CCGT)	GWh	1013	357	226	206	156	193	176	175
	Open Cycle Gas Turbine (OCGT)	GWh	0	0	0	1	1	0	0	0
	Heavy Duty Open Cycle Gas Turbine (OCGT HD)	GWh	0	0	1	1	2	1	1	1
	Biomass PP	GWh	1316	4161	21777	32763	33127	33218	33400	33582
	Waste-to-Power	GWh	0	12	25	39	40	40	40	40
	Biogas PP	GWh	0	389	779	1232	1294	1321	1339	1351
	Hard Coal PP	GWh	38100	38701	3	1	1	0	0	0
	Internal Combustion Generator (ICG)	GWh	0	0	0	0	0	0	0	0
	Multi-fuel Internal Combustion Generator (ICG)	GWh	0	0	234	514	586	550	568	568
	Nuclear PP	GWh	0	0	0	0	0	0	0	0
	Battery storage output	TWh	0	0	3	8	12	19	24	32
	Pumped hydro energy storage (PHES) output	TWh	0	0	0	0	0	0	0	0
	Thermal Energy Storage (TES) output	TWh	0	0	0	0	0	0	0	0
	Adiabatic Compressed Air Energy Storage (A-CAES) output	TWh	0	0	0	0	0	0	0	0
	Gas storage output	TWh	0	1	1	2	2	2	2	2
**Haryana**	PV prosumers	GWh	0	659	6628	16628	28027	42031	57290	79587
	PV fixed tilted	GWh	21	213	213	213	217	10625	54786	128754
	PV single-axis tracking	GWh	0	0	0	0	9	10	10	10
	Concentrating Solar Thermal Power (CSP)	GWh	0	0	0	0	0	0	0	0
	Wind energy	GWh	0	0	37528	43006	46844	46844	46844	46844
	Hydropower Run-of-River	GWh	0	0	0	0	0	0	0	0
	Hydropower reservoir (dam)	GWh	343	343	514	514	514	514	514	514
	Geothermal power	GWh	0	0	0	0	0	0	0	0
	Power-to-Gas	GWhe$_{l}$	0	0	0	3305	3230	3247	3023	3197
	Combined Cycle Gas Turbine (CCGT)	GWh	1635	1493	360	333	1	1	2	1
	Open Cycle Gas Turbine (OCGT)	GWh	637	65	130	130	1	0	0	0
	Heavy Duty Open Cycle Gas Turbine (OCGT HD)	GWh	0	0	1	1	1	0	0	0
	Biomass PP	GWh	1316	4161	10477	15401	15572	15614	15700	15786
	Waste-to-Power	GWh	0	12	25	38	39	39	39	39
	Biogas PP	GWh	0	271	543	858	901	920	930	937
	Hard Coal PP	GWh	47595	69476	502	1	2	0	0	0
	Internal Combustion Generator (ICG)	GWh	97	0	9	0	0	0	0	0
	Multi-fuel Internal Combustion Generator (ICG)	GWh	0	0	2205	2205	2189	2238	2057	2046
	Nuclear PP	GWh	0	0	0	0	0	0	0	0

	Battery storage output	TWh	0	0	2	13	16	24	45	80
	Pumped hydro energy storage (PHES) output	TWh	0	0	0	0	0	0	0	0
	Thermal Energy Storage (TES) output	TWh	0	0	0	0	0	0	0	0
	Adiabatic Compressed Air Energy Storage (A-CAES) output	TWh	0	0	0	0	0	0	0	0
	Gas storage output	TWh	0	0	1	3	3	3	3	3
**Delhi**	PV prosumers	GWh	0	507	4481	10967	17869	22184	25845	28288
	PV fixed tilted	GWh	11	15	15	10744	10870	10871	10871	10871
	PV single-axis tracking	GWh	0	0	0	8	5040	11920	11921	11921
	Concentrating Solar Thermal Power (CSP)	GWh	0	0	0	0	0	0	0	0
	Wind energy	GWh	0	0	1358	1443	1571	1571	1571	1571
	Hydropower Run-of-River	GWh	0	0	0	0	0	0	0	0
	Hydropower reservoir (dam)	GWh	0	0	0	0	0	0	0	0
	Geothermal power	GWh	0	0	0	0	0	0	0	0
	Power-to-Gas	GWhe$_l$	0	0	1	9045	8619	8756	8215	8542
	Combined Cycle Gas Turbine (CCGT)	GWh	5463	9026	1112	969	3	3	6	3
	Open Cycle Gas Turbine (OCGT)	GWh	28	3	6	0	0	0	0	0
	Heavy Duty Open Cycle Gas Turbine (OCGT HD)	GWh	0	0	1	1	1	1	1	0
	Biomass PP	GWh	0	0	0	0	0	0	0	0
	Waste-to-Power	GWh	0	0	0	0	0	0	0	0
	Biogas PP	GWh	0	4	7	11	12	12	12	12
	Hard Coal PP	GWh	31170	36594	11883	5	19	0	0	0
	Internal Combustion Generator (ICG)	GWh	14	0	1	0	0	0	0	0
	Multi-fuel Internal Combustion Generator (ICG)	GWh	0	0	5142	5142	4497	4716	4258	4179
	Nuclear PP	GWh	0	0	0	0	0	0	0	0
	Battery storage output	TWh	0	0	2	9	13	18	22	24
	Pumped hydro energy storage (PHES) output	TWh	0	0	0	0	0	0	0	0
	Thermal Energy Storage (TES) output	TWh	0	0	0	0	0	0	0	0
	Adiabatic Compressed Air Energy Storage (A-CAES) output	TWh	0	0	0	0	0	0	0	0
	Gas storage output	TWh	0	0	0	6	5	6	5	5
	PV prosumers	GWh	0	970	9484	23398	37661	55837	73430	98167
	PV fixed tilted	GWh	1708	7565	7565	7565	33216	128796	170318	359186
	PV single-axis tracking	GWh	0	0	11253	63253	83725	90922	193564	193564
	Concentrating Solar Thermal Power (CSP)	GWh	0	0	0	0	0	0	0	0
	Wind energy	GWh	8493	11036	57159	63727	80608	78000	75392	72268
	Hydropower Run-of-River	GWh	49	49	49	49	49	49	49	49
	Hydropower reservoir (dam)	GWh	3168	3168	3168	3168	4741	4741	4741	4741
	Geothermal power	GWh	0	0	0	0	0	0	0	0
	Power-to-Gas	GWhe$_l$	0	0	0	12279	12505	13821	34652	39337
	Combined Cycle Gas Turbine (CCGT)	GWh	194	310	39	39	1	1	0	0
	Open Cycle Gas Turbine (OCGT)	GWh	3555	468	723	583	1	0	0	0

Rajasthan	Heavy Duty Open Cycle Gas Turbine (OCGT HD)	GWh	0	0	1	1	1	0	0	0
	Biomass PP	GWh	1316	4161	8219	11931	12064	12097	12163	12230
	Waste-to-Power	GWh	0	298	596	923	946	949	949	949
	Biogas PP	GWh	0	275	549	869	912	931	941	949
	Hard Coal PP	GWh	50780	66082	18827	111	27	0	0	0
	Internal Combustion Generator (ICG)	GWh	409	0	15	0	0	0	0	0
	Multi-fuel Internal Combustion Generator (ICG)	GWh	0	0	8005	8005	6840	7662	17120	17550
	Nuclear PP	GWh	6016	8042	8042	6552	6552	6552	6552	3276
	Battery storage output	TWh	0	0	5	23	57	107	192	301
	Pumped hydro energy storage (PHES) output	TWh	0	0	0	0	0	0	0	0
	Thermal Energy Storage (TES) output	TWh	0	0	0	0	0	0	0	0
	Adiabatic Compressed Air Energy Storage (A-CAES) output	TWh	0	0	0	0	0	0	0	0
	Gas storage output	TWh	0	0	1	9	9	10	23	26
Uttar Pradesh	PV prosumers	GWh	0	1396	13466	34670	58746	87860	118658	156461
	PV fixed tilted	GWh	117	1474	1474	52091	84254	146976	217004	305356
	PV single-axis tracking	GWh	0	0	26226	56728	56728	56728	56728	56729
	Concentrating Solar Thermal Power (CSP)	GWh	0	0	0	0	0	0	0	0
	Wind energy	GWh	0	0	50272	65149	70963	70964	70964	70965
	Hydropower Run-of-River	GWh	87	104	104	104	104	104	104	104
	Hydropower reservoir (dam)	GWh	994	994	994	994	1490	1490	1490	1490
	Geothermal power	GWh	0	0	0	0	0	0	0	0
	Power-to-Gas	GWh$_{el}$	0	0	0	21220	20475	20277	19645	19739
	Combined Cycle Gas Turbine (CCGT)	GWh	3829	4838	779	779	1	0	0	0
	Open Cycle Gas Turbine (OCGT)	GWh	0	0	0	0	0	0	0	0
	Heavy Duty Open Cycle Gas Turbine (OCGT HD)	GWh	0	0	1	1	1	0	0	0
	Biomass PP	GWh	1316	4161	12495	18501	18707	18758	18861	18964
	Waste-to-Power	GWh	0	174	347	538	552	553	553	553
	Biogas PP	GWh	0	412	824	1303	1368	1397	1411	1422
	Hard Coal PP	GWh	99076	129208	45643	64	9	0	0	0
	Internal Combustion Generator (ICG)	GWh	1802	0	165	165	0	0	0	0
	Multi-fuel Internal Combustion Generator (ICG)	GWh	0	0	14622	14622	11375	11451	10759	10024
	Nuclear PP	GWh	2243	3276	3276	3276	3276	0	0	0
	Battery storage output	TWh	0	0	5	43	71	118	167	226
	Pumped hydro energy storage (PHES) output	TWh	0	0	0	0	0	0	0	0
	Thermal Energy Storage (TES) output	TWh	0	0	0	0	0	0	0	0
	Adiabatic Compressed Air Energy Storage (A-CAES) output	TWh	0	0	0	0	0	0	0	0
	Gas storage output	TWh	0	1	1	15	15	14	14	14
Madhya [illegible]	PV prosumers	GWh	0	710	7765	19308	33918	54515	79469	109978
	PV fixed tilted	GWh	902	3503	3503	3503	17814	22744	22744	75309
	PV single-axis tracking	GWh	0	0	0	9401	9401	9401	9917	9918

	Concentrating Solar Thermal Power (CSP)	GWh	0	0	0	0	0	0	0	0
	Wind energy	GWh	2259	6468	40757	83840	90674	91027	107242	102071
	Hydropower Run-of-River	GWh	721	721	721	721	721	721	721	721
	Hydropower reservoir (dam)	GWh	4113	4113	4113	4113	6142	6143	6143	6169
	Geothermal power	GWh	0	0	0	0	0	0	0	0
	Power-to-Gas	GWhe$_l$	0	0	0	303	423	848	1045	4993
	Combined Cycle Gas Turbine (CCGT)	GWh	791	1747	188	161	6	18	31	33
	Open Cycle Gas Turbine (OCGT)	GWh	0	0	0	1	0	2	1	1
	Heavy Duty Open Cycle Gas Turbine (OCGT HD)	GWh	0	0	0	4	1	6	3	3
	Biomass PP	GWh	1047	4376	9454	13892	14085	14201	14279	14356
	Waste-to-Power	GWh	0	580	1161	1804	1850	1850	1850	1850
	Biogas PP	GWh	0	258	515	813	856	882	906	921
	Hard Coal PP	GWh	86329	69308	44304	6116	80	72	154	0
	Internal Combustion Generator (ICG)	GWh	235	230	8	0	0	0	0	0
	Multi-fuel Internal Combustion Generator (ICG)	GWh	0	0	106	532	693	853	908	2518
	Nuclear PP	GWh	0	0	0	0	0	0	0	0
	Battery storage output	TWh	0	0	3	12	22	32	42	60
	Pumped hydro energy storage (PHES) output	TWh	0	0	0	0	0	0	0	0
	Thermal Energy Storage (TES) output	TWh	0	0	0	0	0	0	0	0
	Adiabatic Compressed Air Energy Storage (A-CAES) output	TWh	0	0	0	0	0	0	0	0
	Gas storage output	TWh	0	0	1	1	1	2	2	4
**Gujarat, Daman & Dadra**	PV prosumers	GWh	0	1299	7227	33444	62907	94972	125359	164273
	PV fixed tilted	GWh	1646	3884	3884	3884	21373	21380	21381	19734
	PV single-axis tracking	GWh	0	0	5386	52271	52273	85874	114694	206260
	Concentrating Solar Thermal Power (CSP)	GWh	0	0	0	0	0	0	0	0
	Wind energy	GWh	9356	18889	78151	132475	158006	169083	208598	208344
	Hydropower Run-of-River	GWh	5	228	228	228	228	228	228	228
	Hydropower reservoir (dam)	GWh	530	531	531	531	531	531	531	531
	Geothermal power	GWh	0	0	274	274	274	274	274	274
	Power-to-Gas	GWhe$_l$	0	0	0	1879	5413	6572	12133	18663
	Combined Cycle Gas Turbine (CCGT)	GWh	17164	31719	3506	3491	110	170	902	1199
	Open Cycle Gas Turbine (OCGT)	GWh	3810	443	775	767	0	2	1	286
	Heavy Duty Open Cycle Gas Turbine (OCGT HD)	GWh	0	0	0	3	1	5	3	3
	Biomass PP	GWh	1047	4376	8387	12262	12433	12535	12603	12672
	Waste-to-Power	GWh	0	390	781	1213	1244	1244	1244	1244
	Biogas PP	GWh	0	226	451	711	749	772	790	807
	Hard Coal PP	GWh	105828	104406	96168	22647	1539	119	6	0
	Internal Combustion Generator (ICG)	GWh	1651	2317	33	5	0	0	0	0
	Multi-fuel Internal Combustion Generator (ICG)	GWh	0	0	667	1566	2868	3161	4549	6307
	Nuclear PP	GWh	2243	3276	3276	3276	3276	1638	0	0

	Battery storage output	TWh	0	0	2	31	55	80	97	139
	Pumped hydro energy storage (PHES) output	TWh	0	0	0	0	0	0	0	0
	Thermal Energy Storage (TES) output	TWh	0	0	0	0	0	0	0	0
	Adiabatic Compressed Air Energy Storage (A-CAES) output	TWh	0	0	0	0	0	0	0	0
	Gas storage output	TWh	0	0	1	2	4	5	9	13
**Maharashtra & Goa**	PV prosumers	GWh	0	2201	21156	56037	93321	143531	197743	266862
	PV fixed tilted	GWh	577	2296	2296	2296	82348	82393	82394	81818
	PV single-axis tracking	GWh	0	0	3897	78468	78468	105136	130858	130858
	Concentrating Solar Thermal Power (CSP)	GWh	0	0	0	0	0	0	0	0
	Wind energy	GWh	11411	12834	62211	101145	112575	134362	179568	265606
	Hydropower Run-of-River	GWh	925	945	945	945	945	945	945	945
	Hydropower reservoir (dam)	GWh	7123	7123	7123	10630	10685	10685	10685	10685
	Geothermal power	GWh	0	0	0	0	0	0	0	0
	Power-to-Gas	$GWhe_l$	0	0	0	695	3700	4245	9605	15581
	Combined Cycle Gas Turbine (CCGT)	GWh	7980	18846	1846	1827	56	61	177	352
	Open Cycle Gas Turbine (OCGT)	GWh	0	0	0	1	0	2	0	1
	Heavy Duty Open Cycle Gas Turbine (OCGT HD)	GWh	0	0	0	4	1	5	2	3
	Biomass PP	GWh	1047	4376	13334	19820	20096	20262	20372	20482
	Waste-to-Power	GWh	0	594	1188	1846	1893	1893	1893	1893
	Biogas PP	GWh	0	468	935	1474	1552	1599	1639	1672
	Hard Coal PP	GWh	109540	162854	139686	38135	3982	414	0	0
	Internal Combustion Generator (ICG)	GWh	264	58	4	4	0	0	0	0
	Multi-fuel Internal Combustion Generator (ICG)	GWh	0	0	493	1801	2615	2773	4943	7001
	Nuclear PP	GWh	7138	9233	8042	8042	8042	8042	8042	8042
	Battery storage output	TWh	0	0	8	47	94	132	166	186
	Pumped hydro energy storage (PHES) output	TWh	0	0	2	1	1	1	1	1
	Thermal Energy Storage (TES) output	TWh	0	0	0	0	0	0	0	0
	Adiabatic Compressed Air Energy Storage (A-CAES) output	TWh	0	0	0	0	0	0	0	0
	Gas storage output	TWh	0	1	1	2	4	5	8	12
**Chhattisgarh**	PV prosumers	GWh	0	247	1287	7266	12580	20817	30166	42945
	PV fixed tilted	GWh	16	334	334	334	25986	26016	26016	26001
	PV single-axis tracking	GWh	0	0	0	8816	8816	14771	27416	36796
	Concentrating Solar Thermal Power (CSP)	GWh	0	0	0	0	0	0	0	0
	Wind energy	GWh	0	0	9052	48515	52846	52848	63468	63468
	Hydropower Run-of-River	GWh	46	107	107	107	107	107	107	107
	Hydropower reservoir (dam)	GWh	305	305	305	453	458	458	458	458
	Geothermal power	GWh	0	0	0	0	0	0	0	0
	Power-to-Gas	$GWhe_l$	0	0	0	375	595	990	1697	4237
	Combined Cycle Gas Turbine (CCGT)	GWh	0	0	0	1	1	5	1	1
	Open Cycle Gas Turbine (OCGT)	GWh	33	10	10	10	0	3	1	32

	Heavy Duty Open Cycle Gas Turbine (OCGT HD)	GWh	0	0	0	4	1	6	3	2
	Biomass PP	GWh	1047	1817	2570	3373	3420	3448	3467	3486
	Waste-to-Power	GWh	0	429	858	1333	1366	1366	1366	1366
	Biogas PP	GWh	0	163	238	376	395	409	420	425
	Hard Coal PP	GWh	100716	37011	27168	3239	163	159	363	0
	Internal Combustion Generator (ICG)	GWh	0	0	0	0	0	0	0	0
	Multi-fuel Internal Combustion Generator (ICG)	GWh	0	0	156	476	510	653	945	1911
	Nuclear PP	GWh	0	0	0	0	0	0	0	0
	Battery storage output	TWh	0	0	0	13	21	27	34	45
	Pumped hydro energy storage (PHES) output	TWh	0	0	0	0	0	0	0	0
	Thermal Energy Storage (TES) output	TWh	0	0	0	0	0	0	0	0
	Adiabatic Compressed Air Energy Storage (A-CAES) output	TWh	0	0	0	0	0	0	0	0
	Gas storage output	TWh	0	0	0	1	1	1	2	3
**Kerala**	PV prosumers	GWh	0	279	3036	8192	14201	21884	29981	41546
	PV fixed tilted	GWh	0	158	158	158	158	158	158	176
	PV single-axis tracking	GWh	0	0	8802	17716	17718	24571	39805	43970
	Concentrating Solar Thermal Power (CSP)	GWh	0	0	0	0	0	0	0	0
	Wind energy	GWh	90	160	9248	10910	11858	15891	15863	29555
	Hydropower Run-of-River	GWh	1536	1664	1664	1664	1664	1664	1664	1664
	Hydropower reservoir (dam)	GWh	6545	6545	9790	9818	9818	9818	9818	9818
	Geothermal power	GWh	0	0	0	0	0	0	0	0
	Power-to-Gas	$GWhe_l$	0	0	6	689	729	753	821	995
	Combined Cycle Gas Turbine (CCGT)	GWh	1376	346	280	280	8	6	2	9
	Open Cycle Gas Turbine (OCGT)	GWh	0	0	2	2	0	1	0	9
	Heavy Duty Open Cycle Gas Turbine (OCGT HD)	GWh	0	0	6	6	1	2	3	17
	Biomass PP	GWh	1696	4140	6565	9139	9293	9293	9395	9549
	Waste-to-Power	GWh	0	68	135	209	214	215	215	215
	Biogas PP	GWh	0	151	302	476	504	509	514	535
	Hard Coal PP	GWh	13732	16014	3633	2	1	1	0	0
	Internal Combustion Generator (ICG)	GWh	1015	1345	12	0	0	0	0	0
	Multi-fuel Internal Combustion Generator (ICG)	GWh	0	0	437	437	620	630	669	707
	Nuclear PP	GWh	0	0	0	0	0	0	0	0
	Battery storage output	TWh	0	0	1	8	11	18	28	35
	Pumped hydro energy storage (PHES) output	TWh	0	0	0	0	0	0	0	0
	Thermal Energy Storage (TES) output	TWh	0	0	0	0	0	0	0	0
	Adiabatic Compressed Air Energy Storage (A-CAES) output	TWh	0	0	0	0	0	0	0	0
	Gas storage output	TWh	0	0	0	1	1	1	1	1
**Tamil [illegible]**	PV prosumers	GWh	0	1248	13350	31570	56509	84059	112315	148957
	PV fixed tilted	GWh	225	5519	5519	5520	5520	5520	5520	5453
	PV single-axis tracking	GWh	0	0	26578	70775	70775	101334	149928	180382

	Concentrating Solar Thermal Power (CSP)	GWh	0	0	0	0	0	0	0	0
	Wind energy	GWh	19134	23832	48061	66624	78530	103139	97262	103654
	Hydropower Run-of-River	GWh	233	255	255	255	255	255	255	255
	Hydropower reservoir (dam)	GWh	4433	4433	4433	6626	6650	6650	6650	6650
	Geothermal power	GWh	0	0	0	0	0	0	0	0
	Power-to-Gas	$GWhe_l$	0	0	6	2553	3290	5054	7979	9605
	Combined Cycle Gas Turbine (CCGT)	GWh	4895	898	996	996	25	66	175	11
	Open Cycle Gas Turbine (OCGT)	GWh	138	0	28	28	0	1	1	553
	Heavy Duty Open Cycle Gas Turbine (OCGT HD)	GWh	0	0	7	7	1	2	7	258
	Biomass PP	GWh	1696	5203	8691	12389	12597	12597	12736	12944
	Waste-to-Power	GWh	0	145	290	449	460	461	461	461
	Biogas PP	GWh	0	449	898	1419	1502	1514	1547	1585
	Hard Coal PP	GWh	56370	82017	48992	1301	2007	959	537	0
	Internal Combustion Generator (ICG)	GWh	19049	4455	1725	1532	0	0	0	0
	Multi-fuel Internal Combustion Generator (ICG)	GWh	0	0	1442	1442	2422	3114	4197	4347
	Nuclear PP	GWh	7342	18168	18168	16530	14892	14892	14892	14892
	Battery storage output	TWh	0	0	6	32	45	65	91	112
	Pumped hydro energy storage (PHES) output	TWh	0	0	2	1	1	1	1	1
	Thermal Energy Storage (TES) output	TWh	0	0	0	0	0	0	0	0
	Adiabatic Compressed Air Energy Storage (A-CAES) output	TWh	0	0	0	0	0	0	0	0
	Gas storage output	TWh	0	1	1	3	4	5	7	8
**Karnataka**	PV prosumers	GWh	0	870	9101	23545	39386	59536	79039	104862
	PV fixed tilted	GWh	123	11047	11047	11047	11047	54055	110768	237960
	PV single-axis tracking	GWh	0	0	20606	78364	78365	78380	112122	112141
	Concentrating Solar Thermal Power (CSP)	GWh	0	0	0	0	0	0	0	0
	Wind energy	GWh	6771	12200	34639	36786	37989	35909	33830	27173
	Hydropower Run-of-River	GWh	3104	3160	3160	3160	3160	3160	3160	3160
	Hydropower reservoir (dam)	GWh	12872	12872	19307	19308	19308	19308	19308	19308
	Geothermal power	GWh	0	0	0	0	0	0	0	0
	Power-to-Gas	$GWhe_l$	0	0	6	1296	1679	2685	4999	5416
	Combined Cycle Gas Turbine (CCGT)	GWh	1293	295	263	263	8	9	159	247
	Open Cycle Gas Turbine (OCGT)	GWh	212	1	43	43	0	1	1	28
	Heavy Duty Open Cycle Gas Turbine (OCGT HD)	GWh	0	0	6	7	1	2	5	116
	Biomass PP	GWh	1696	5257	8798	12552	12763	12763	12903	13114
	Waste-to-Power	GWh	0	312	624	964	989	992	992	992
	Biogas PP	GWh	0	314	628	992	1050	1058	1075	1111
	Hard Coal PP	GWh	31740	43329	11402	3	2	2	0	0
	Internal Combustion Generator (ICG)	GWh	2413	383	160	145	0	0	0	0
	Multi-fuel Internal Combustion Generator (ICG)	GWh	0	0	1130	1130	1402	1836	2630	2608
	Nuclear PP	GWh	4487	6552	6552	6552	6552	6552	4914	3276

	Battery storage output	TWh	0	0	4	31	48	81	137	217
	Pumped hydro energy storage (PHES) output	TWh	0	0	0	0	0	0	0	0
	Thermal Energy Storage (TES) output	TWh	0	0	0	0	0	0	0	0
	Adiabatic Compressed Air Energy Storage (A-CAES) output	TWh	0	0	0	0	0	0	0	0
	Gas storage output	TWh	0	0	1	2	2	3	4	5
**Andhra Pradesh**	PV prosumers	GWh	0	654	7219	19043	32099	48943	65912	88273
	PV fixed tilted	GWh	378	5308	5308	5308	5308	5518	45407	92638
	PV single-axis tracking	GWh	0	0	16736	65750	65750	65751	65768	65770
	Concentrating Solar Thermal Power (CSP)	GWh	0	0	0	0	0	0	0	0
	Wind energy	GWh	2649	10504	31114	43636	46718	46422	45608	57738
	Hydropower Run-of-River	GWh	333	333	333	333	333	333	333	333
	Hydropower reservoir (dam)	GWh	6246	6425	9636	9637	9637	9637	9637	9637
	Geothermal power	GWh	0	0	0	0	0	0	0	0
	Power-to-Gas	$GWhe_l$	0	0	6	2453	3061	2981	7440	7526
	Combined Cycle Gas Turbine (CCGT)	GWh	10294	1579	2685	2685	156	386	1605	1997
	Open Cycle Gas Turbine (OCGT)	GWh	1088	0	288	283	0	1	1	26
	Heavy Duty Open Cycle Gas Turbine (OCGT HD)	GWh	0	0	7	7	2	2	3	66
	Biomass PP	GWh	1696	3267	4818	6471	6579	6579	6652	6761
	Waste-to-Power	GWh	0	115	230	356	365	366	366	366
	Biogas PP	GWh	0	560	1119	1769	1873	1888	1922	1976
	Hard Coal PP	GWh	35425	54711	24633	1286	47	97	0	0
	Internal Combustion Generator (ICG)	GWh	602	139	22	18	0	0	0	0
	Multi-fuel Internal Combustion Generator (ICG)	GWh	0	0	996	996	2275	2034	3013	2572
	Nuclear PP	GWh	0	0	0	0	0	0	0	0
	Battery storage output	TWh	0	0	3	26	35	46	71	97
	Pumped hydro energy storage (PHES) output	TWh	0	0	0	0	0	0	0	0
	Thermal Energy Storage (TES) output	TWh	0	0	0	0	0	0	0	0
	Adiabatic Compressed Air Energy Storage (A-CAES) output	TWh	0	0	0	0	0	0	0	0
	Gas storage output	TWh	0	1	2	4	4	4	7	7
**Telangana**	PV prosumers	GWh	0	620	6868	18159	30759	46573	64583	88036
	PV fixed tilted	GWh	98	5491	5491	5491	5491	33335	75754	137690
	PV single-axis tracking	GWh	0	0	23275	80319	80319	80322	80328	80333
	Concentrating Solar Thermal Power (CSP)	GWh	0	0	0	0	0	0	0	0
	Wind energy	GWh	0	329	39185	41616	45330	45330	45330	44926
	Hydropower Run-of-River	GWh	20	85	85	85	85	85	85	85
	Hydropower reservoir (dam)	GWh	2570	3096	3096	3096	3096	3096	3096	3102
	Geothermal power	GWh	0	0	0	0	0	0	0	0
	Power-to-Gas	$GWhe_l$	0	0	6	1987	3472	5141	7185	7054
	Combined Cycle Gas Turbine (CCGT)	GWh	1843	563	437	375	10	91	273	260
	Open Cycle Gas Turbine (OCGT)	GWh	0	0	2	2	0	1	0	37

	Heavy Duty Open Cycle Gas Turbine (OCGT HD)	GWh	0	0	6	6	1	2	5	275
	Biomass PP	GWh	1696	2615	3514	4478	4553	4553	4604	4679
	Waste-to-Power	GWh	0	50	99	153	157	158	158	158
	Biogas PP	GWh	0	41	83	130	138	139	141	151
	Hard Coal PP	GWh	34860	61839	22197	2067	600	251	17	0
	Internal Combustion Generator (ICG)	GWh	0	0	0	0	0	0	0	0
	Multi-fuel Internal Combustion Generator (ICG)	GWh	0	0	1015	1015	1665	2332	3090	2659
	Nuclear PP	GWh	0	0	0	0	0	0	0	0
	Battery storage output	TWh	0	0	3	25	35	60	89	132
	Pumped hydro energy storage (PHES) output	TWh	0	0	3	3	3	3	3	3
	Thermal Energy Storage (TES) output	TWh	0	0	0	0	0	0	0	0
	Adiabatic Compressed Air Energy Storage (A-CAES) output	TWh	0	0	0	0	0	0	0	0
	Gas storage output	TWh	0	0	0	1	2	3	5	5
**Odisha**	PV prosumers	GWh	0	296	3250	8539	16170	24859	34255	47202
	PV fixed tilted	GWh	49	583	583	583	30941	49828	50117	81181
	PV single-axis tracking	GWh	0	0	0	9122	35956	35956	52237	52238
	Concentrating Solar Thermal Power (CSP)	GWh	0	0	0	0	0	0	0	0
	Wind energy	GWh	0	0	3019	3206	3492	3493	3493	3493
	Hydropower Run-of-River	GWh	165	165	165	165	165	165	165	165
	Hydropower reservoir (dam)	GWh	5609	5609	5609	8414	8414	8414	8414	8414
	Geothermal power	GWh	0	0	0	0	0	0	0	0
	Power-to-Gas	GWh$_{el}$	0	0	1	3	565	589	1033	11106
	Combined Cycle Gas Turbine (CCGT)	GWh	0	0	1	1	1	2	2	2
	Open Cycle Gas Turbine (OCGT)	GWh	21	61	75	75	1	6	104	171
	Heavy Duty Open Cycle Gas Turbine (OCGT HD)	GWh	0	0	1	1	1	3	3	4
	Biomass PP	GWh	57	524	3155	4803	4856	4883	4883	4896
	Waste-to-Power	GWh	0	288	576	895	917	917	917	917
	Biogas PP	GWh	0	148	295	462	488	501	520	534
	Hard Coal PP	GWh	75702	33024	31255	4157	1413	861	954	0
	Internal Combustion Generator (ICG)	GWh	173	0	0	0	0	0	0	0
	Multi-fuel Internal Combustion Generator (ICG)	GWh	0	0	77	172	508	515	556	4684
	Nuclear PP	GWh	0	0	0	0	0	0	0	0
	Battery storage output	TWh	0	0	1	7	28	40	50	72
	Pumped hydro energy storage (PHES) output	TWh	0	0	0	0	0	0	0	0
	Thermal Energy Storage (TES) output	TWh	0	0	0	0	0	0	0	0
	Adiabatic Compressed Air Energy Storage (A-CAES) output	TWh	0	0	0	0	0	0	0	0
	Gas storage output	TWh	0	0	0	1	1	1	1	8
	PV prosumers	GWh	0	840	8388	21738	37566	58073	80247	106532
	PV fixed tilted	GWh	11	103	103	125	29030	52619	84884	118122
	PV single-axis tracking	GWh	0	0	0	31201	31201	31202	31203	31205

West Bengal & Sikkim	Concentrating Solar Thermal Power (CSP)	GWh	0	0	0	0	0	0	0	0
	Wind energy	GWh	0	0	15646	29651	32297	32297	32297	32297
	Hydropower Run-of-River	GWh	3759	10872	10872	10872	10872	10872	10872	10872
	Hydropower reservoir (dam)	GWh	4576	4576	6864	6864	6864	6864	6864	6864
	Geothermal power	GWh	0	0	10627	10627	10627	10627	10627	10627
	Power-to-Gas	$GWhe_l$	0	0	1	330	5484	5838	6427	6715
	Combined Cycle Gas Turbine (CCGT)	GWh	140	141	28	28	51	94	93	91
	Open Cycle Gas Turbine (OCGT)	GWh	289	13	6	6	0	1	9	394
	Heavy Duty Open Cycle Gas Turbine (OCGT HD)	GWh	0	0	1	1	1	2	3	3
	Biomass PP	GWh	57	524	3699	5637	5699	5730	5730	5745
	Waste-to-Power	GWh	0	61	123	191	196	196	196	196
	Biogas PP	GWh	0	263	525	821	868	891	927	951
	Hard Coal PP	GWh	62341	69933	48943	8060	463	462	431	0
	Internal Combustion Generator (ICG)	GWh	21	0	2	2	0	0	0	0
	Multi-fuel Internal Combustion Generator (ICG)	GWh	0	0	832	832	2786	3004	3217	2838
	Nuclear PP	GWh	0	0	0	0	0	0	0	0
	Battery storage output	TWh	0	0	3	17	35	55	80	107
	Pumped hydro energy storage (PHES) output	TWh	0	0	0	0	0	0	0	0
	Thermal Energy Storage (TES) output	TWh	0	0	0	0	0	0	0	0
	Adiabatic Compressed Air Energy Storage (A-CAES) output	TWh	0	0	0	0	0	0	0	0
	Gas storage output	TWh	0	0	1	1	5	5	5	5
Jharkhand	PV prosumers	GWh	0	186	1665	5359	9966	15003	20932	29461
	PV fixed tilted	GWh	25	30	30	30	25436	33635	47330	74542
	PV single-axis tracking	GWh	0	0	0	2162	2165	2166	2168	2168
	Concentrating Solar Thermal Power (CSP)	GWh	0	0	0	0	0	0	0	0
	Wind energy	GWh	0	0	6779	9976	10874	10874	10875	10875
	Hydropower Run-of-River	GWh	0	0	0	0	0	0	0	0
	Hydropower reservoir (dam)	GWh	0	0	0	0	0	0	0	0
	Geothermal power	GWh	0	0	0	0	0	0	0	0
	Power-to-Gas	$GWhe_l$	0	0	1	937	6316	7185	7628	7624
	Combined Cycle Gas Turbine (CCGT)	GWh	0	0	1	1	1	2	2	2
	Open Cycle Gas Turbine (OCGT)	GWh	0	0	1	1	0	0	1	1
	Heavy Duty Open Cycle Gas Turbine (OCGT HD)	GWh	0	0	1	1	1	2	3	2
	Biomass PP	GWh	57	427	796	1190	1203	1209	1209	1212
	Waste-to-Power	GWh	0	154	307	478	490	490	490	490
	Biogas PP	GWh	0	131	178	278	294	302	310	322
	Hard Coal PP	GWh	12061	20191	17174	3654	156	155	82	0
	Internal Combustion Generator (ICG)	GWh	0	0	0	0	0	0	0	0
	Multi-fuel Internal Combustion Generator (ICG)	GWh	0	0	1043	1043	2767	3345	3433	3287
	Nuclear PP	GWh	0	0	0	0	0	0	0	0

	Battery storage output	TWh	0	0	0	5	12	17	27	44
	Pumped hydro energy storage (PHES) output	TWh	0	0	0	0	0	0	0	0
	Thermal Energy Storage (TES) output	TWh	0	0	0	0	0	0	0	0
	Adiabatic Compressed Air Energy Storage (A-CAES) output	TWh	0	0	0	0	0	0	0	0
	Gas storage output	TWh	0	0	0	1	4	5	5	5
**Bihar**	PV prosumers	GWh	0	278	2669	7456	13719	21110	29963	40652
	PV fixed tilted	GWh	0	224	224	224	13483	18821	22558	42612
	PV single-axis tracking	GWh	0	0	0	4984	4986	4986	13330	13330
	Concentrating Solar Thermal Power (CSP)	GWh	0	0	0	0	0	0	0	0
	Wind energy	GWh	0	0	7331	7786	8485	8485	8485	8485
	Hydropower Run-of-River	GWh	329	329	493	493	493	493	493	493
	Hydropower reservoir (dam)	GWh	0	0	0	0	0	0	0	0
	Geothermal power	GWh	0	0	10627	10627	10627	10627	10627	10627
	Power-to-Gas	$GWhe_l$	0	0	1	3	696	888	1004	1068
	Combined Cycle Gas Turbine (CCGT)	GWh	0	0	1	1	1	1	2	2
	Open Cycle Gas Turbine (OCGT)	GWh	0	0	1	1	0	0	1	1
	Heavy Duty Open Cycle Gas Turbine (OCGT HD)	GWh	0	0	1	1	1	2	3	2
	Biomass PP	GWh	57	524	4400	6710	6784	6821	6821	6840
	Waste-to-Power	GWh	0	39	79	122	125	125	125	125
	Biogas PP	GWh	0	277	377	589	622	639	650	682
	Hard Coal PP	GWh	19949	44984	17621	429	3	21	102	0
	Internal Combustion Generator (ICG)	GWh	0	0	0	0	0	0	0	0
	Multi-fuel Internal Combustion Generator (ICG)	GWh	0	0	242	365	651	752	788	800
	Nuclear PP	GWh	0	0	0	0	0	0	0	0
	Battery storage output	TWh	0	0	1	7	12	16	25	40
	Pumped hydro energy storage (PHES) output	TWh	0	0	0	0	0	0	0	0
	Thermal Energy Storage (TES) output	TWh	0	0	0	0	0	0	0	0
	Adiabatic Compressed Air Energy Storage (A-CAES) output	TWh	0	0	0	0	0	0	0	0
	Gas storage output	TWh	0	0	1	1	1	1	1	1
**Northeastern states**	PV prosumers	GWh	0	197	1982	5306	9033	13595	18053	24073
	PV fixed tilted	GWh	8	26	26	26	1263	6252	6421	16726
	PV single-axis tracking	GWh	0	0	0	0	1	2	6863	6864
	Concentrating Solar Thermal Power (CSP)	GWh	0	0	0	0	0	0	0	0
	Wind energy	GWh	0	0	2128	4668	5085	5085	5085	5085
	Hydropower Run-of-River	GWh	1757	2521	2521	2521	2521	2521	2521	2521
	Hydropower reservoir (dam)	GWh	2546	2546	2547	3819	3819	3819	3819	3819
	Geothermal power	GWh	0	0	10627	10627	10627	10627	10627	10626
	Power-to-Gas	$GWhe_l$	0	0	1	11	14	86	1889	1959
	Combined Cycle Gas Turbine (CCGT)	GWh	2107	686	721	714	180	216	711	717
	Open Cycle Gas Turbine (OCGT)	GWh	774	0	148	136	0	0	2	6

	Heavy Duty Open Cycle Gas Turbine (OCGT HD)	GWh	0	0	1	1	1	0	1	1
	Biomass PP	GWh	57	524	2440	3708	3749	3770	3770	3780
	Waste-to-Power	GWh	0	602	1203	1870	1917	1917	1917	1917
	Biogas PP	GWh	0	97	194	303	321	329	333	350
	Hard Coal PP	GWh	445	6880	1720	1	0	0	0	0
	Internal Combustion Generator (ICG)	GWh	597	0	68	37	0	0	0	0
	Multi-fuel Internal Combustion Generator (ICG)	GWh	0	0	2	3	5	16	395	464
	Nuclear PP	GWh	0	0	0	0	0	0	0	0
	Battery storage output	TWh	0	0	1	2	5	8	12	18
	Pumped hydro energy storage (PHES) output	TWh	0	0	0	0	0	0	0	0
	Thermal Energy Storage (TES) output	TWh	0	0	0	0	0	0	0	0
	Adiabatic Compressed Air Energy Storage (A-CAES) output	TWh	0	0	0	0	0	0	0	0
	Gas storage output	TWh	0	0	0	0	0	0	2	2

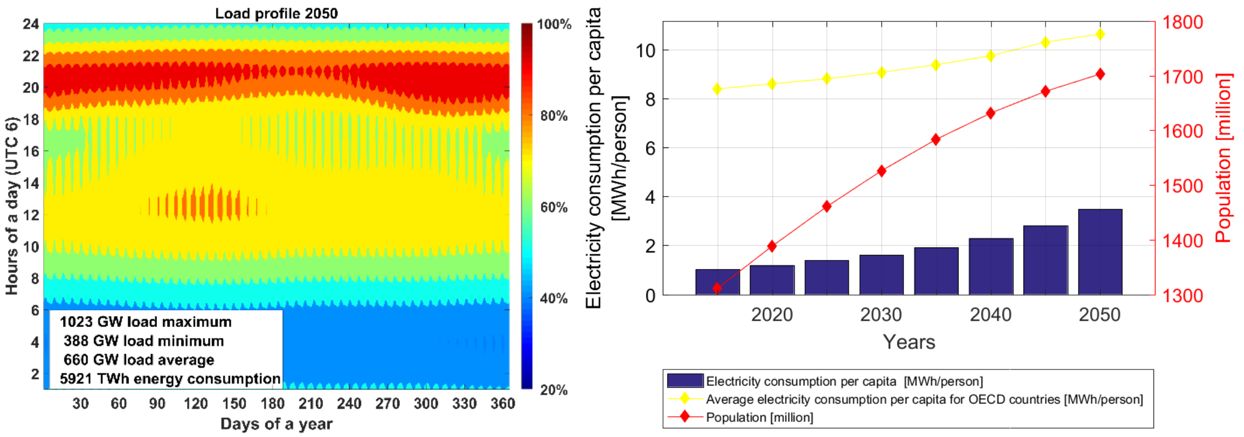

Supplementary Figure 1: The synthetic load profile in 2050 (left) [31] and the average electricity consumption per capita in India and in OECD countries, growth in population from 2015 to 2050 (right).

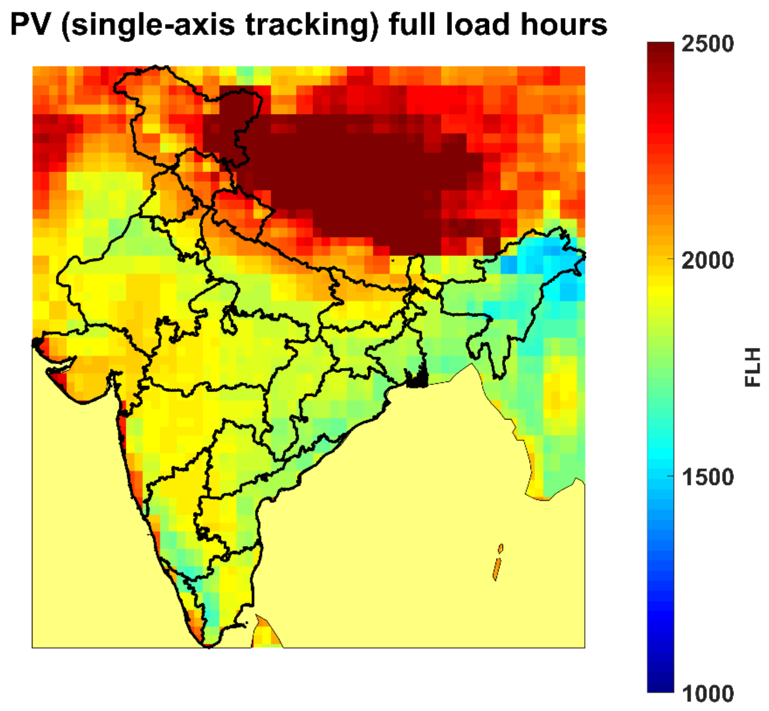

Supplementary Figure 2: Annual full load hours (FLH) across India for solar PV single-axis tracking. Also, called as capacity factor, it ranges between 17% - 25% across the different states.

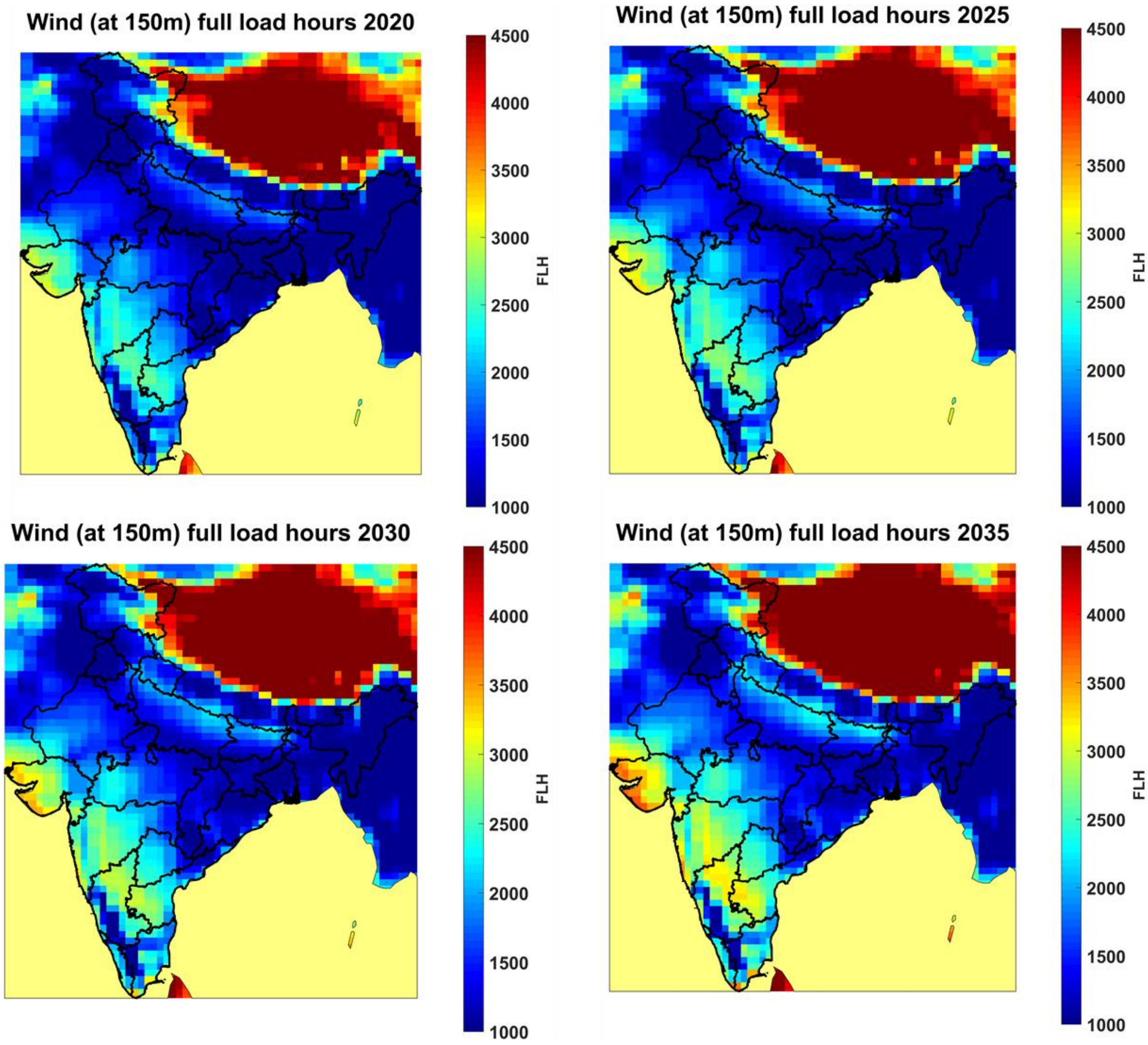

Supplementary Figure 3: Annual full load hours (FLH) across India for onshore wind. An approach of increasing capacity factors is applied to capture the advancement in turbine technology. The average annual capacity factor in 2020 is 29%, 2025 is 31%, 2030 is 33% and from 2035 onwards 36% is assumed.

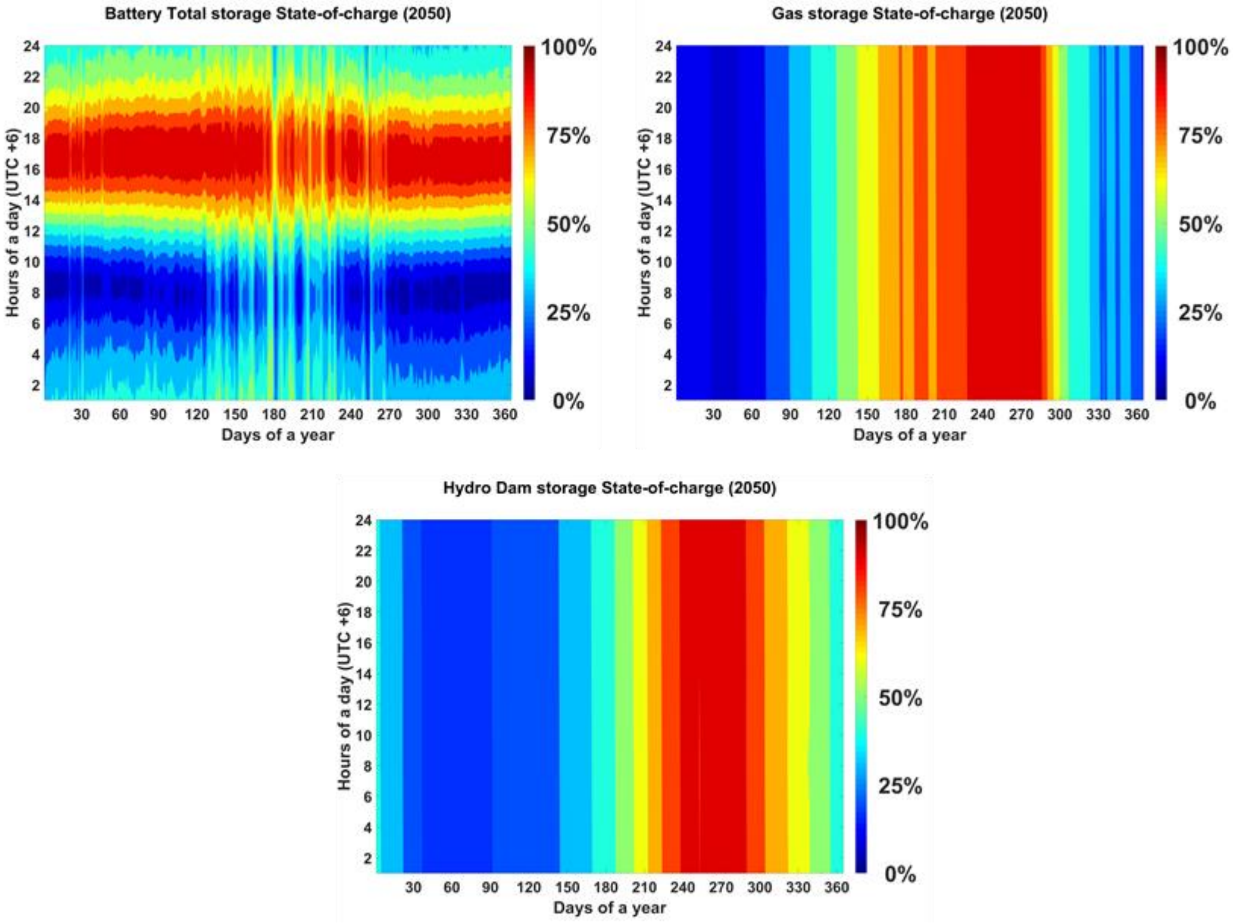

Supplementary Figure 4: State of charge for battery storage (left) and gas storage (right) in 2050 and hydropower dam (bottom) across India.

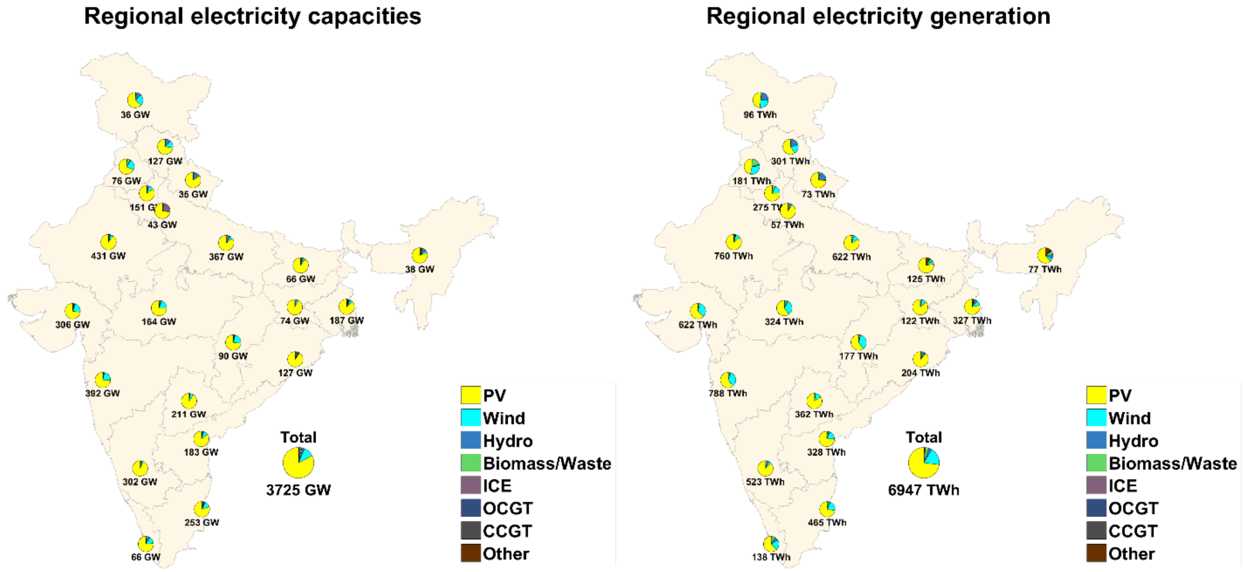

Supplementary Figure 5: Regional electricity generation capacities (left) and electricity generation (right) across India in 2050.

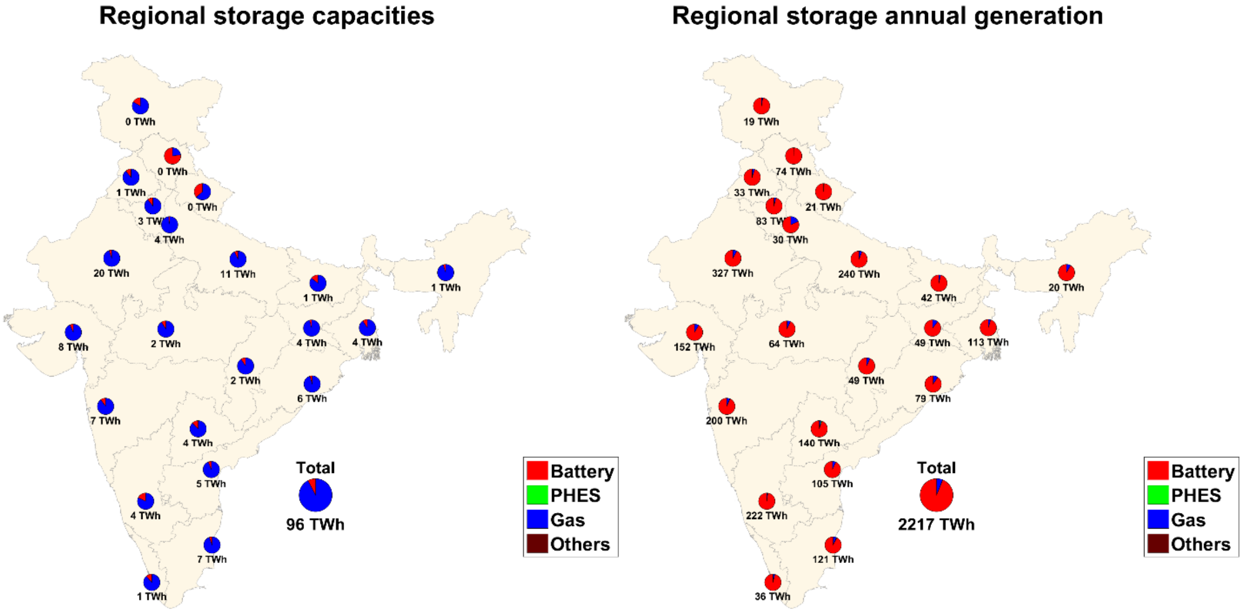

Supplementary Figure 6: Regional distribution of storage capacities (left) and output (right) across India in 2050.

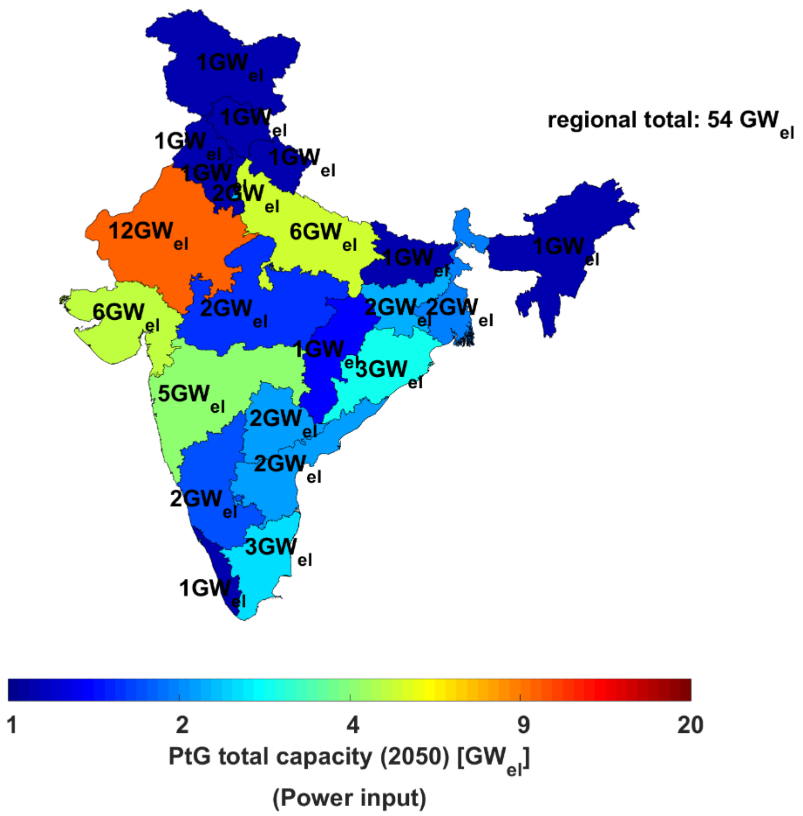

Supplementary Figure 7: Regional distribution of installed capacity of electrolysers and CO_2 direct air capture across India in 2050.

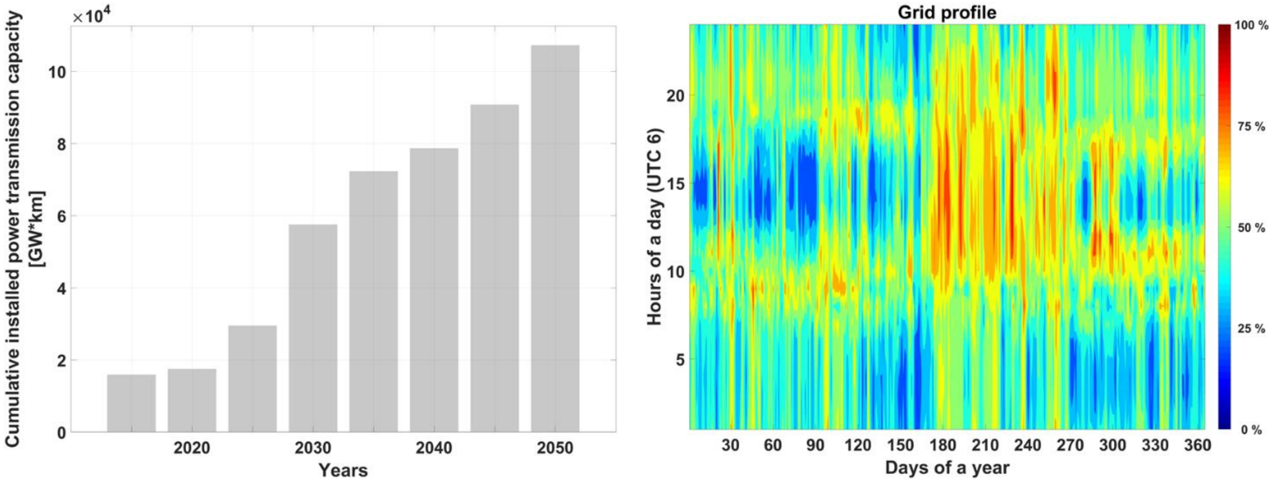

Supplementary Figure 8: Total cumulative grid capacity (left) installed during the transition and hourly profile of grid utilisation across India in 2050 (right).

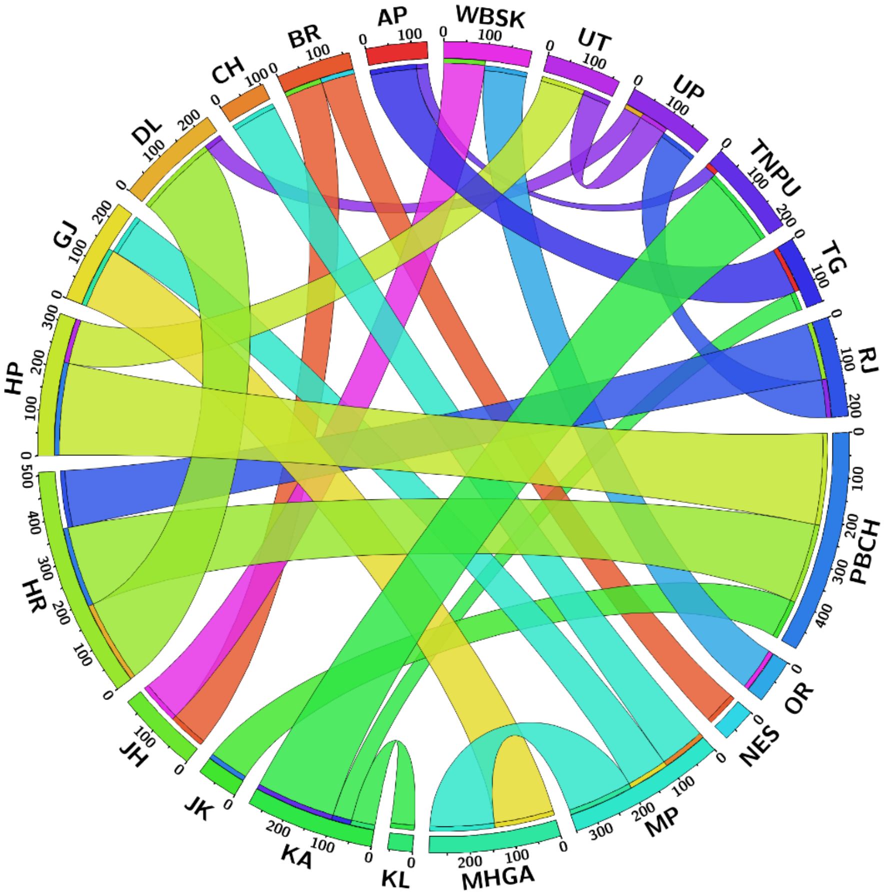

Supplementary Figure 9: Interregional exchange of electricity across India in 2050. The thickness of the flow indicates the amount of electricity exchanged between the regions in TWh. The exporter region's outer ribbon and the flow associated with it have the same colour.

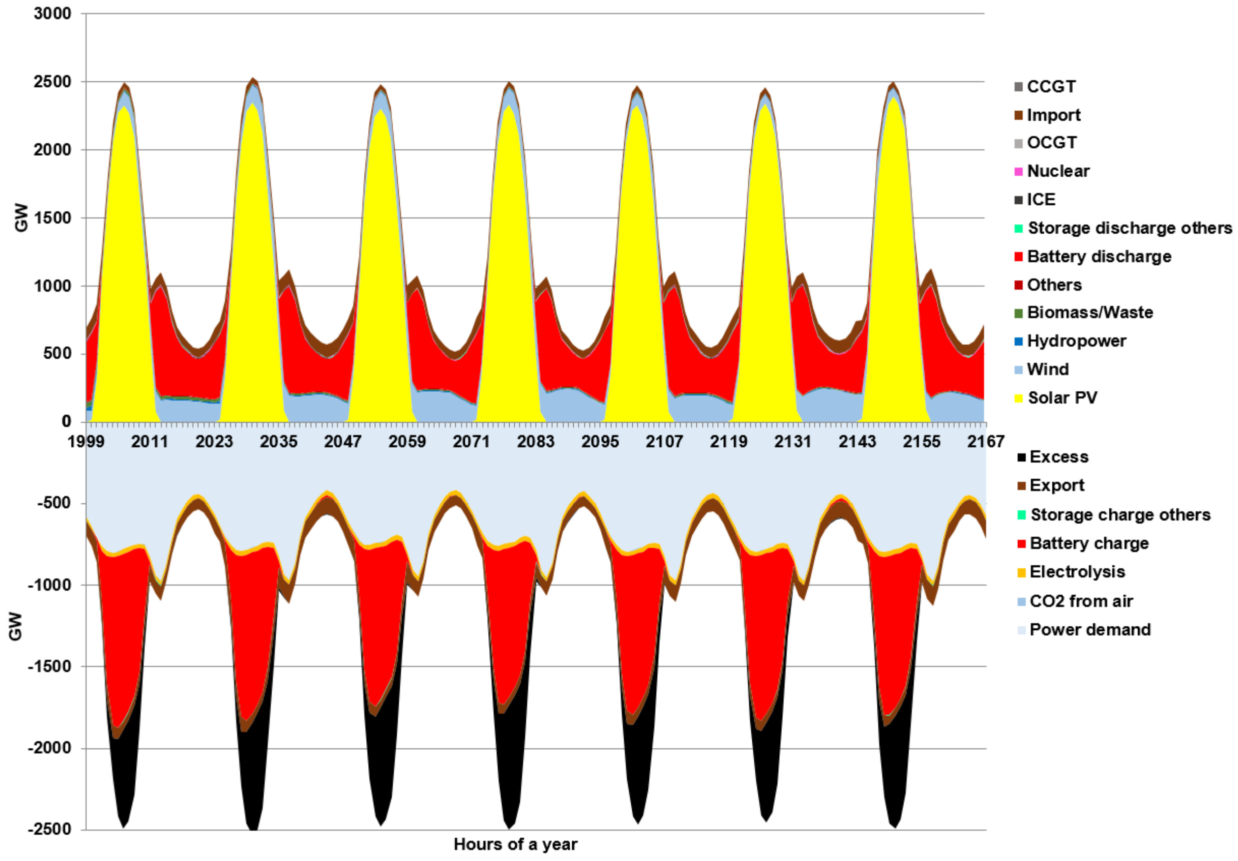
3000
2500
2000
1500
1000
500
0
-500
-1000
-1500
-2000
-2500
GW
GW
1999
2011
2023
2035
2047
2059
2071
2083
2095
2107
2119
2131
2143
2155
2167
Hours of a year
CCGT
Import
OCGT
Nuclear
ICE
Storage discharge others
Battery discharge
Others
Biomass/Waste
Hydropower
Wind
Solar PV
Excess
Export
Storage charge others
Battery charge
Electrolysis
CO2 from air
Power demand

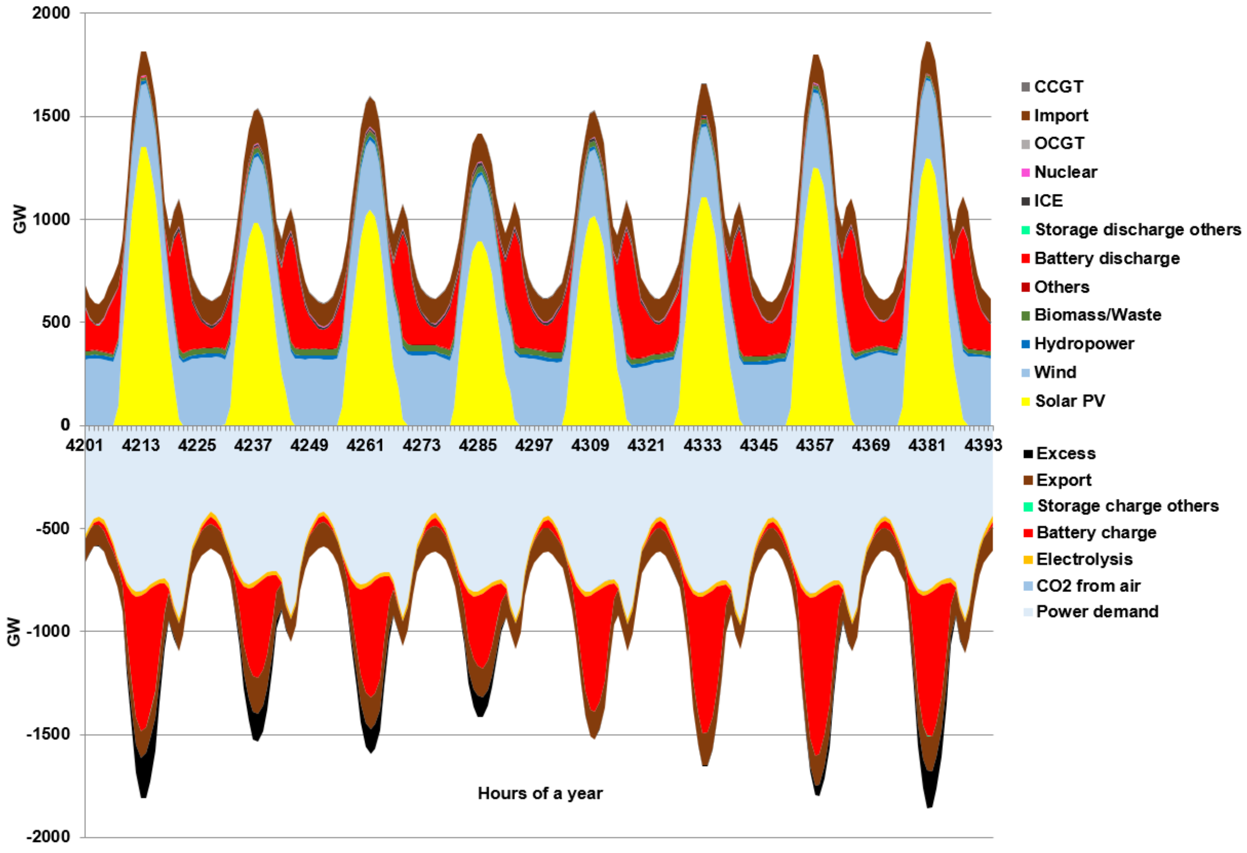
2000
1500
1000
500
0
-500
-1000
-1500
-2000
GW
GW
4201
4213
4225
4237
4249
4261
4273
4285
4297
4309
4321
4333
4345
4357
4369
4381
4393
Hours of a year
CCGT
Import
OCGT
Nuclear
ICE
Storage discharge others
Battery discharge
Others
Biomass/Waste
Hydropower
Wind
Solar PV
Excess
Export
Storage charge others
Battery charge
Electrolysis
CO2 from air
Power demand

Supplementary Figure 10: Hourly operation of the Indian power system during a best solar week in 2050 (top) and worst solar week (bottom).

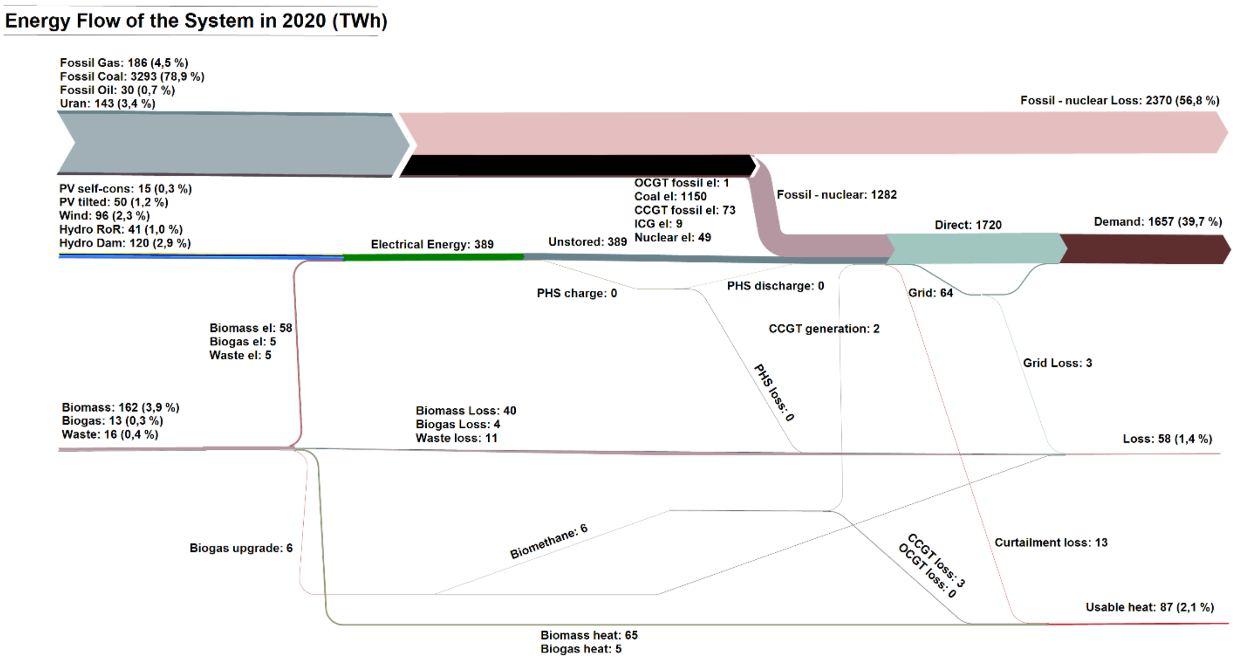

Supplementary Figure 11: Energy flows of the Indian power system in 2020.

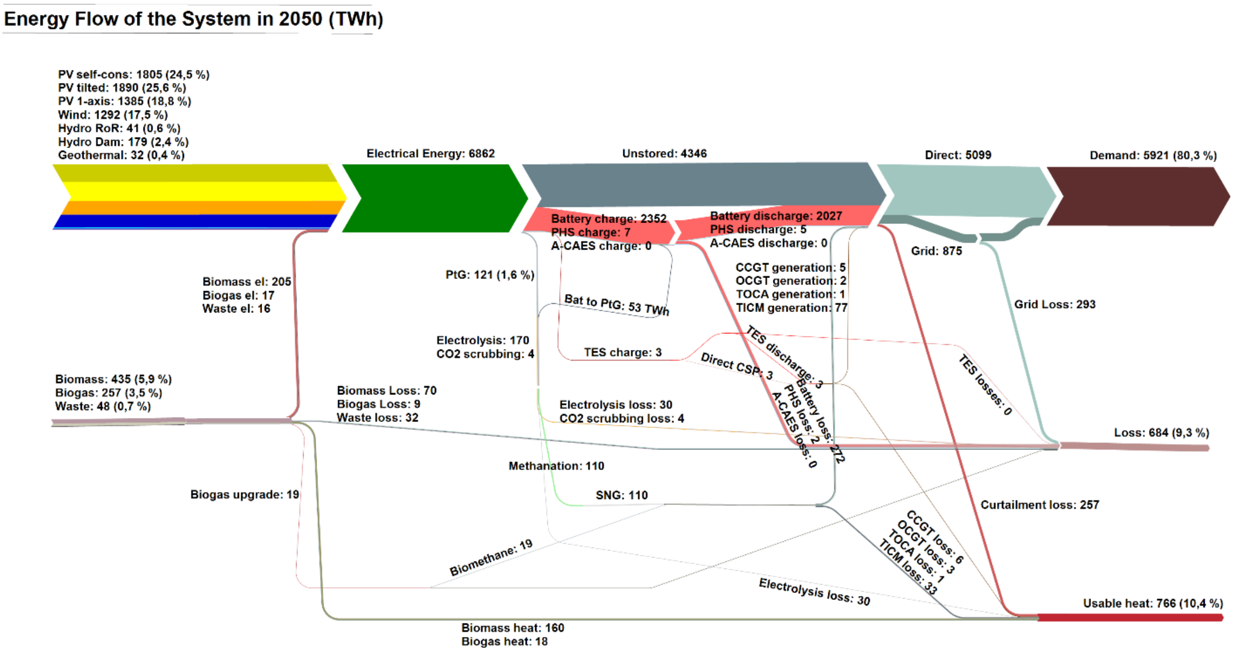

Supplementary Figure 12: Energy flows of the Indian power system in 2050.

Abbreviations

A-CAES	Adiabatic compressed air energy storage
CAPEX	Capital expenditures
CCGT	Combined cycle gas turbine
CHP	Combined heat and power
CSP	Concentrated solar thermal power
GHG	Greenhouse gas
GW	Gigawatt
HVAC	High voltage alternating current
HVDC	High voltage direct current
MW	Megawatt
OCGT	Open cycle gas turbine
OCGT HD	Open cycle gas turbine – Heavy Duty
OPEX	Operational expenditures
PHES	Pumped hydro energy storage
PP	Power plant
PV	Photovoltaics
TES	Thermal energy storage

References

1. Central Electricity Authority (CEA). *National Electrcity Plan*. http://www.cea.nic.in/reports/committee/nep/nep_jan_2018.pdf (2018).

2. Central Electricity Regulatory Commission. *Tariff Determination from Renewable Energy Sources - Regulations*. http://www.cercind.gov.in/2017/regulation/Noti131.pdf (2017).

3. Bolinger, Mark; Seel, J. *Utility-Scale Solar 2015: An Empirical Analysis of Project Cost, Performance, and Pricing Trends in the United States. Lawrence Berkeley National Laboratory*. (2016).

4. European Commission. Joint Research Centre. Institute for Energy and Transport. & SERTIS. *Energy Technology Reference Indicator (ETRI) projections for 2010-2050.* (2014).

5. Sigfússon, B. & Uihlein, A. *2015 JRC Geothermal Energy Status Report. European Commission - Joint Research Centre*. https://publications.jrc.ec.europa.eu/repository/bitstream/JRC99264/2015 jrc geothermal energy status report_online.pdf (2015) doi:10.2790/959587.

6. Central Electricity Authority (CEA). *DRAFT REPORT ON OPTIMAL GENERATION CAPACITY MIX FOR 2029-30*. (2019).

7. Koomey, J. & Hultman, N. E. A reactor-level analysis of busbar costs for US nuclear plants, 1970-2005. *Energy Policy* **35**, 5630–5642 (2007).

8. [IEA]- International Energy Agency. *World Energy Outlook*. https://www.iea.org/reports/world-energy-outlook-2017 (2017).

9. [IEA] - International Energy Agency and [NEA]-Nuclear Energy Agency. *Projected costs of generating electricity*. (2015) doi:10.1787/cost_electricity-2015-en.

10. Central Electricity Regulatory Commission. *Tariff Regulations*. (2019).

11. Lazard. *Lazard's Levelised Cost of Energy Analysis (version 10.0)*. (2016).

12. Urban, W., Lohmann, H. & Girod, K. Abschlussbericht für das BMBF-Verbundprojekt Biogaseinspeisung. *Fraunhofer UMSICHT* (2009).

13. Haysom, J. E., Jafarieh, O., Anis, H., Hinzer, K. & Wright, D. Learning curve analysis of concentrated photovoltaic systems. *Prog. Photovoltaics Res. Appl.* **23**, 1678–1686 (2015).

14. Kutscher, C., Mehos, M., Turchi, C., Glatzmaier, G. & Moss, T. *Line-Focus Solar Power Plant Cost Reduction Plan. National Renewable Energy Laboratory (NREL)*. vol. NREL/TP-55 (2010).

15. Agora Energiewende. *Stromspeicher in der energiewende*. www.agora-energiewende.de/fileadmin/Projekte/2013/%0Aspeicher-in-der-energiewende/Agora_Speicherstudie_Web.pdf. (2014).

16. Breyer, C., Tsupari, E., Tikka, V. & Vainikka, P. Power-to-gas as an emerging profitable business through creating an integrated value chain. in *Energy Procedia* vol. 73 182–189 (2015).

17. Fasihi, M., Efimova, O. & Breyer, C. Techno-economic assessment of CO2 direct air capture plants. *J. Clean. Prod.* **224**, 957–980 (2019).

18. Fasihi, M., Bogdanov, D. & Breyer, C. Long-Term Hydrocarbon Trade Options for the Maghreb Region and Europe—Renewable Energy Based Synthetic Fuels for a Net Zero Emissions World. *Sustainability* **9**, 306 (2017).

19. Hoffmann, W. Importance and evidence for cost effective electricity storage. *29th Eur. PV Sol.*

Energy Conf. Exhib. 3981–3988 (2014) doi:10.1016/j.enpol.2012.11.033.

20. Svensson, R., Odenberger, M., Johnsson, F. & Ströomberg, L. Transportation systems for CO2—application to carbon capture and storage. *Energy Convers. Manag.* **45**, 2343–2353 (2004).

21. Michalski, J. *et al.* Hydrogen generation by electrolysis and storage in salt caverns: Potentials, economics and systems aspects with regard to the German energy transition. *Int. J. Hydrogen Energy* **42**, 13427–13443 (2017).

22. Deutsches Institut für Wirtschaftsforschung. *Data documnetation: Current and Prospective Costs of Electricity Generation until 2050*. (2013).

23. Tongia, R. & Gross, S. *Coal in India -Adjusting to Transition*. (2019).

24. Government of India. *Indian Petroleum and Natural gas statistics*. (2017).

25. McKinsey. Unlocking gas potential - New business models. in *Petrotech* (2019).

26. [BNEF] - Bloomberg New Energy Finance. *New Energy Outlook 2015 - Long-term projections of the global energy sector*. *Bloomberg New Energy Finance* (2015) doi:10.1017/CBO9781107415324.004.

27. [BNEF] - Bloomberg New Energy Finance. *New Energy Outlook 2019*. (2019).

28. CSIRO Division of Atmospheric Research. *Lifecycle emissions and energy analysis of LNG, oil and coal*. (1996).

29. Environmental Protection Agency. *Annexes to the Inventory of U.S. GHG Emissions and Sinks*. (2013).

30. DII. *2050 Desert power—perspectives on a sustainable power system for EUMENA*. (2012).

31. Toktarova, A., Gruber, L., Hlusiak, M., Bogdanov, D. & Breyer, C. Long term load projection in high resolution for all countries globally. *Int. J. Electr. Power Energy Syst.* **111**, 160–181 (2019).
